# Horizontal Transfer of LTR Retrotransposons Contributes to the Genome Diversity of *Vitis*

**DOI:** 10.3390/ijms221910446

**Published:** 2021-09-28

**Authors:** Minkyu Park, Ali Sarkhosh, Violeta Tsolova, Islam El-Sharkawy

**Affiliations:** 1 Center for Viticulture and Small Fruit Research, College of Agriculture and Food Sciences, Florida A&M University, Tallahassee, FL 32308, USA; minkju3003@gmail.com (M.P.); violeta.tsolova@famu.edu (V.T.); 2 Horticultural Sciences Department, University of Florida, Gainesville, FL 32611, USA; sarkhosha@ufl.edu

**Keywords:** genome evolution, horizontal transfer, *Vitis*, genome diversification, LTR retrotransposon

## Abstract

While horizontally transferred transposable elements (TEs) have been reported in several groups of plants, their importance for genome evolution remains poorly understood. To understand how horizontally transferred TEs contribute to plant genome evolution, we investigated the composition and activity of horizontally transferred TEs in the genomes of four *Vitis* species. A total of 35 horizontal transfer (HT) events were identified between the four *Vitis* species and 21 other plant species belonging to 14 different families. We determined the donor and recipient species for 28 of these HTs, with the *Vitis* species being recipients of 15 of them. As a result of HTs, 8–10 LTR retrotransposon clusters were newly formed in the genomes of the four *Vitis* species. The activities of the horizontally acquired LTR retrotransposons differed among *Vitis* species, showing that the consequences of HTs vary during the diversification of the recipient lineage. Our study provides the first evidence that the HT of TEs contributes to the diversification of plant genomes by generating additional TE subfamilies and causing their differential proliferation in host genomes.

## 1. Introduction

Long terminal repeat (LTR) retrotransposons are the major components of plant genomes [1,2], and their activity is an important source of genome evolution [3,4]. The proliferation of LTR retrotransposons can cause genome expansion in both heterochromatic [5] and euchromatic [6] regions. The removal of LTR retrotransposons by unequal homologous recombination and illegitimate recombination can also cause a reduction in genome size [7]. LTR retrotransposons can be activated by stress [8,9], and their activity can mediate the evolution of disease resistance genes [10]. Because plants cannot avoid environmental stress, rapid adaptation and evolution are crucial for their survival and reproduction. The activity of LTR retrotransposons, therefore, plays an important role in plant evolution [11].

LTR retrotransposons are classified into five superfamilies: *Gypsy*, *Copia*, *Bel-Pao*, *Retrovirus*, and *ERV* [12]. Among them, *Gypsy* and *Copia* are the two major superfamilies in plants and the most abundant in plant genomes. The two superfamilies consist of diverse families, and each family can independently increase or decrease in activity [13]. LTR retrotransposons are replicated via an RNA intermediate, and their sequence structure is similar to that of retroviruses except for the absence of the envelope gene that enables extracellular mobility [14]. Because LTR retrotransposons do not have extracellular mobility, the transfer of their genetic materials mainly occurs via vertical transmission from parents to offspring. However, multiple instances of horizontal transfer (HT) of LTR transposons were also reported.

HT refers to the transfer of genetic material across the mating barrier. Although the mechanism of HT and its importance are well known for bacterial genomes [15], they remain poorly studied in eukaryotes. Evidence for HT has thus far been reported in diverse eukaryotes such as fungi [16], insects [17], vertebrates [18,19], and plants [20,21,22]. Transposable elements (TEs) seem to be the most often transferred material among eukaryotes, and it was suggested that such transfers can be important for the diversification of eukaryotic genomes [23]. However, comprehensive studies are required to demonstrate the effects of HTs on the diversification of eukaryotic genomes.

In plants, because genome diversity caused by LTR retrotransposon activity is important for evolution, the HT of LTR retrotransposons is also expected to be important. Although there were several reports on the HT of LTR retrotransposons in plants [20,22,24,25], no direct evidence of their impact on host genome evolution was provided. However, diversification of the TE repertoire by HT and active proliferation of horizontally transferred TEs in the host genome were identified among grass species [22], suggesting that HT can be involved in the diversification of the host genome. In this study, we investigated the composition and activity history of horizontally transferred LTR retrotransposons in four *Vitis* species, and the results provide evidence of plant genome diversification via HTs.

## 2. Results

### 2.1. Investigation of Putative HT Events between Vitis and Other Plant Species

To identify HT cases in the *Vitis* genomes, we used the whole-genome sequences of four *Vitis* species, namely, *V. rotundifolia*, *V. arizonica*, *V. riparia*, and *V. vinifera* [26,27,28,29]. A total of 137 plant whole-genome sequences available from the public genome sequence database (Supplementary Table S1) were used to screen HTs with the *Vitis* genomes. To detect the HTs, we used the following HT screening pipeline (see Methods for details): (1) Perform pairwise comparisons between the four *Vitis* and 137 other plant genome sequences by nucleotide BLAST and isolate candidate sequence contigs containing the hit sequence with >90% identity, (2) remove conserved sequences such as organelle genome sequences, ribosomal sequences, and conserved genes among the candidate sequence contigs, (3) identify candidate HT events by removing redundant HT cases according to homologous copies, and (4) validate each of the candidate HT events by using activity history and phylogenetic analysis of the homologs of the horizontally transferred sequences.

**Figure 1:**
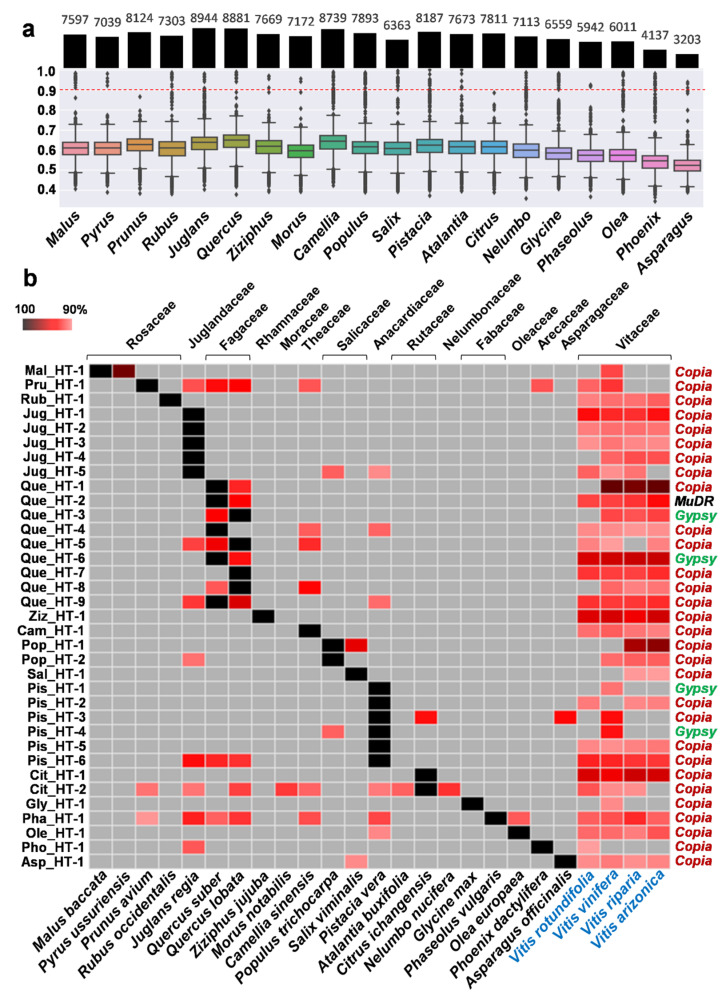
Identification of 35 putative HT events between four *Vitis* and 21 other plant species. (**a**) Box plots reveal the identity distribution of orthologous genes calculated from the 3rd codons. The number of orthologous gene sets used is presented with a bar graph on the top of the box plot panel, and the values are shown at the top of each bar. The genus names are shown at the bottom, and the range for the Y-axis is on the left side of the panel. The 90% identity threshold that was used for the HT investigation is presented by a red dotted line. (**b**) The heat map presents the sequence identity between the sequence of each HT event and that of the plant species involved in the HT. Columns and rows represent the plant species and each HT event, respectively. The names of the plant species are presented at the bottom of the panel, and those of the four *Vitis* species are marked with blue letters. The family names of the species are shown at the top of the panel. The names of the HT events are presented on the left side of the panel, and their classification is on the right. Two LTR retrotransposon superfamilies, *Copia* and *Gypsy*, are marked with red and green letters, respectively, and a DNA transposon, *MuDR*, is marked with black letters. The intensity of the red color in each box reveals identity from 90% (pale red) to 100% (black). The black box appears when the used sequence matches itself. The gray box indicates that there is no match according to the >90% identity threshold.

Through our preliminary tests with the HT screening pipeline, we found that all the sequences for putative HT cases were TEs. To use the activity history and phylogenetic analysis of the TEs for the validation of HT events, we used the homologous sequences of the horizontally transferred TEs in all the involved species. To prevent mixing with other TE family members, a >85% identity threshold was used for the identification of homologous sequences. For the analysis of the activity history of the horizontally transferred TEs, we tested low identity thresholds, including 88%, 90%, and 92%, for the HT investigation, and the 90% identity threshold revealed a distinct difference in the activity history and phylogenetic analyses for the validation of HTs. To verify the adequacy of the threshold, we measured the genetic distance between *Vitis* (*V. vinifera*) and the 20 plant genera that showed HTs in this study by calculating the identity of the 3rd codon of the orthologous gene sets. A total of 3203 to 8944 orthologous gene sets were identified per species, and their identity distribution of the 3rd codon was analyzed with box plots. The maximum of the box plots appeared to be less than 80% (Figure 1a). Outliers with over 90% identity were identified as highly conserved DNAs, including ribosomal proteins, organelle genes, and conserved genes, which were filtered out during the HT investigation. After filtering, no horizontally transferred genes were identified among the HT investigation results obtained with the 90% identity threshold. Considering that protein-coding genes are more conserved than TEs, this indicates that the 90% threshold is high enough to avoid false positives from conserved sequences.

Through the HT screening pipeline, a total of 35 putative HT events were identified between the four *Vitis* species and 21 other plant species belonging to 20 genera and 14 families (Figure 1b and Supplementary Figure S1). All the horizontally transferred DNAs of the 35 HT events were identified as TEs (Figure 1b). Thirty-four out of the 35 events involved LTR retrotransposons, and the rest involved DNA transposons (*MuDR*). Among the 34 horizontally transferred LTR retrotransposons, 30 were *Copia* elements, and the other four were *Gypsy* elements, indicating that 88.2% of the HTs in *Vitis* occurred via *Copia* elements. The alignments between the sequences of the 35 HT events and their homolog sequences in the *Vitis* genome showed that five events involved full-length LTR retrotransposons with the two LTR sequences (Supplementary Figure S2). Among the rest, only four events involved fragments shorter than 2 kb, and the other elements were partially degraded TEs with a length >2 kb.

**Figure 2:**
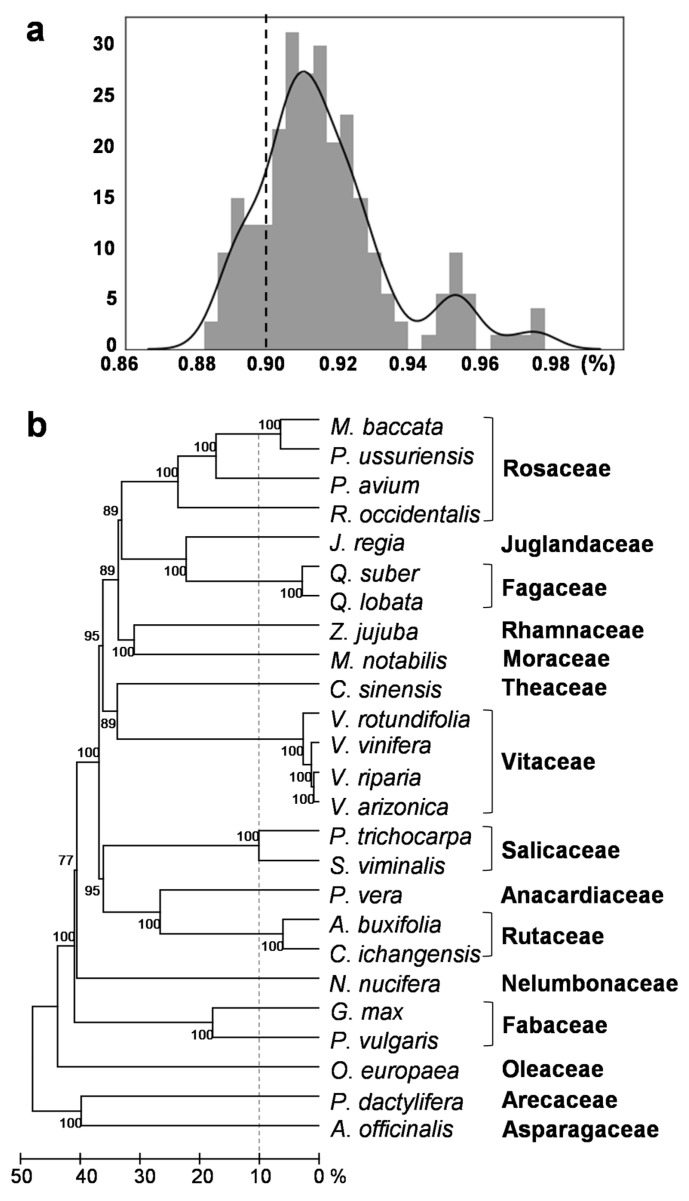
Identity histogram of the HT cases and phylogenetic tree of the 25 plant species involved in the 35 putative HT events. (**a**) The histogram shows the identity frequency of the best hits between the sequences of the 35 putative HT events and the genome sequences of the 25 species involved in the HT events. The Y-axis and X-axis reveal the frequency and identity, respectively. The dotted line indicates the 90% identity threshold used for HT identification. (**b**) The UPGMA phylogenetic tree of the 25 species is presented. The name of the species is shown at the end of each branch, and the family name of the species is presented to the right of the species’ name. The distances among the species are presented with percent identity at the bottom of the phylogenetic tree. The identity at the branching point indicates the species’ distance. The dotted line indicates the 90% identity threshold for the HT investigation.

To investigate additional HTs among the involved species, each of the 35 HT events was investigated among the four *Vitis* and 21 other plant species. For this, the DNA sequences of the 35 putative horizontally transferred fragments were isolated from the 21 plant genome sequences and compared to the four *Vitis* and 21 other plant genome sequences with BLAST. The best hit sequence with a >88% sequence identity was isolated to identify the representative HT event in each species for each of the 35 HT events. The distribution of sequence identity values for all the identified HT events was analyzed with a histogram (Figure 2a). From the histogram, three modes were identified, and the major peak appeared at a 91% identity. The second and third peaks appeared at 95% and 97% identities, respectively. This indicates that most HT events had an approximately 91% identity. Among the identified putative HT events, only those with a >90% identity were used for further analysis (Figure 1b).

To estimate the approximate interspecies distances between the 25 species involved in the HTs, we used pairwise sequence identities calculated for the 3rd codon of 172 orthologous nuclear protein-coding genes. The pairwise distances of the 25 species are presented with an unweighted pair group method with arithmetic mean (UPGMA) phylogenetic tree (Figure 2b, Supplementary Figure S3). Speciation of the two Rosaceae (*Malus baccata* and *Pyrus ussuriensis*), two Fagaceae (*Quercus lobata* and *Quercus suber*), and four Vitaceae species occurred after the 90% identity threshold for HT identification (Figure 2b, dotted line). The pairwise sequence identities between the four *Vitis* species were 97.3–99.1% (Supplementary Figure S3).

### 2.2. Verification of HTs

**Figure 3:**
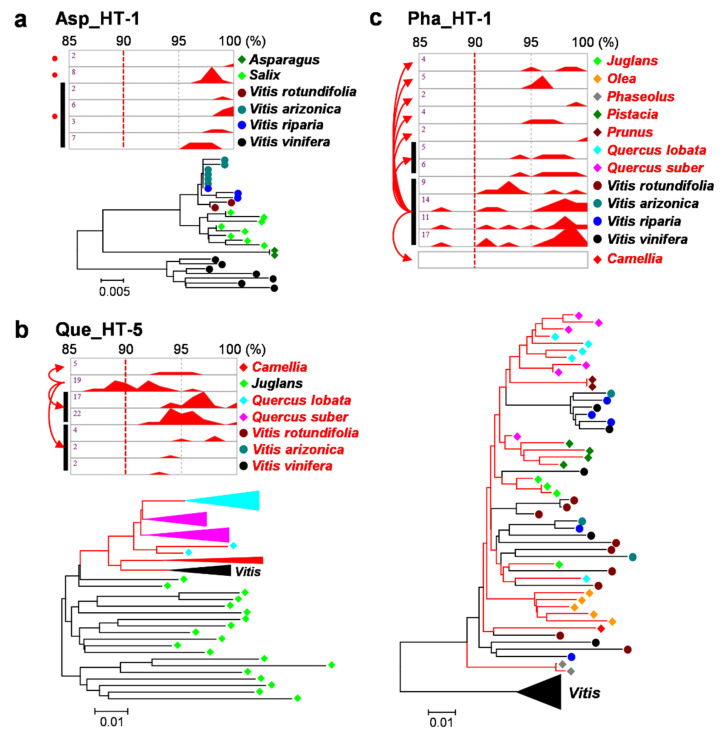
Three representative cases of HT validation. The panels represent cases of “undetermined donor and recipient” (**a**), “*Vitis* as the recipient” (**b**), and “*Vitis* as the donor” (**c**). (**a**) The activity history is shown in the upper panel. The name of the species or genus is shown on the right side of the panel. Each of the *Vitis* species is marked with a colored circle, and other non-*Vitis* species are marked with colored rhombuses. The percent identity is shown at the top of the panel. The red graph in each square represents the relative activity history of the paralogs of the horizontally transferred TE. The numbers in the panel indicate the number of paralogs used in each species. The species in the same genus are marked with black bars on the left side of the panel. The red dots indicate the species or genera for which the donor or recipient was undetermined. The dotted red lines present the 90% identity threshold used in the identification of HTs. The lower panel presents the phylogenetic tree of the homologs of the horizontally transferred TE. The species or genus of each element is marked with the same colored circle or rhombus used in the activity history panel. (**b**) In the activity history panel, the red arrows on the left reveal the direction of HT. In the phylogenetic tree, the branches of recipients are marked with red lines. The colored triangles in the phylogenetic tree represent the merged clusters of the same species or genus. The triangles of the species or genera, except for *Vitis*, are presented in the same color used in the activity history panel. The *Vitis* genus is presented with a black triangle. The rest are the same as in (**a**). (**c**) In the activity history panel, the species that contains a single-copy homolog is presented with an empty square at the bottom of the panel. The rest are the same as in (**a**,**b**).

Each of the 35 putative HT events was verified with activity history and phylogenetic analyses of the homologs of the horizontally transferred TEs. We assumed that if a TE was horizontally transferred and proliferated in the host genome thereafter, the activity history of its paralogs would appear after the HT event. In the case of the donor species, the activity history of the homologs was also expected to appear before the HT event. With the activity history, we used phylogenetic analysis to verify each HT and determine its donor and recipient species. Based on the verification results, the confirmed HT events were classified into three types: the donor and recipients were not determined (Figure 3a), *Vitis* was the recipient (Figure 3b), and *Vitis* was the donor (Figure 3c).

The activity history of the horizontally transferred TEs and their homologs in the donor species was estimated with their paralog sequences in each species. In the case of Asp_HT-1, the TE paralogs of *Asparagus* and *Salix* were nested within their homologs of *Vitis* in the phylogenetic tree (Figure 3a). However, the activity history of the TE paralogs of all the genera exceeded the 90% identity threshold (Figure 3a, red dotted line). This could be interpreted in two ways. One is that all the species are recipients, and the donor species is unknown. The other is that, based on the phylogenetic analysis, *Vitis* is the donor, but the old activity of the TE paralogs in *Vitis* species is not detected by the removal of the old TEs. Therefore, this case was classified as “not determined”. In the case of donor species, the activity history appeared under the 90% identity threshold (Figure 3b, *Juglans* and Figure 3c, *Vitis*). In the phylogenetic tree of Que_HT-5, the paralogs of the horizontally transferred TEs in the three genera *Camellia*, *Quercus*, and *Vitis* were nested within the homologs of *Juglans*, indicating that *Juglans* was the donor for the three genera (Figure 3b, red branches in the phylogenetic tree). The activity history of the homologs of the horizontally transferred TE in *Juglans* appeared under the 90% identity threshold, whereas those of the rest did not (Figure 3b, upper panel). Therefore, in this case, *Vitis* was classified as the recipient, along with *Camellia* and *Quercus*. The phylogenetic analysis of Pha_HT-1 revealed that the homologs of the horizontally transferred TEs in the seven genera were nested within the *Vitis* homologs (Figure 3c, red branches in the phylogenetic tree). The activity history of the homologs of the four *Vitis* species appeared under the 90% identity threshold, while that of the homologs of the seven genera did not. In this case, *Vitis* was considered the donor (Figure 3c), and the other seven genera were considered the recipients.

### 2.3. Donors and Recipients of the HT Events

**Figure 4:**
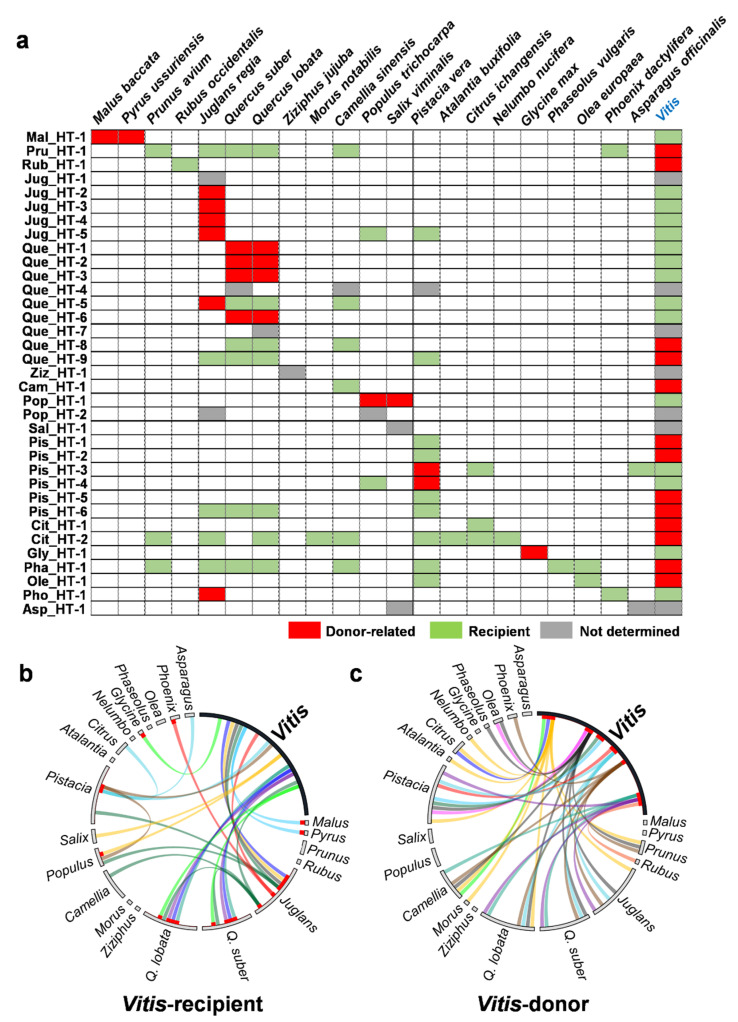
Donor and recipient of the 35 HT events. (**a**) The donor, recipient, and undetermined species are presented with red, green, and gray boxes, respectively. The columns and rows indicate the species and each HT event, respectively. The name of the species and *Vitis* genus is shown at the top of the panel. The name of the HT event is shown on the left side of the panel. (**b**,**c**) Circos plots representing HT events when *Vitis* was the recipient (**b**) or donor (**c**), respectively. The bars that comprise the circles represent the species or genus involved in the HT events, and the length of the bar represents the number of HT events in each species or genus. For each HT event, the donor is marked with a red box on the inner side of the circle bars. The lines in the circle link the donor and recipient species.

The donor and recipient could be inferred for 28 of the 35 HT events by activity history and phylogenetic analyses (Figure 4a, Supplementary Figure S4). Interestingly, 12 HT events had multiple recipients with the same element (Figure 4a; Pru_HT-1, Jug_HT-5, Que_HT-5, Que_HT-8, Que_HT-9, Pis_HT-3, Pis_HT-4, Pis_HT-6, Cit_HT-2, Pha_HT-1, Ole_HT-1, and Pho_HT-1). Because most HT events showed an approximately 91% identity (Figure 2a), they were expected to occur before the speciation of the species involved. Therefore, the donor and recipient species reveal not the direct species but the progeny or relatives of the species involved in the HT events. The number of HT events with *Vitis* species as recipients was 15, and the remaining 13 HT events included *Vitis* species as donors. Among the non-*Vitis* genera, *Juglans* was the most frequent donor to *Vitis*, donating six HT events (Figure 4b). When *Vitis* species were the donors, *Pistacia* was the most frequent recipient (in nine out of 13 events for which *Vitis* was the donor; Figure 4c). In contrast, only two TEs were transferred from *Pistacia* to *Vitis* (Figure 4a, Pis_HT-3 and Pis_HT-4).

In the case of Mal_HT-1 and Pop_HT-1, two species were involved as donors in each event, and they belonged to the same family, Rosaceae (*M. baccata* and *P. ussuriensis*) and Salicaceae (*Populus trichocarpa* and *Salix viminalis*), respectively (Figure 2b). In Mal_HT-1, the species distance between *M. baccata* and *P. ussuriensis* was 93.6% (Supplementary Figure S3), but the identity of the horizontally transferred TE between *M. baccata* and *V. vinifera* was 91.9%, and that between *P. ussuriensis* and *V. vinifera* was 91.3% (Supplementary Figure S1). This indicates that the HT event occurred before the speciation of the two species. Therefore, these two Rosaceae species were considered donors (Figure 4). In the case of Pop_HT-1, the species distance between *P. trichocarpa* and *S. viminalis* was 89.9% (Supplementary Figure S3). However, the identity of the horizontally transferred TE was higher than that as 95–97% among *Vitis, P. trichocarpa* and *S. viminalis* (Supplementary Figure S1). This may indicate that there was HT or introgression of the TE between *P. trichocarpa* and *S. viminalis* after speciation, and the same element was horizontally transferred to *Vitis*. Therefore, these two Salicaceae species were also considered donors (Figure 4).

### 2.4. Horizontally Transferred LTR Retrotransposons in the Vitis Genomes

**Figure 5:**
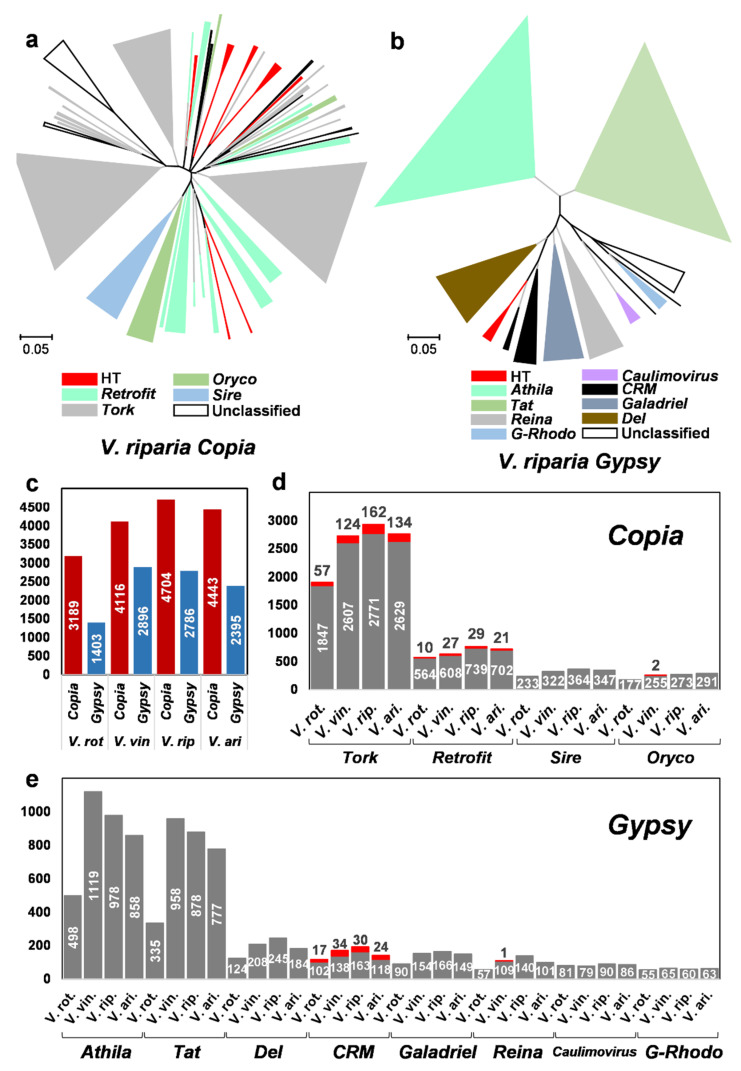
Composition of the horizontally transferred LTR retrotransposons in the four *Vitis* genomes. (**a**,**b**) Phylogenetic trees of *Copia* (**a**) and *Gypsy* (**b**) elements in *V. riparia*. The clusters are represented with colored triangles, and the classified families are presented in different colors. The unclassified clusters are presented with empty triangles. The clusters for the horizontally transferred LTR retrotransposons are marked with red triangles. (**c**) The numbers of RTs in the four *Vitis* genomes are compared. The sizes of the *Copia* and *Gypsy* superfamilies are shown by red and blue bars, respectively, and the values are shown in each of the bars. (**d**,**e**) The sizes of *Copia* (**d**) and *Gypsy* (**e**) families and the proportions of horizontally transferred elements in each family are compared. The number of LTR retrotransposons (excluding horizontally transferred elements) and the horizontally transferred elements are presented with gray and red bars, respectively, and the values are shown in the middle or at the top of the bars. The species name and the name of the LTR retrotransposon family are shown at the bottom of the panel. Abbreviations: *V. rot.*: *Vitis rotundifolia*, *V. vin.*: *Vitis vinifera*, *V. rip.*: *Vitis riparia*, and *V. ari*.: *Vitis arizonica*.

Because 34 out of the 35 HT events in the *Vitis* genomes involved LTR retrotransposons, we investigated the content of horizontally transferred LTR retrotransposons in the four *Vitis* genomes. For this, we isolated the reverse transcriptase (RT) domain sequences of the two LTR retrotransposon superfamilies *Copia* and *Gypsy* from the four *Vitis* genomes. The *Copia* and *Gypsy* elements were classified into families according to the GyDB RT database (GyDB, https://gydb.org/, accessed on 25 September 2021), and phylogenetic trees were inferred with the classified RT sequences (Figure 5a,b and Supplementary Figure S5).

Among the four *Vitis* species, the total number of RT sequences was highest in *V. riparia,* with 4704 *Copia* and 2786 *Gypsy* elements (Figure 5c). In all four species, the number of *Copia* elements was 1.4–2.3× higher than that of *Gypsy* elements. The consequence of HTs for the composition of LTR retrotransposons was investigated, focusing on the 15 HT events for which *Vitis* species were the recipients (Figure 5a,b). According to the phylogenetic tree of *V. riparia*, a total of seven clusters in *Copia* and one in *Gypsy* were newly formed by HTs. The number of clusters newly formed by HTs in the other three species was 7–8 in *Copia* and 1–2 in *Gypsy* (Supplementary Figure S5).

According to the number of RTs, the proportion of LTR retrotransposons obtained by HT in the four *Vitis* genomes was 2.1–4.1% for *Copia* and 1.0–1.2% for *Gypsy*. A total of four families were identified from the *Copia* superfamily in the *Vitis* genomes. Among them, *Tork* was the largest family, and 3.0–5.5% of the family members were newly generated by paralogs of the horizontally transferred LTR retrotransposons (Figure 5d). In *Retrofit*, 1.7–4.3% of the new family members were generated by HTs. In the *Gypsy* superfamily, a total of eight families were identified, but only *CRM* and *Reina* contained horizontally transferred elements (Figure 5e). Interestingly, *CRM* contained the highest proportion of paralogs of the horizontally transferred LTR retrotransposons, at 14.3–19.8%, in the four *Vitis* species. In the case of *Reina*, only one horizontally transferred LTR retrotransposon was identified in *V. vinifera*.

**Figure 6:**
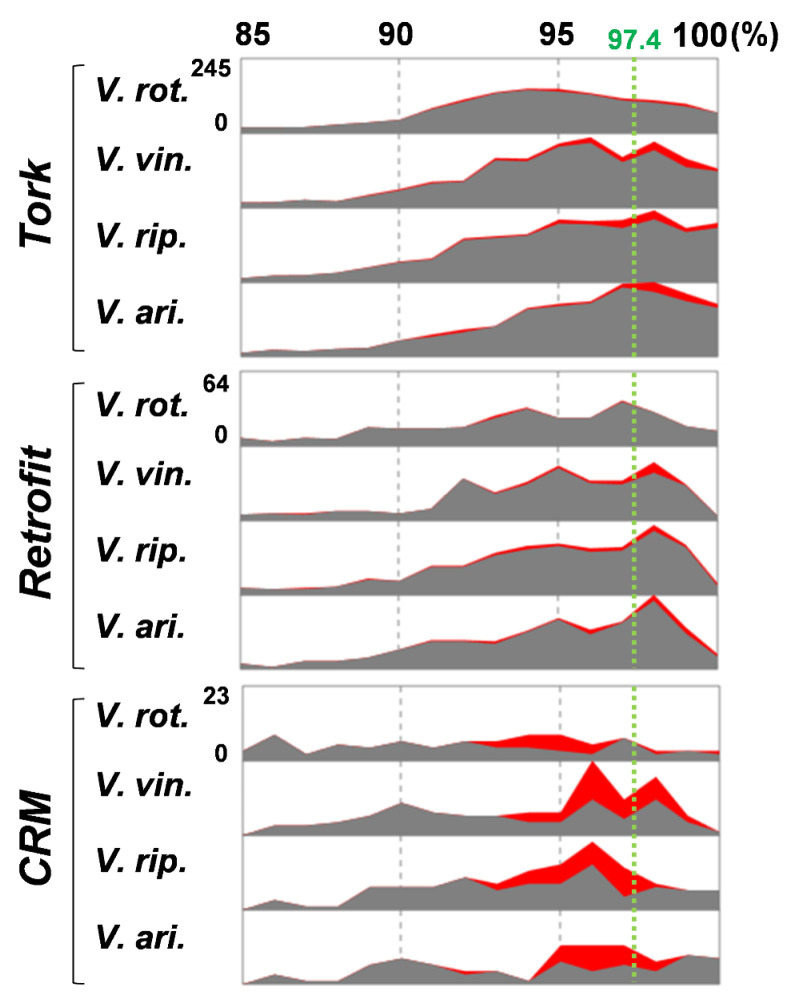
Activity history of LTR retrotransposon families involved in HT. The relative activity history of the three LTR retrotransposon families, *Tork*, *Retrofit*, and *CRM*, is presented. The activity history for the native LTR retrotransposons is shown with gray graphs, and that for paralogs of the horizontally transferred elements is shown with red graphs. The range of the X-axis is shown at the top of the panel. The estimated speciation point of *V. rotundifolia* is indicated with green dotted lines, and its identity value is shown at the top of the line. The range of the Y-axis is shown on the left side of each panel, and the range is the same as that of the other three *Vitis* species. The abbreviations are the same as in Figure 5.

To investigate how horizontally transferred LTR retrotransposons contribute to the genome diversity of *Vitis*, the activity history of the native elements and the paralogs of the horizontally transferred LTR retrotransposons were analyzed in *Tork*, *Retrofit*, and *CRM* (Figure 6). *Tork* was the largest LTR retrotransposon family in the four *Vitis* genomes, with 1904–2933 family members, but *Retrofit* and *CRM* were relatively small families, with 574–768 and 119–193 members, respectively. The pairwise distance between *V. rotundifolia* and the remaining three *Vitis* species was ~97.4% (Figure 6, green dotted lines), indicating that speciation occurred under approximately this identity. The activity peak of each of the LTR retrotransposon families in each species was determined to be the highest value in the activity histogram. In *Tork*, the activity peak in *V. rotundifolia* appeared at approximately 94% and gradually decreased thereafter. In contrast, the peak in the other three species appeared at approximately 96% or 98%. The activity history of the homologs of the horizontally transferred elements also appeared to differ between *V. rotundifolia* and the other three *Vitis* species. While the low-level activity of the homologs was observed in *V. rotundifolia*, the other three species showed an increase in activity around the speciation point of *V. rotundifolia*. Similar activity patterns of the homologs of the horizontally transferred elements were observed in *Retrofit* and *CRM*. In the case of *CRM*, the native elements showed steady activity, and the burst activity of the horizontally transferred elements caused an activity peak at an approximately 96% identity in *V. vinifera*, *V. riparia*, and *V. arizonica*. These results demonstrate that horizontally transferred LTR retrotransposons can settle in host genomes and contribute to the genome diversity of host species through their differential activity.

## 3. Discussion

### 3.1. Detection of HTs

To detect HTs, three types of inferences have been commonly used: (1) sequence similarity, (2) phylogenetic incongruences, (3) patchy distribution [30]. In this study, we used the sequence similarity and phylogenetic incongruences along with the activity history of the horizontally transferred TEs. The expected sequence similarity without HTs was estimated by aligning the 3rd codons of orthologous gene sets between *Vitis* species and the 20 plant genera that showed HTs. The box plots of the 3rd codon identity showed the median values between 52–65% (Figure 1a). To detect HTs, we used the 90% identity threshold. In a similar previous study by El Baidouri et al., a 90% threshold was used within monocot or dicot classes and 85% between the two classes to detect horizontally transferred LTR retrotransposons [20]. Therefore, the 90% threshold used in this study for all classes is high enough to detect HTs. The phylogenetic analyses with the homologs of each of the 35 horizontally transferred TEs revealed phylogenetic incongruences by showing that a cluster of homologs of a species was nested among the homologs of other species or branches of homologs of different species were intermixed (Figure 3 and Supplementary Figure S4). In particular, the 28 HT events that determined donor and recipient species revealed that the activity history of the homologs corresponded to the expected donor and recipients from the phylogenetic tree by showing the activity of donor homologs under the 90% threshold and that of recipients over it. These facts demonstrate that the HT events identified in this study are true.

In the previous study by El Baidouri et al., HTs of LTR retrotransposons in *Vitis* were identified with four other plant families, Arecaceae, Salicaceae, Rosaceae, and Rutaceae [20]. In this study, we identified a total of 15 plant families that reveal HT events with *Vitis* species, including the four previously known families. Of the 35 HT events in this study, two events with Rutaceae, one with Salicaceae, and one with Rosaceae corresponded to the HT events identified in the previous study (Supplementary Figure S6). 

### 3.2. HT of LTR Retrotransposons and Diversification of the Vitis Genomes

Our study revealed that horizontally transferred LTR retrotransposons contribute to genome diversification in the four *Vitis* species. The identified proportion of horizontally transferred LTR retrotransposons, 2.1–4.1% for *Copia* and 1.0–1.2% for *Gypsy*, was the minimum quantity identified by our HT screening method. The horizontally transferred LTR retrotransposons created 8–10 new clusters (or branches) in the four *Vitis* genomes (Figure 5 and Supplementary Figure S5). In the *Vitis* genomes, *Copia* was the major LTR retrotransposon superfamily, and 82.9% of the horizontally transferred TEs were identified as *Copia* elements. Considering that the proportion of *Copia* elements in the four *Vitis* genomes was 58.7–69.4%, the identified HT events were biased toward *Copia* elements.

Whereas *Gypsy* elements are prone to appear in heterochromatic regions in many plant species [5,31,32], *Copia* elements are easily found in gene-rich regions because of their random insertion pattern [33,34]. *Copia* elements are known to respond to diverse biotic or abiotic stresses such as pathogens, compounds related to plant defense, wounding, and freezing [9,35,36,37,38]. These facts suggest that horizontally transferred *Copia* elements played a role in the evolution of *Vitis* species by providing broader options to respond to environmental stress through their activity.

The activity history of the horizontally transferred LTR retrotransposons also revealed how they contribute to host genome diversification. In our analysis, the major difference in LTR retrotransposon content in the *Vitis* genomes appeared in the native LTR retrotransposon families, such as *Tork*, *Athila*, and *Tat* (Figure 5d,e and Figure 6, and Supplementary Figure S7). In particular, the activity peaks of *Tork, Athila, Tat,* and *Retrofit* appeared around the speciation point of *V. rotundifolia*, but the peaks appeared differently between *V. rotundifolia* and the remaining three *Vitis* species (Figure 6 and Supplementary Figure S7). In addition to the native elements, the horizontally transferred LTR retrotransposons showed differential activity between *V. rotundifolia* and the other three *Vitis* species. While the activity of the horizontally transferred *Tork* and *Retrofit* elements was lower in *V. rotundifolia*, it was increased around the speciation point in the remaining three species (Figure 6). The *CRM* family displayed apparent differences in horizontally transferred elements. The activity peak of the *CRM* family was formed by the active proliferation of horizontally transferred elements in *V. vinifera*, *V. riparia*, and *V. arizonica* but not in *V. rotundifolia*. (Figure 6). These results demonstrate that the differential activity of the horizontally transferred LTR retrotransposons partially contributes to the diversification of the *Vitis* genomes.

### 3.3. Multiple Recipients of Horizontally Transferred Elements

Among the 35 HT events, 12 events involved multiple recipients of the same element. In our study, nine out of the 22 genera included donor species. Among the nine genera, only three genera, *Juglans*, *Pistacia*, and *Vitis*, were donor species to multiple recipients. The multiple recipients might be related to the burst of HT events that occurred at a sequence identity of approximately 91% (Figure 2a). The species distance between *Populus* and *Salix* was consistent with the timing of the burst at 89.9% (Supplementary Figure S3). The speciation date of *Populus* and *Salix* was calculated as ~48 million years ago on the basis of fossil evidence, placing it in the early middle Eocene [39]. The middle Eocene to early Oligocene included drastic global climate changes from the warmest to an icehouse climate, leading to global extinctions of species [40].

Considering the extreme climate conditions when the burst of HT occurred, a possible hypothesis for the multiple recipients would be as follows. During this severe climate change, the *Copia* elements that responded to environmental stress might have been highly activated, which would have facilitated the adaptation of species by creating genetic diversity such as changes in gene expression, gene mutation, and gene inactivation [41]. If the genetic diversity created by the activated *Copia* elements was favorable to the adaptation of the species, the elements would become fixed in the population. The findings of our previous study suggested that the HT of TEs might occur via an intermediate for the proliferation of active TEs [22]. Accordingly, the highly activated *Copia* elements would provide higher chances of HT, leading to frequent HT events. The fact that 88.2% of the horizontally transferred LTR retrotransposons were *Copia* elements (Figure 1b) supports this speculation. In addition, 11 out of the 12 HT events (91.7%) that involved multiple recipients also involved *Copia* elements (Figure 4). Therefore, the donor species participating in frequent HT events with stress-sensitive *Copia* elements would have had a higher chance of having multiple recipients.

The phylogenetic analysis of the HT events that revealed multiple recipients showed a mixed cluster or mixed branches of the paralogs of the horizontally transferred elements among the multiple recipients and the homologs of the host species (Supplementary Figure S4, Cit_HT-2, Jug_HT-5, Pha_HT-1, Pis_HT-6, Pru_HT-1, and Que_HT-9). This may indicate that the HTs between the donor and multiple recipients occurred at a similar time point, corresponding to the burst of HTs at an identity of approximately 91% (Figure 2a), and the similar starting point of the divergence of the horizontally transferred TEs caused the mixed branches of the TE homologs.

In our previous study of HT among grass species, we suggested insect-mediated HT as a possible mechanism [22]. Three conditions are required to explain cases of multiple recipients with single elements by vector-mediated HT. First, the vector should have a broad range of hosts to promote physical contact with the donor and multiple recipients. Second, HT should occur from the donor to the vector genome first. Third, HT should occur from the vector to multiple recipients with the same elements. *Phellinus*, a genus of fungi, has a broad range of plant hosts, and 13 out of the 15 families (except for Nelumbonaceae and Oleaceae) with HTs in this study were identified as its hosts [42]. Therefore, *Phellinus* is a candidate vector that satisfies the first condition. A parasite plant would also be a possible vector. *Saparia*, an endoparasitic flowering plant, showed host-to-parasite horizontal gene transfers with Vitaceae [43], which satisfies the second condition. To understand the mechanisms of HTs to multiple recipients, further studies on interkingdom or host-parasite HT with these organisms are needed.

### 3.4. Conclusions

Although the HT of TEs is suggested to be a major force for variation and biological innovation in eukaryotic genomes [23], related studies in plant genomes were limited to proving the existence of horizontally transferred TEs [20,22,24,25]. This study provides the first evidence that the HT of TEs contributes to the diversification of plant genomes through the differential activity of horizontally transferred LTR retrotransposons. In addition, our study revealed HTs between Vitaceae and a wide range of plant families, including herbaceous and woody plants. These may indicate that the HT of TEs is not a rare or unique phenomenon for certain species but one of the general life mechanisms for plant evolution through genome diversification.

## 4. Materials and Methods

### 4.1. Screening of Possible HT Cases

To screen possible HT cases, we used the genome sequences of four *Vitis* and 137 other plant species available from the public genome database (Supplementary Table S1). The four *Vitis* whole-genome sequences were merged into a single file and were used as a query sequence in the BLAST search against each of the 137 whole-genome sequences from other plants (identity: >90%, e-value: <e^−10^, score: >500, match length: >500 bp). The sequences of the BLAST hits were isolated from the non-*Vitis* genomes. Among the BLAST hits, organelle genome sequences, ribosomal sequences, or highly conserved genes were filtered out. After filtering, the remaining sequences were considered candidate sequences involved in HTs. To remove redundant copies of the candidate sequences, all-to-all BLAST with all the candidate sequences was performed (single high-scoring segment pair (HSP), identity: >90%, e-value: <e^−10^, score: >500), and the sequence clusters that had a >90% identity among the cluster members were identified. The sequences in these clusters were considered redundant copies, and only one was kept. The sequences that passed the three filtering steps, including adherence to a 90% identity threshold, conserved sequence status, and redundant copy removal, were considered the DNA sequences for putative HT events. The horizontally transferred DNA sequences for the 35 putative HT events were used to identify other HTs between the four *Vitis* and other 21 plant species for each event. For this, all the horizontally transferred sequences of the 35 HT events were compared to the genomes of the 25 plants involved in the events with a BLAST search (single HSP, identity: >90%, e-value: <e^−10^, score: >500). Among the results, the best hits for each species with a >90% identity and >500 bp match length were considered putative HTs with additional species.

The genetic distances among the species involved in the putative HT events were calculated by the identity of the 3rd codon of the orthologous coding sequence (CDS) sets between *V. vinifera* and the other 20 species. In the case of the *Quercus* genus, the coding sequences of *Q. lobata* were used. The orthologous CDS sets were identified with all the CDSs of *V. vinifera* and each of the 20 species by reciprocal best hits of BLAST analyses (single HSP, identity: >60%, e-value: <e^−5^, score: >200). The matched sequences of *V. vinifera* and the compared species were isolated and translated into amino acids with different codon frames. The codon frames with zero stop codons were identified in both sequences. The in-frame codon sequences were compared again using codon alignment with PRANK v.170427. After alignment, the gaps were removed, and the pairwise identity of the 3rd codon was calculated.

### 4.2. Activity History of TE Paralogs and Phylogenetic Analysis of TE Homologs

The putative horizontally transferred DNA sequences were annotated and classified with the Repbase database (giri REPBASE, https://www.girinst.org/, accessed on 25 September 2021). The validation of HTs was performed by analyzing the relative activity history and phylogenetic relations of the homologs of the horizontally transferred TEs.

The relative activity history of TEs was inferred with the UPGMA algorithm [44] to use the pairwise identity distances between paralogous sequences of TEs. To avoid split alignments by BLAST, we used small (260–280 bp) sequence fragments in the analysis. The sequence fragments were isolated from the reverse transcriptase (RT) domain if it existed or otherwise from other arbitrary positions of the DNA sequences involved in the 35 HT events. The sequence fragments were used to isolate paralogous copies from each of the plant genome sequences involved in the HT event. Candidate paralogous copies were identified with BLAST analysis (single HSP, score > 60, and e-value: <e^−8^). To avoid mixing with false paralogs from other TE families, the candidate paralogous copies were compared by all-to-all BLAST in each species (single HSP, score > 60, and e-value: <e^−8^), and the sequence clusters that showed >85% identity with each other were considered true paralogous copies. The pairwise distance of the paralogous copies in each species was calculated by all-to-all BLAST analysis (single HSP, score > 60, and e-value: <e^−8^). After removing the hits caused by self-matches, a UPGMA phylogenetic tree was inferred by clustering the sequences from the highest to lowest identity. The values at all nodes of the tree were calculated by the UPGMA algorithm. All the values were considered divergence points of TE paralogs and used to draw histograms of the relative activity history of the TEs. An in-house Python script was used to calculate the node values of the UPGMA tree, and software used in the previous study by Park et al. [22] was employed. The phylogenetic tree was inferred from all the homologs of the horizontally transferred TEs included in the activity history analyses. All the homologous sequences were aligned with MUSCLE as implemented in MEGA 7.0 software [45], and the phylogenetic tree was constructed by the neighbor-joining method (p-distance and pairwise deletion). The donor and recipient species were determined with the phylogenetic analysis and activity history data. If the donor and recipient could not be determined, only the putative HT events that showed mixed branches or mixed clusters among the involved species in the phylogenetic analysis were considered HT events to avoid possible false positives.

### 4.3. Phylogenetic Tree of the Species Involved in the HT Events

To construct a phylogenetic tree of the 25 species involved in the HT events, we used orthologous coding sequences of all 25 species. The orthologous coding sequences were isolated from repeated isolation of reciprocal best hits by BLAST analyses (single HSP, identity: >60%, e-value: <e^−5^, score: >200) using nuclear genes of the 25 species. A total of 582 orthologous coding sequence sets were identified, and 172 of them were randomly selected for the subsequent analysis. Each of the 172 orthologous coding sequence sets was aligned based on codons using MUSCLE as implemented in MEGA 7.0, and the unaligned regions were manually trimmed. All 172 aligned orthologous sequences were concatenated into a single sequence in each species, and the 3rd codons were used to calculate pairwise distances and construction of the phylogenetic tree. To compare the 90% identity threshold for HT investigation with the distances among the 25 species, we used the UPGMA phylogenetic tree, which revealed pairwise distances based on sequence identity. The phylogenetic tree was constructed with MEGA 7.0 (UPGMA algorithm, bootstraps: 100, p-distance, and complete deletion).

### 4.4. Analysis of LTR Retrotransposons in the Four Vitis Genomes

To analyze the LTR retrotransposons in the *Vitis* genomes, RT sequences were isolated using HMMER v3.3.2. The hmm profiles for HMMER analyses were generated using the aligned RT sequences of *Copia* and *Gypsy* elements obtained from GyDB (GyDB, https://gydb.org/, accessed on 25 September 2021). The aligned RT sequences were trimmed to a 92 amino acid (aa) sequence length, and these were used to generate the hmm profile. The RT sequences of the four *Vitis* species were isolated from the HMMER results (e-value: >e^−10^ and aa length: >50). The RT sequences of the *Gypsy* and *Copia* elements were classified into families by BLAST search (BLASTp, best hit, single HSP, score: >50, and e-value: >e^−10^) with the RT sequences obtained from GyDB. The nucleotide sequences of the classified RT aa sequences were retrieved, and the homologs of the horizontally transferred LTR retrotransposons were identified with BLAST analysis (single HSP, score: >200, and e-value: >e^−10^). The phylogenetic trees were constructed with all the RT sequences of *Copia* and *Gypsy* by the neighbor-joining method as implemented in MEGA 7.0 (p-distance and partial deletion with an 80% site coverage cutoff). The activity history of the LTR retrotransposon families was analyzed using the same method used for the activity history of TE paralogs.

## Supplementary Materials

The following are available online at https://www.mdpi.com/article/10.3390/ijms221910446/s1.
