# Supplementary material for: Horizontal Transfer of LTR Retrotransposons Contributes to the Genome Diversity of Vitis
Source: Int J Mol Sci. 2021 Sep 28;22(19):10446. doi: 10.3390/ijms221910446 (PMC8508631; doi:10.3390/ijms221910446)
Supplement: Supplementary file 1 [file ijms-22-10446-s001.zip › Supplementary_Figures.pdf]

Supplementary Figure S1

	Malus baccata	*Pyrus ussuriensis*	*Prunus avium*	*Rubus occidentalis*	*Juglans regia*	*Quercus suber*	*Quercus lobata*	*Ziziphus jujuba*	*Morus notabilis*	*Camellia sinensis*	*Populus trichocarpa*	*Salix viminalis*	*Pistacia vera*	*Atalantia buxifolia*	*Citrus ichangensis*	*Nelumbo nucifera*	*Glycine max*	*Phaseolus vulgaris*	*Olea europaea*	*Phoenix dactylifera*	*Asparagus officinalis*	*Vitis rotundifolia*	*Vitis vinifera*	*Vitis riparia*	*Vitis arizonica*
Mal_HT-1	1	0.974																					0.919		
Pru_HT-1			1		0.92	0.938	0.931			0.92										0.916		0.912	0.922		
Rub_HT-1				1																		0.906	0.910	0.909	0.913
Jug_HT-1					1																	0.931	0.925	0.923	0.930
Jug_HT-2					1																	0.907	0.910	0.910	0.909
Jug_HT-3					1																	0.902	0.909	0.905	0.904
Jug_HT-4					1																		0.912	0.917	0.916
Jug_HT-5					1						0.913		0.904									0.912	0.903	0.909	
Que_HT-1						0.926	1																0.977	0.973	0.977
Que_HT-2						0.937	1															0.919	0.919	0.922	0.932
Que_HT-3						1	0.944																0.918	0.916	0.920
Que_HT-4							1			0.91			0.912									0.904	0.904	0.901	0.903
Que_HT-5					0.920	1	0.947			0.92												0.906	0.900		0.906
Que_HT-6						0.930	1															0.952	0.955	0.956	0.956
Que_HT-7						1																0.922	0.920	0.920	0.924
Que_HT-8						1	0.915			0.93													0.911	0.906	0.909
Que_HT-9					0.93	0.952	1						0.910									0.923	0.921	0.922	0.924
Ziz_HT-1								1														0.951	0.953	0.947	0.954
Cam_HT-1										1												0.910	0.914	0.909	0.906
Pop_HT-1											1	0.950												0.963	0.969
Pop_HT-2					0.91						1												0.911	0.913	0.913
Sal_HT-1												1												0.901	0.900
Pis_HT-1													1										0.909		
Pis_HT-2													1									0.907		0.905	0.906
Pis_HT-3													1		0.930						0.93		0.931		
Pis_HT-4											0.913		1										0.930		
Pis_HT-5													1									0.904	0.903	0.905	0.908
Pis_HT-6					0.93	0.923	0.925						1									0.926	0.925	0.922	0.922
Cit_HT-1															1							0.952	0.947	0.955	0.953
Cit_HT-2			0.909		0.91	0.919			0.92	0.91			0.907	0.91	1	0.922						0.916	0.903	0.903	
Gly_HT-1																	1						0.904		
Pha_HT-1			0.901		0.93	0.922	0.913			0.92			0.918					1	0.914			0.913	0.917	0.924	0.913
Ole_HT-1													0.905						1			0.911	0.910	0.906	0.916
Pho_HT-1					0.92															1		0.900			
Asp_HT-1												0.904									1	0.904	0.907	0.903	0.905

Supplementary Figure S1. Sequence identity of the horizontally transferred TEs. Columns and rows represent species and each of the TE events, respectively. The intensity of the red color in each box reveals identity. The boxes with 1.0 identity appear when the used sequence matches itself.

Supplementary Figure S2

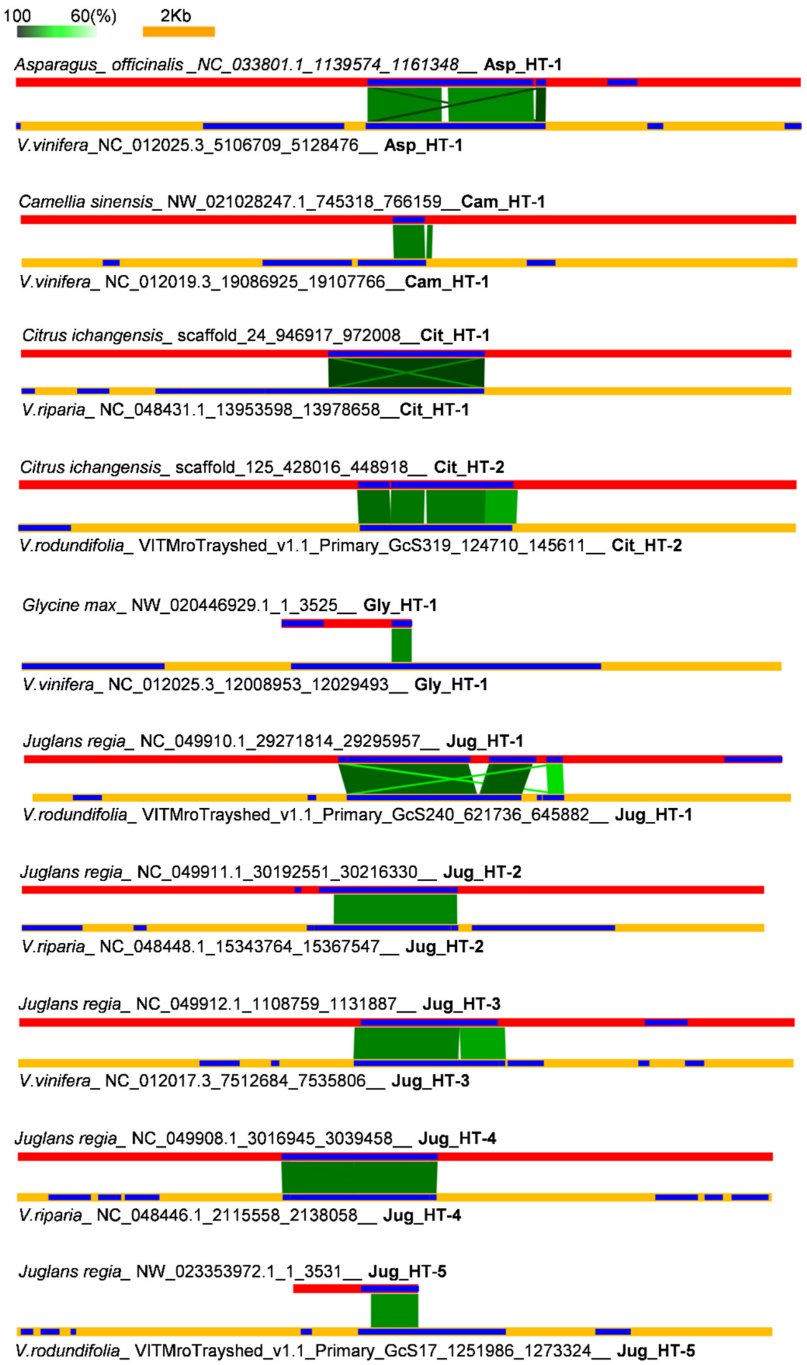

Supplementary Figure S2. *Cont.*

Supplementary Figure S2

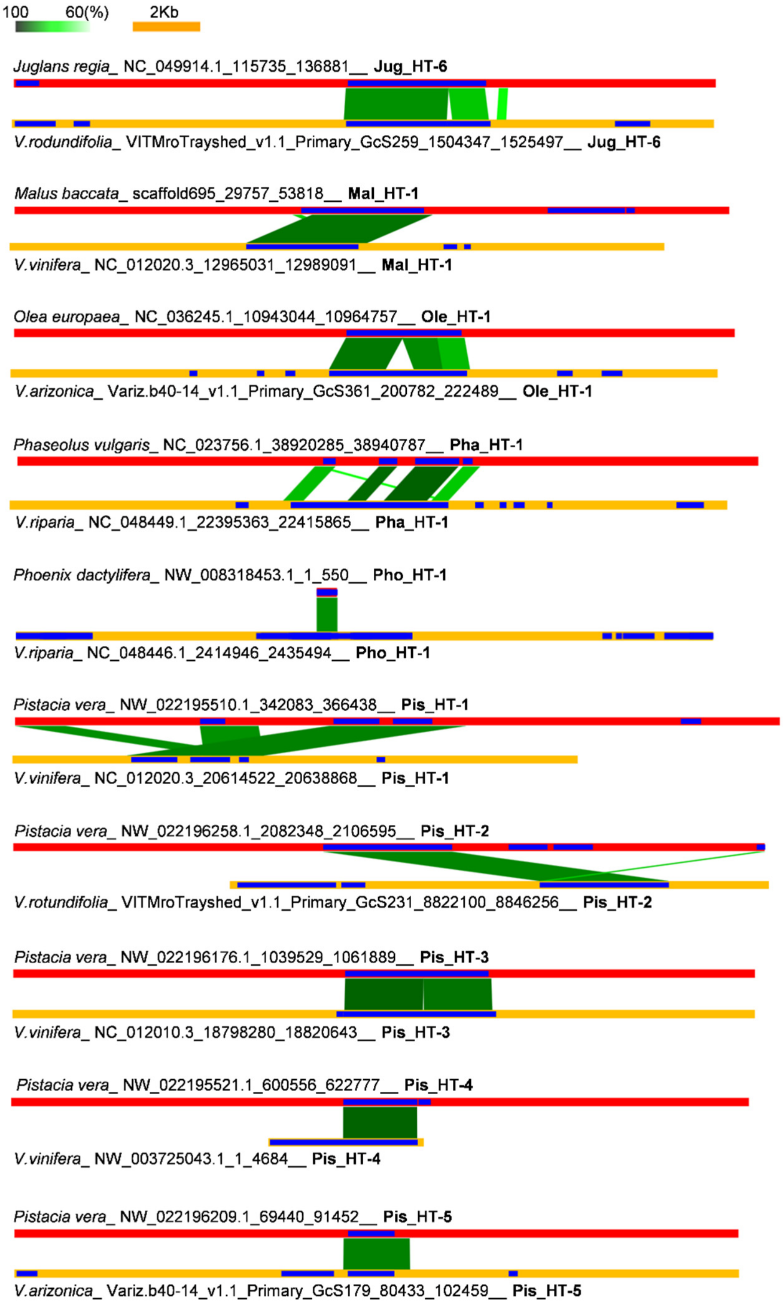

Supplementary Figure S2. *Cont.*

Supplementary Figure S2

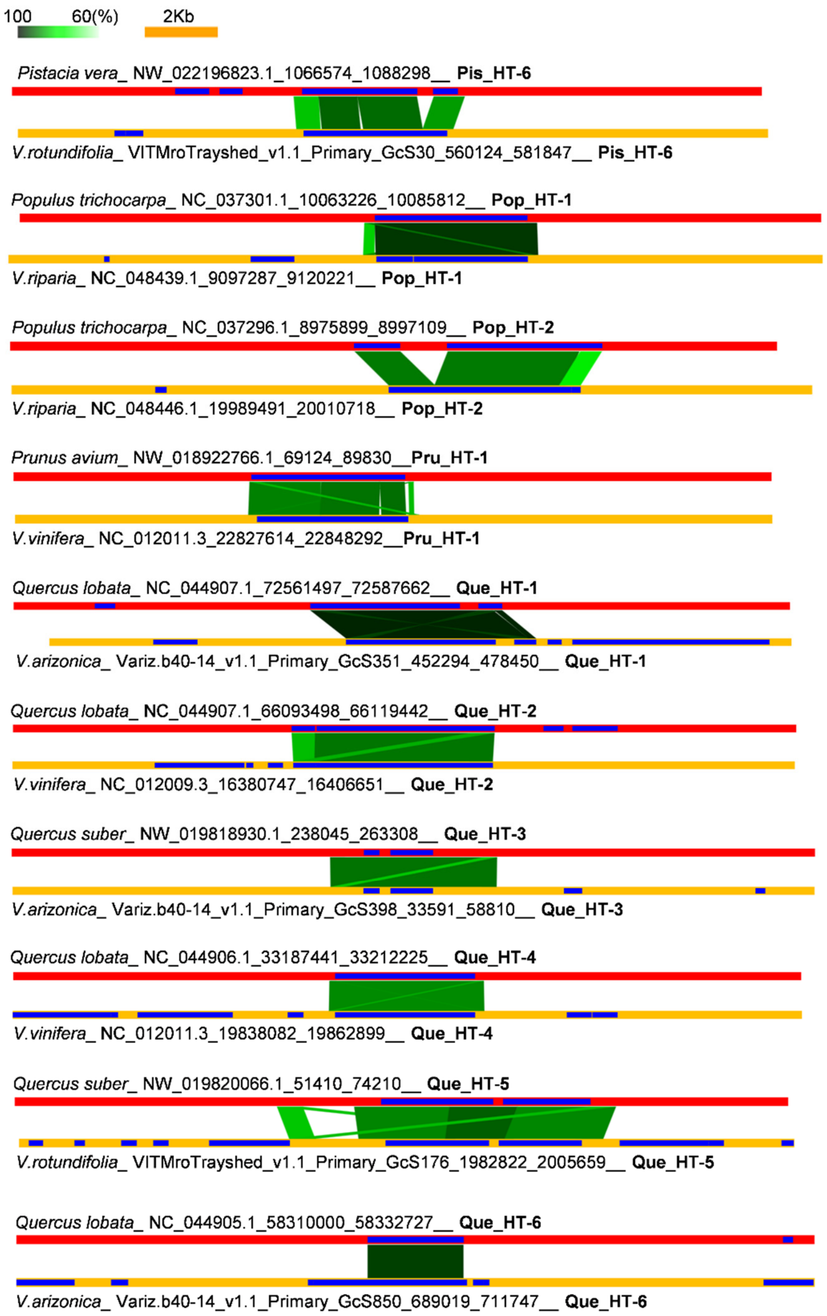

Supplementary Figure S2. *Cont.*

Supplementary Figure S2

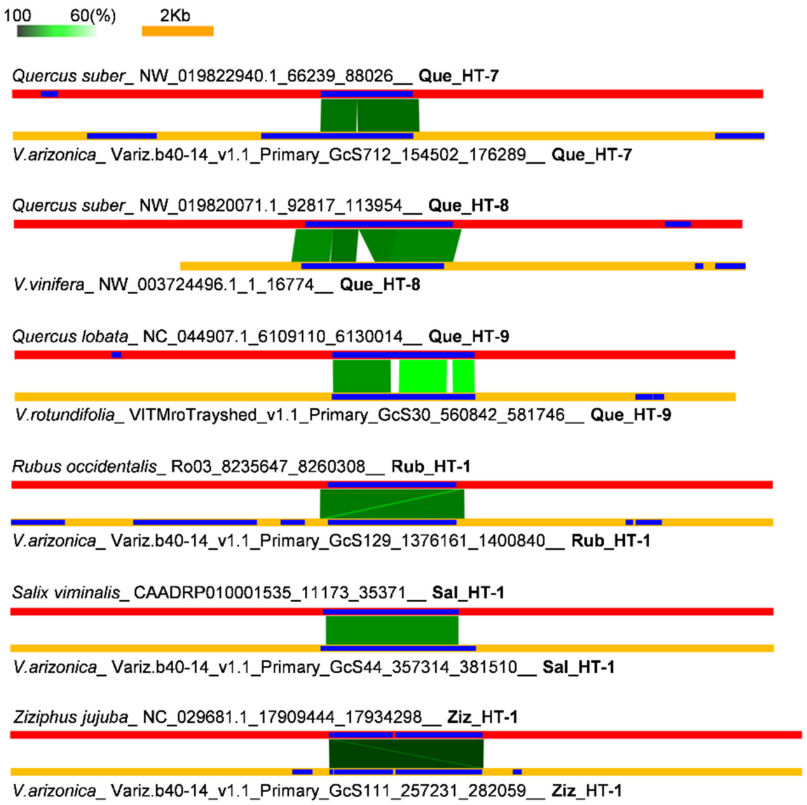

Supplementary Figure S2. Comparative sequence analysis of the 35 HT events. The sequence contigs containing the horizontally transferred TEs are compared to the *Vitis* collinear regions. The horizontal red and yellow bars represent non-*Vitis* and *Vitis* sequence contigs, respectively. The blue bars indicate annotated TEs. The matched sequence regions are linked with green boxes and the color intensity is proportional to the identity.

Supplementary Figure S3

	Malus	*Pyrus*	*Prunus*	*Rubus*	*Juglans*	*Q. suber*	*Q. lobata*	*Ziziphus*	*Morus*	*Camellia*	*V. Rotundifolia*	*V. vinifera*	*V. riparia*	*V. arizonica*	*Populus*	*Salix*	*Pistacia*	*Atalantia*	*Citrus*	*Nelumbo*	*Glycine*	*Phaseolus*	*Olea*	*Phoenix*
Pyrus	93.6																							
Prunus	82.3	83.1																						
Rubus	75.3	75.3	78.4																					
Juglans	65.8	65.4	66.9	65.6																				
Q. suber	66.7	66.8	68.7	67.2	77.6																			
Q. lobata	66.7	66.8	68.9	67.2	77.8	97.2																		
Ziziphus	66.9	66.9	68.7	68	66.1	67.7	67.7																	
Morus	65.5	65.1	66.9	65.1	64.1	65	65	69																
Camellia	61	60.7	62.1	61.5	63.5	64.5	64.5	61.8	59.8															
V. Rotundifolia	62.5	62.4	64.2	63.5	65.5	67	67.2	64.1	61.6	66.3														
V. vinifera	62.5	62.3	64.1	63.5	65.4	67	67.1	64	61.4	66.2	97.3													
V. riparia	62.5	62.3	64.2	63.5	65.4	66.9	67.1	64.1	61.4	66.2	97.3	98.7												
V. arizonica	62.5	62.3	64.2	63.5	65.4	67	67.1	64	61.5	66.2	97.5	98.8	99.1											
Populus	62	62.2	63.8	62.5	64	65.6	65.6	63	60.6	61.2	63.3	63.3	63.3	63.3										
Salix	61.4	61.4	63	61.9	63.4	64.7	64.8	62.4	60	60.7	62.6	62.6	62.6	62.6	89.9									
Pistacia	63	62.9	64.5	63.6	65.2	67	67.1	64.1	61.4	62.2	64.9	64.7	64.8	64.8	65.4	64.4								
Atalantia	61.1	61.2	62.9	62.2	63.9	65.5	65.6	62.8	60.1	61	63.1	63.1	63	63.1	63.6	62.8	73.2							
Citrus	61.6	61.5	63	62.3	64.5	65.8	65.9	63	60.6	61.3	63.2	63.1	63.1	63.2	63.8	63	73.5	94						
Nelumbo	57.7	57.7	58.8	58.2	59.8	60.4	60.4	58.8	57	60.4	62.3	62.4	62.4	62.4	58.1	57.5	58.7	57.8	57.9					
Glycine	59.6	59.3	60.4	59.8	61.9	63	63.1	60.4	58.5	57.9	59.6	59.6	59.6	59.6	59	58.4	60	58.7	59.1	55.7				
Phaseolus	57.8	58	59.1	58.5	60.2	61.4	61.6	59.3	56.9	56.8	58.3	58.3	58.3	58.3	57.7	57.1	58.7	58	57.5	54.8	82.2			
Olea	54.4	54.6	55.5	55.1	56.4	57.3	57.5	55.5	54	60.2	58.6	58.6	58.6	58.6	55.6	55	56.2	55.5	55.2	55.4	53.3	53.2		
Phoenix	52.6	52.5	53	52.7	53.2	53.8	53.8	52.5	51.5	53.5	54.5	54.5	54.5	54.6	52.1	51.8	53	52	52	56.6	51.3	50.8	49.9	
Asparagus	50.5	50.3	50.8	50.4	51.1	51.6	51.6	50.7	49.6	51.5	52.5	52.6	52.6	52.6	50.3	50.1	50.9	50.6	50.4	53.4	49.3	49	48.2	60.1

Supplementary Figure S3. The pairwise distances of the 25 plant species involved in the HT events. The names for genus or species are presented on the left and at the bottom side of the panel, respectively. The pairwise distances are presented with percent identity. The genus names are presented except for *Quercus* and *Vitis* genera that contain multiple species. The color intensity from green to red is proportional to the identity.

Asp_HT-1

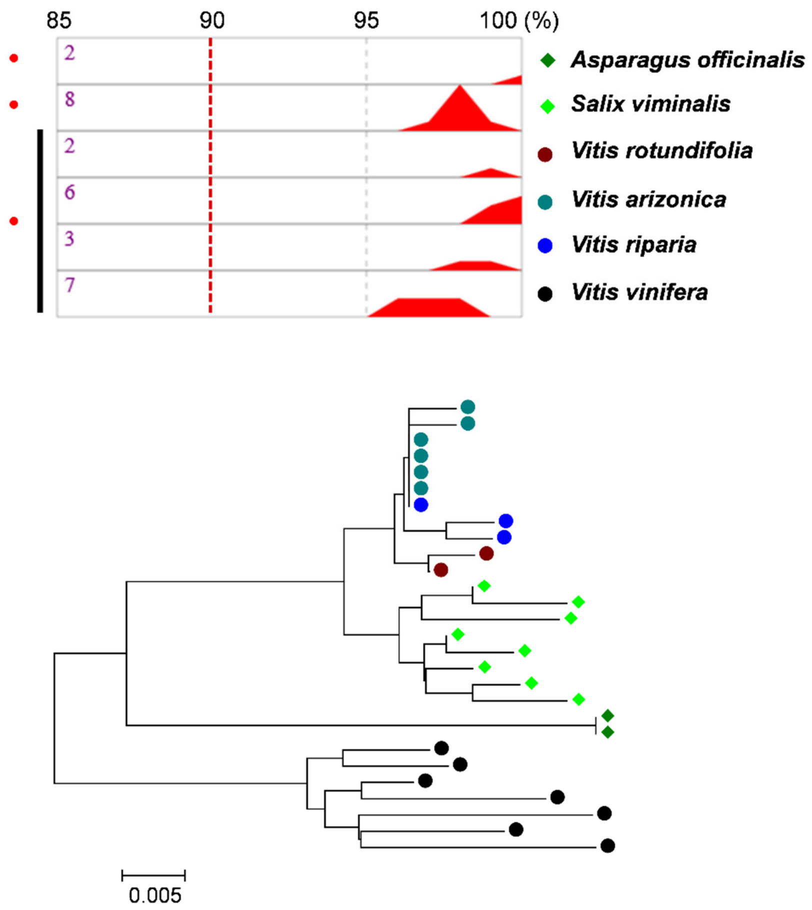

Supplementary Figure S4. *Cont.*

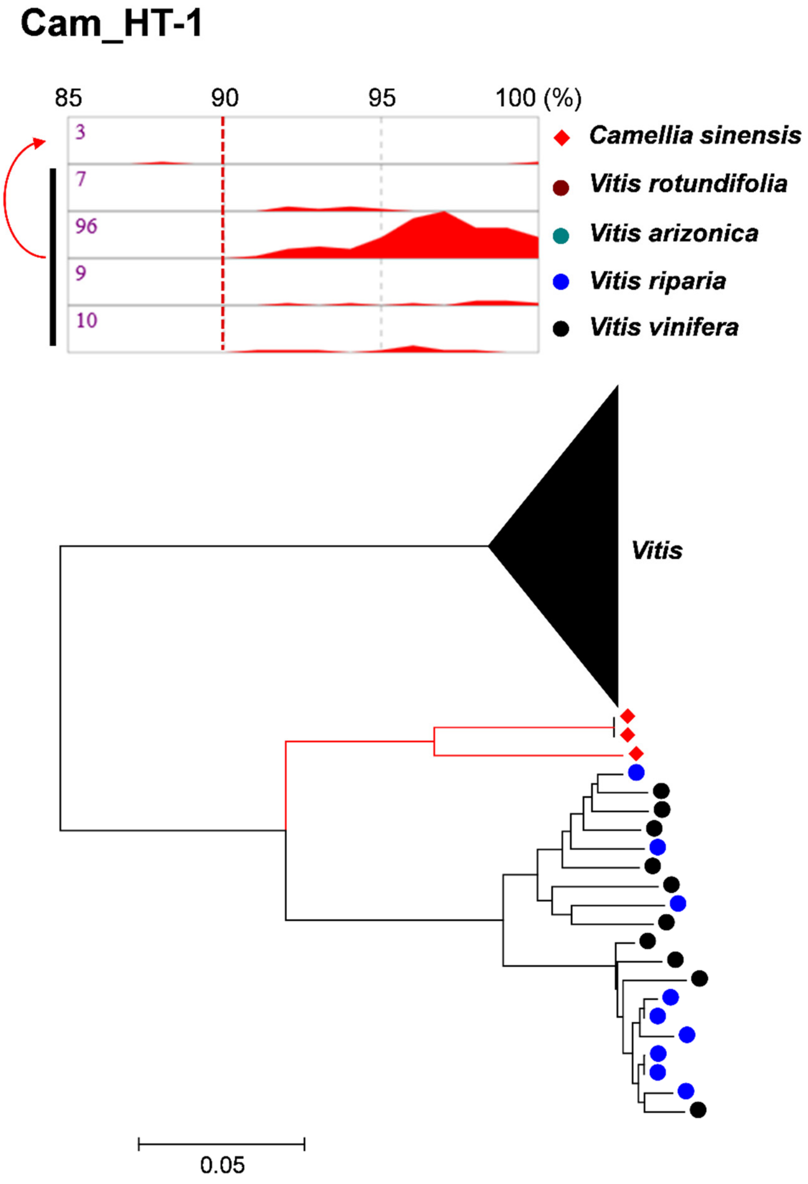

Supplementary Figure S4. *Cont.*

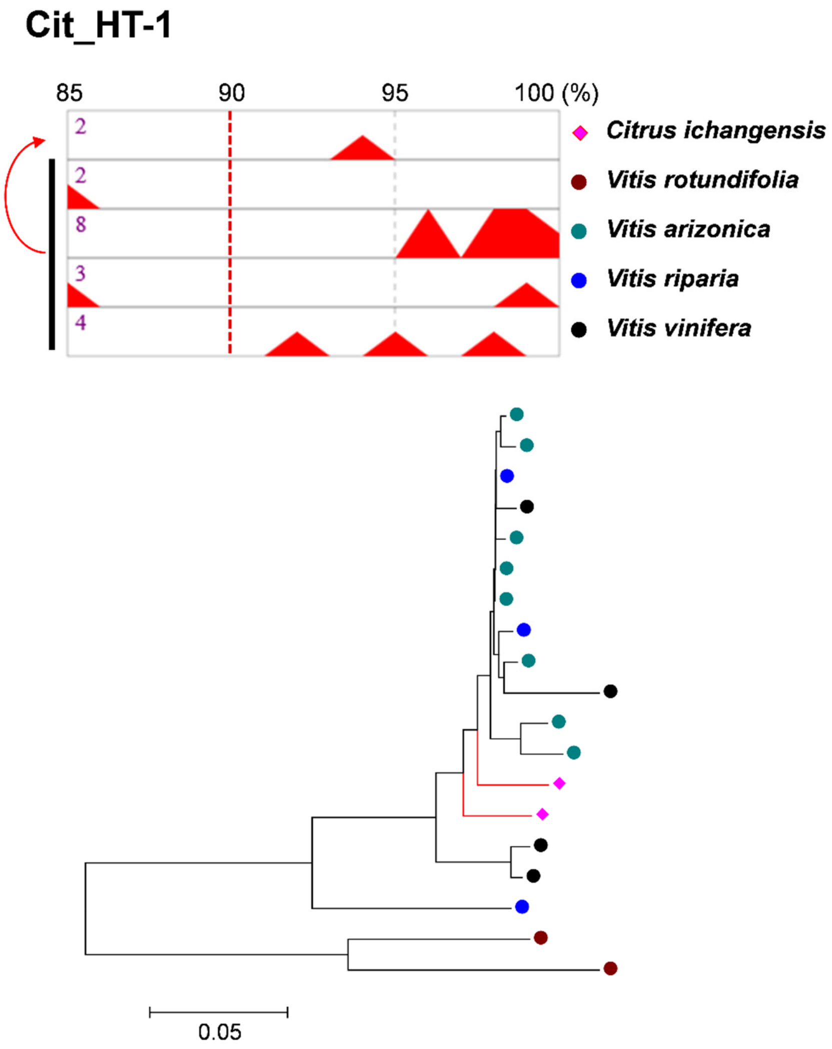

Supplementary Figure S4. *Cont.*

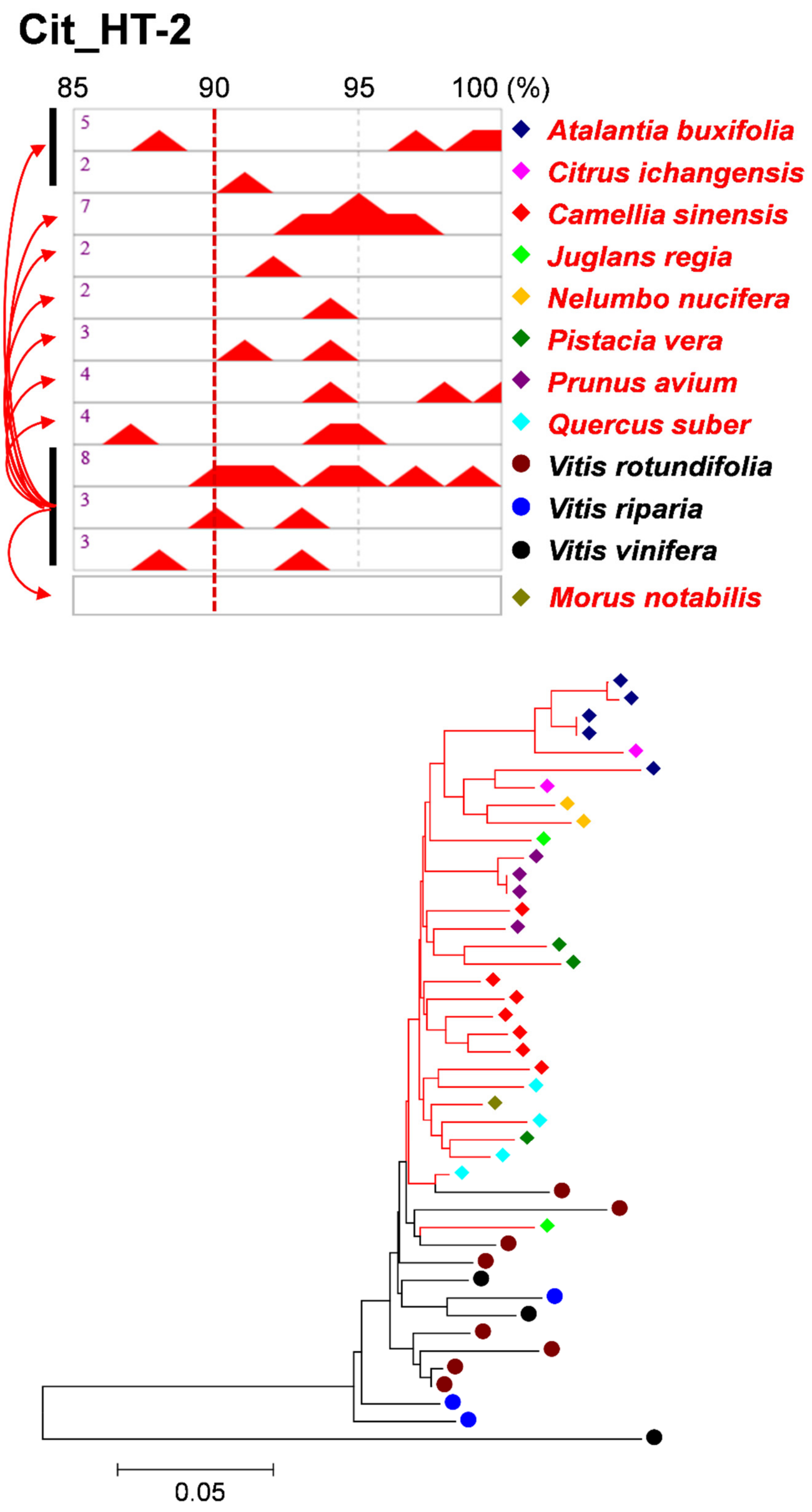

Supplementary Figure S4. *Cont.*

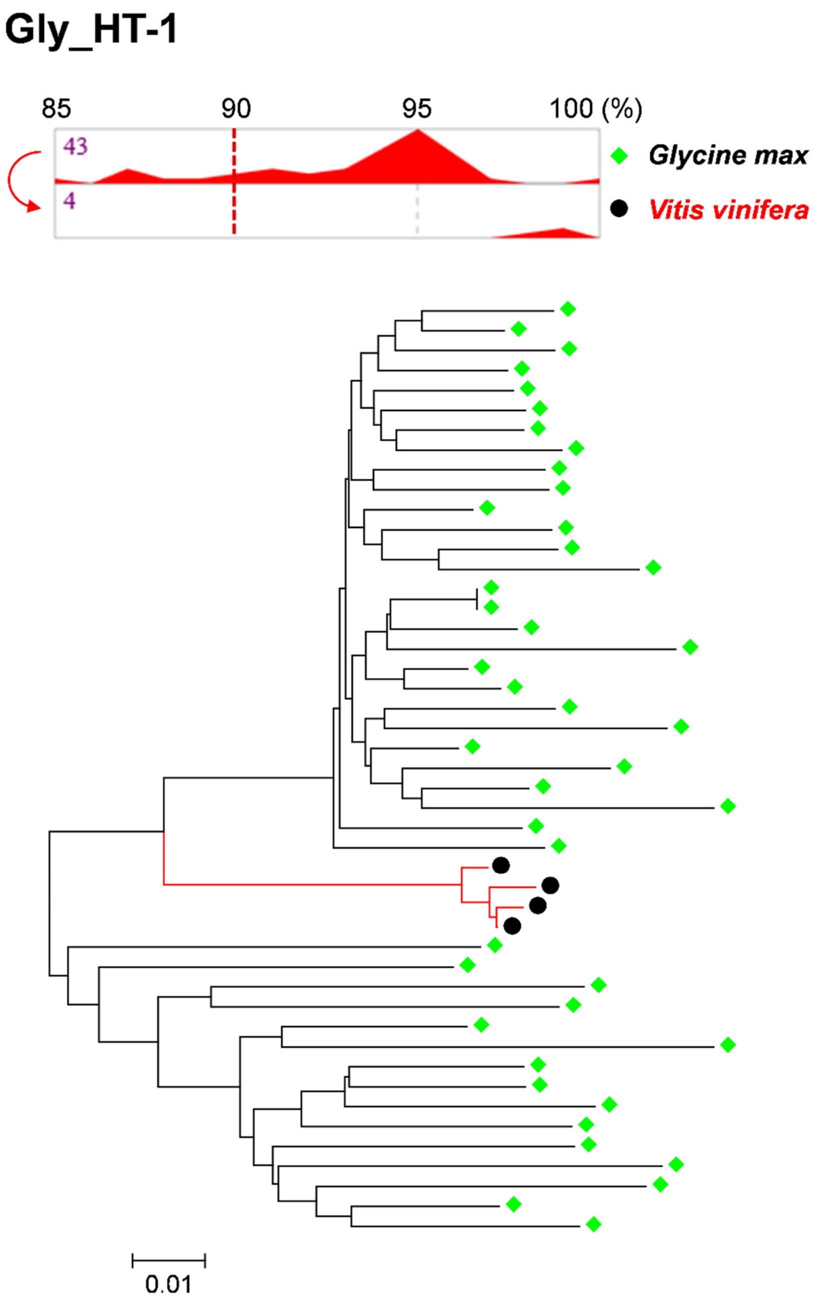

Supplementary Figure S4. *Cont.*

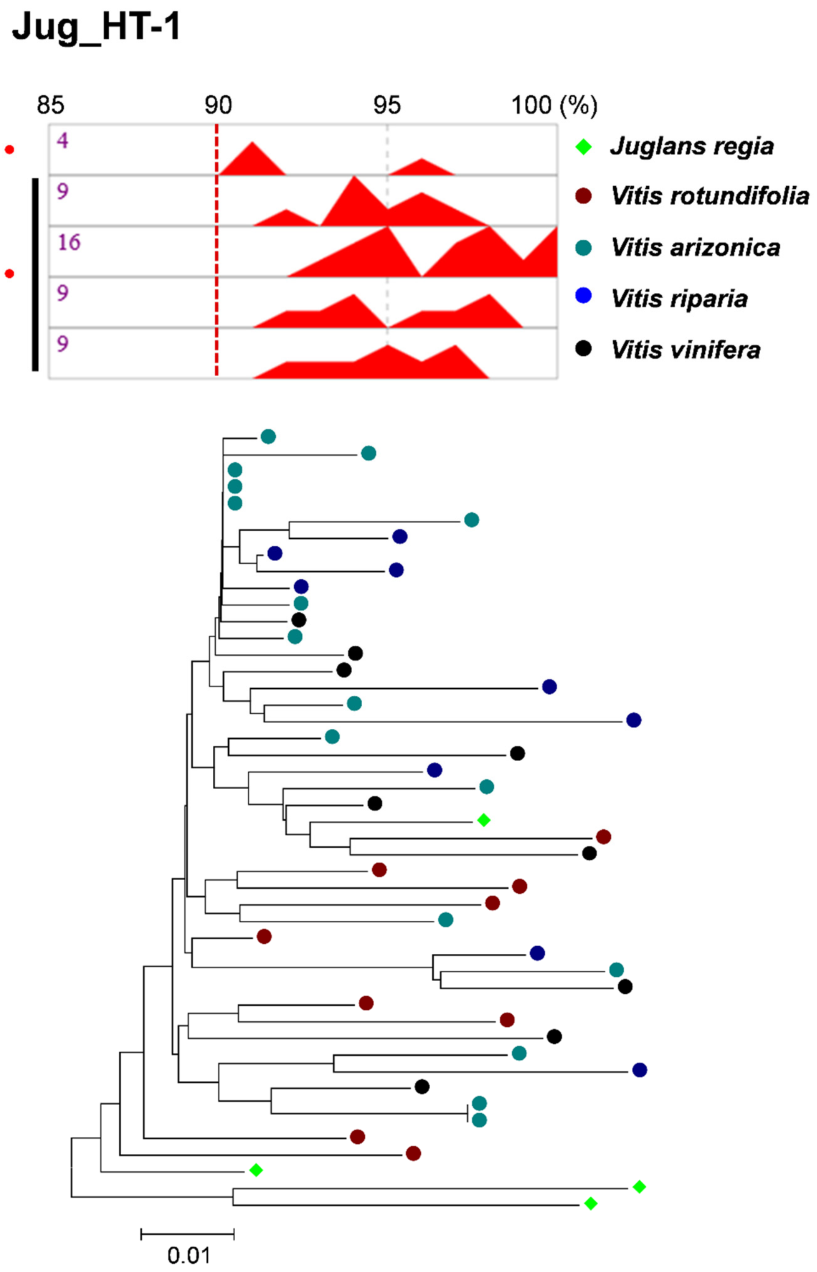

Supplementary Figure S4. *Cont.*

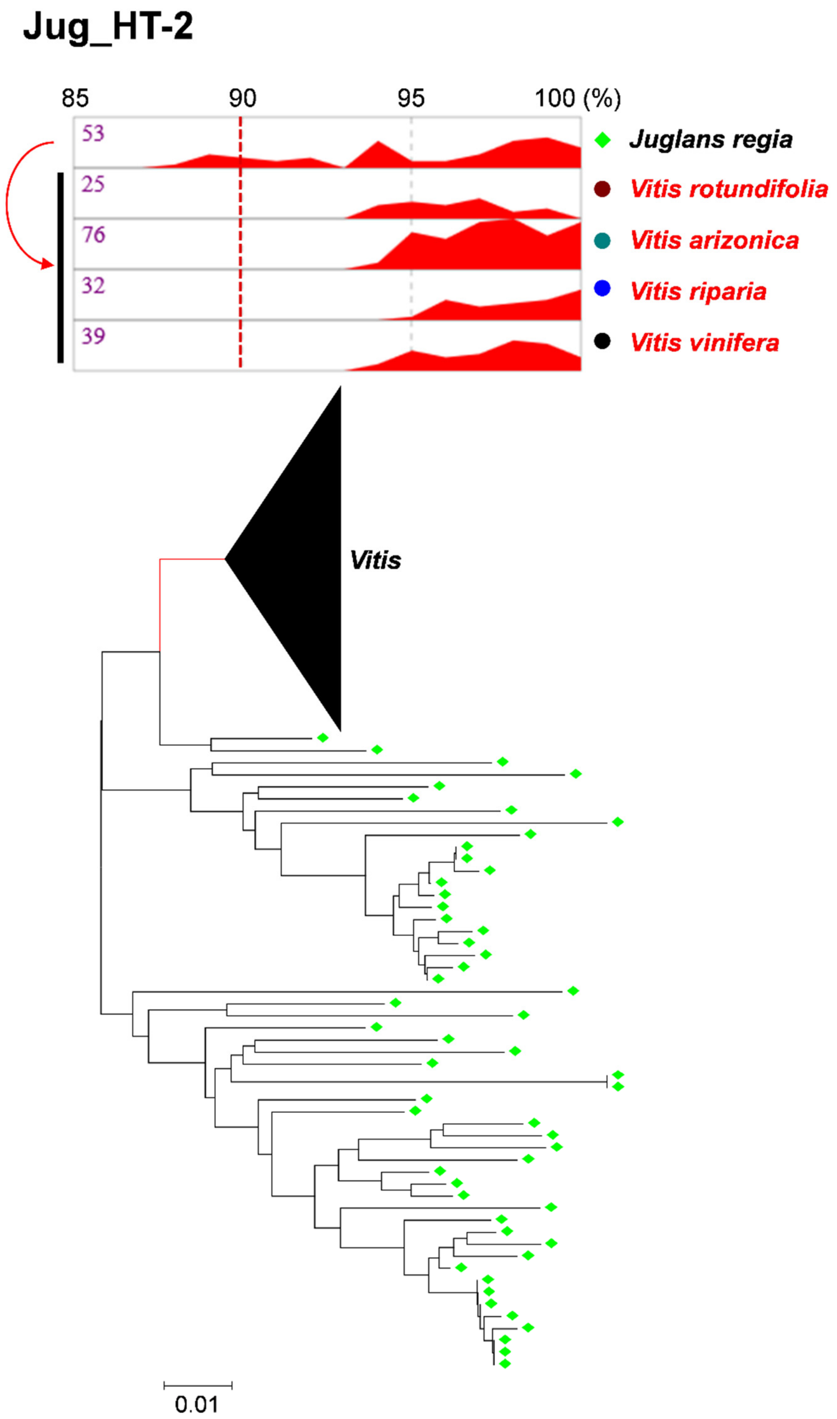

Supplementary Figure S4. *Cont.*

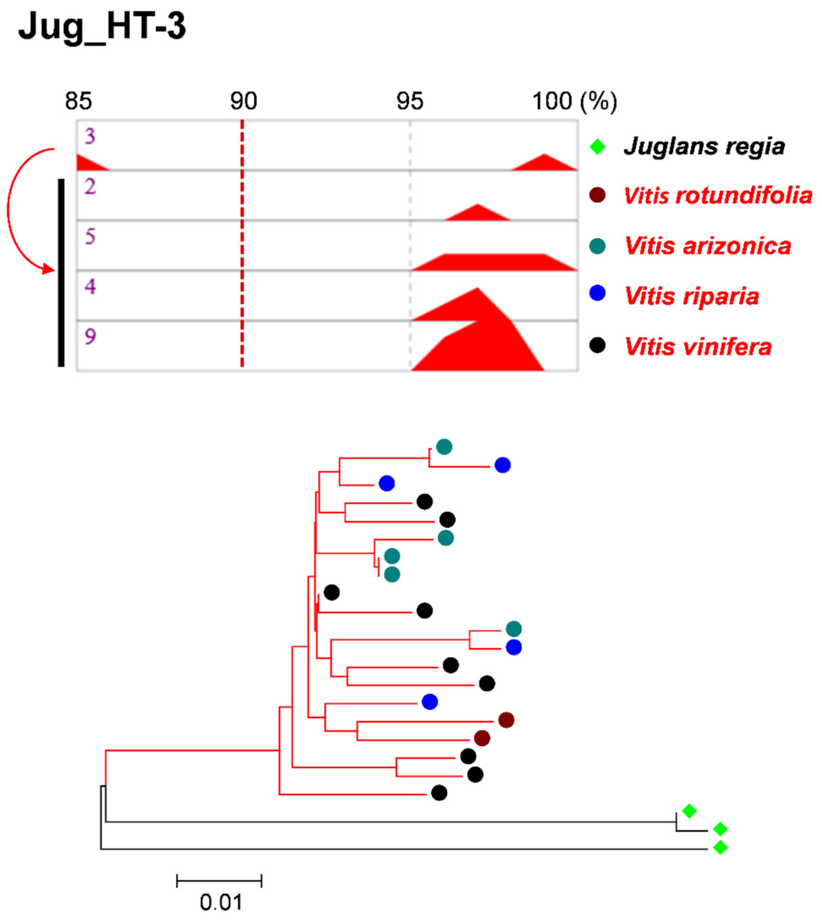

Supplementary Figure S4. *Cont.*

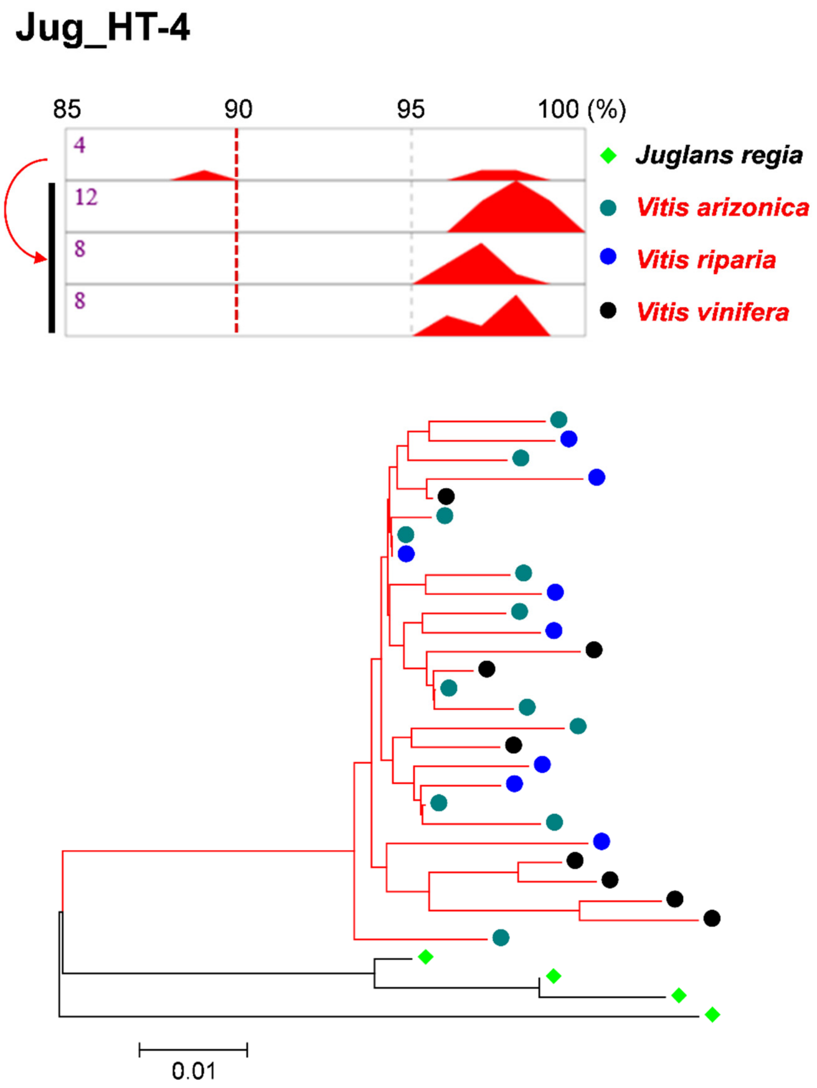

Supplementary Figure S4. *Cont.*

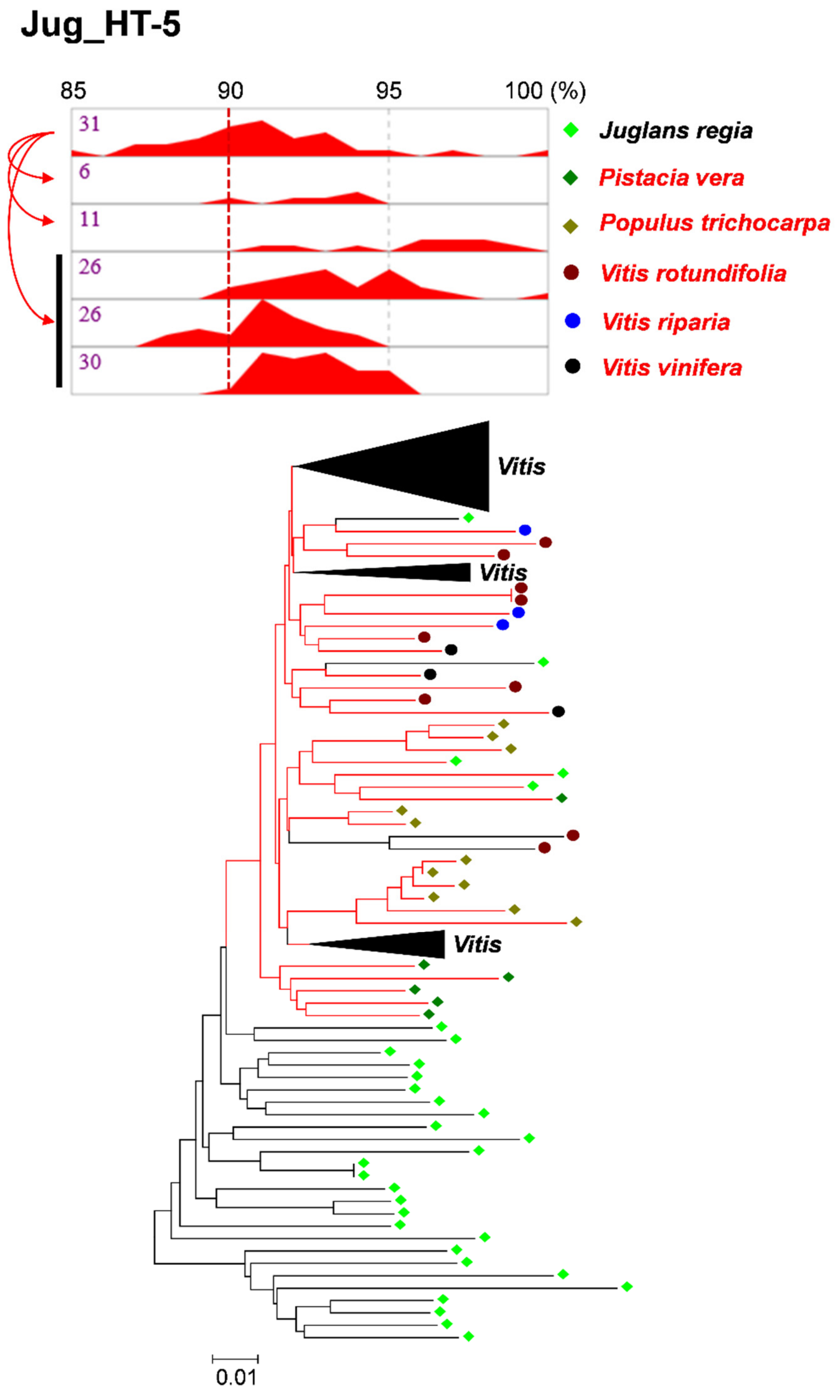

Supplementary Figure S4. *Cont.*

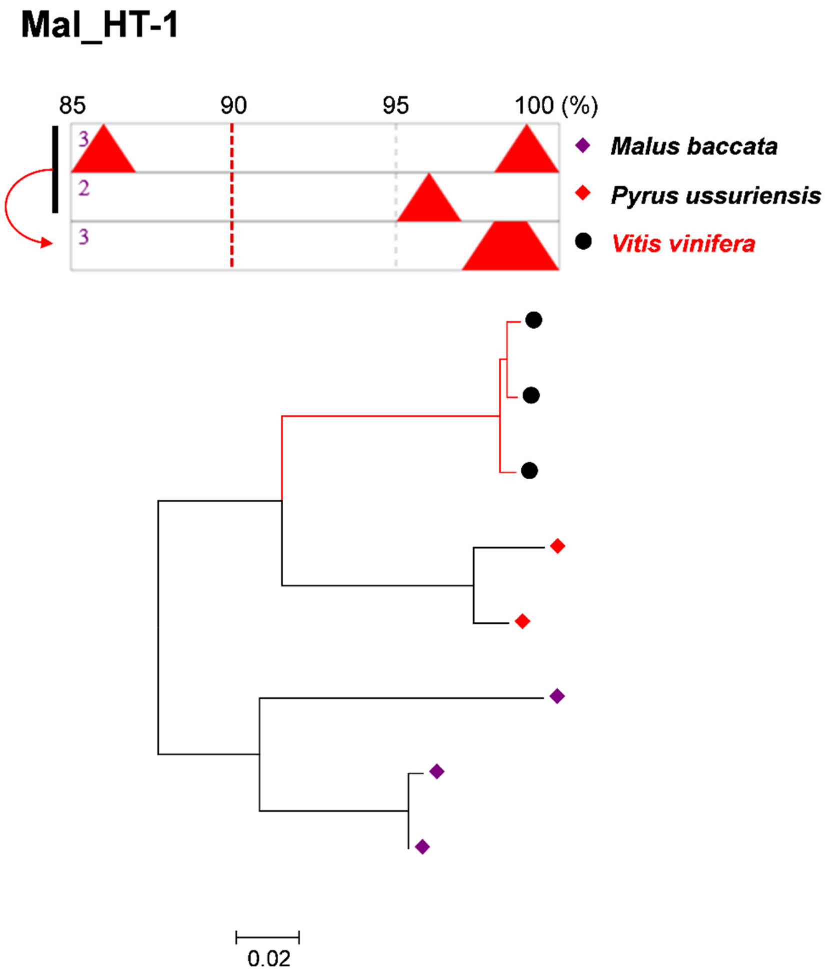

Supplementary Figure S4. *Cont.*

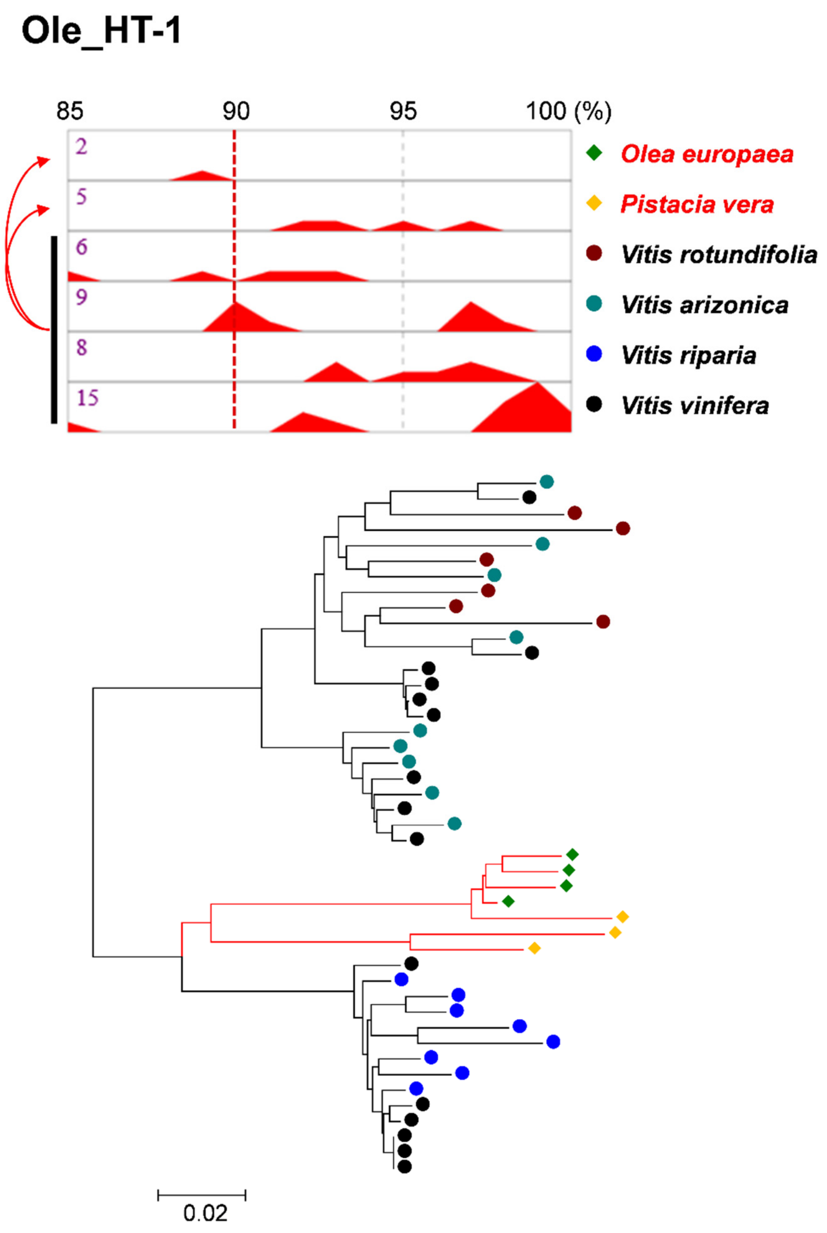

Supplementary Figure S4. *Cont.*

Pha_HT-1

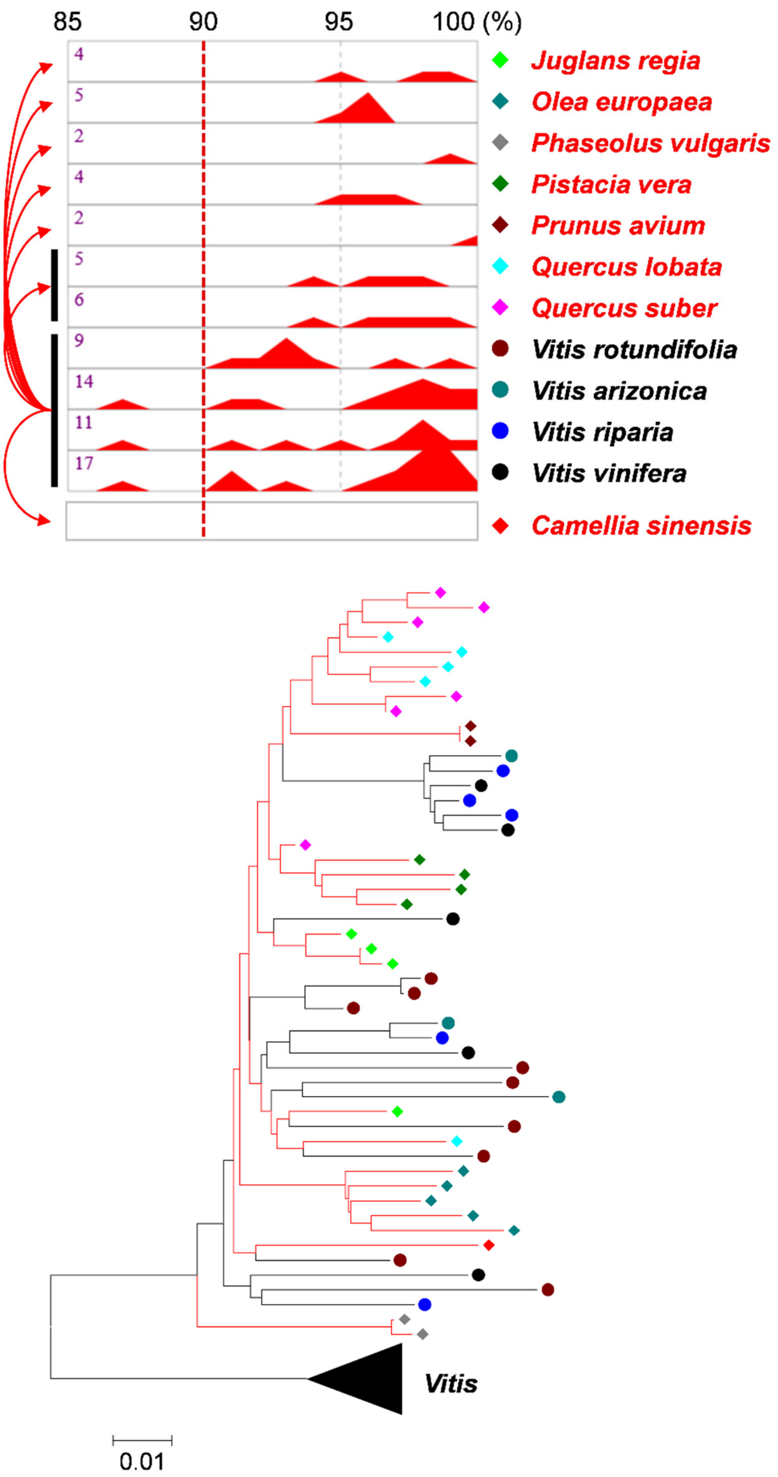

Supplementary Figure S4. *Cont.*

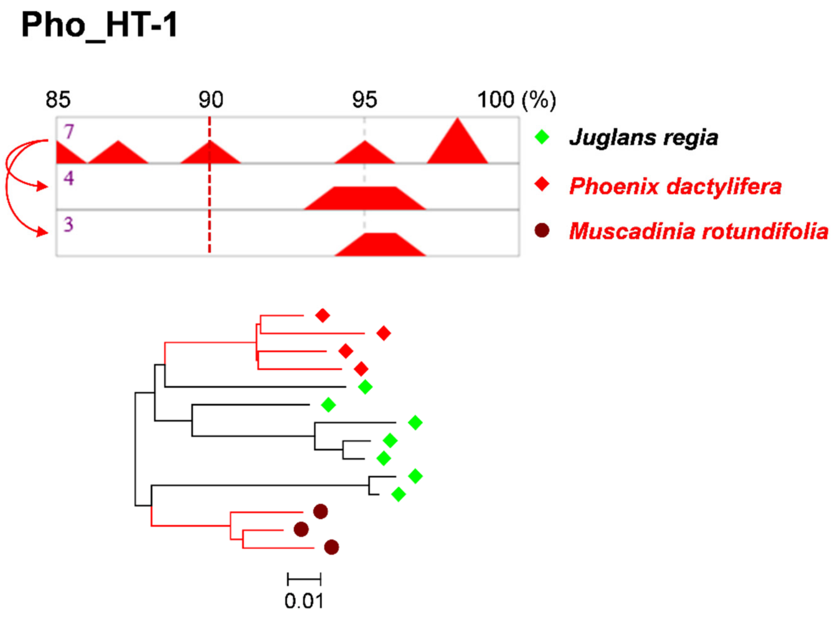

Supplementary Figure S4. *Cont.*

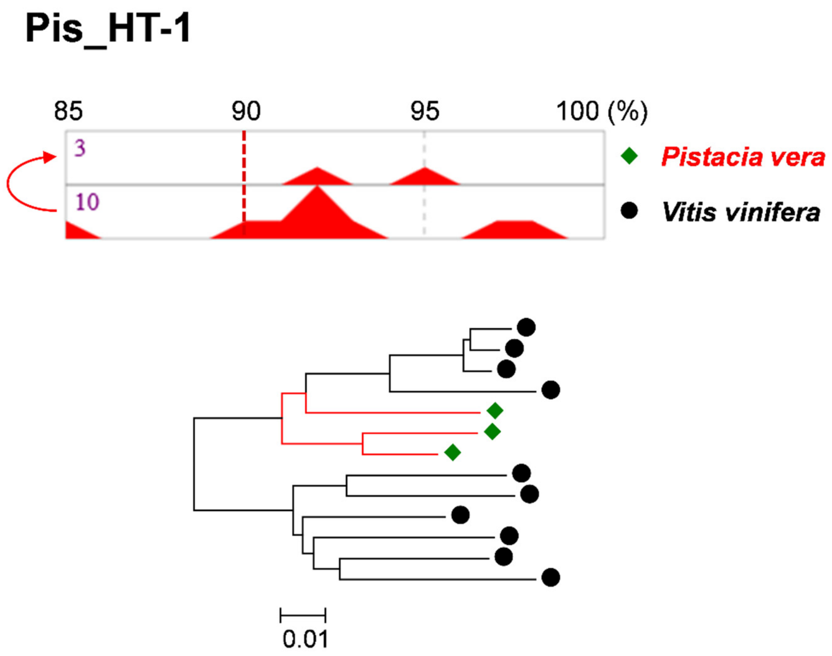

Supplementary Figure S4. *Cont.*

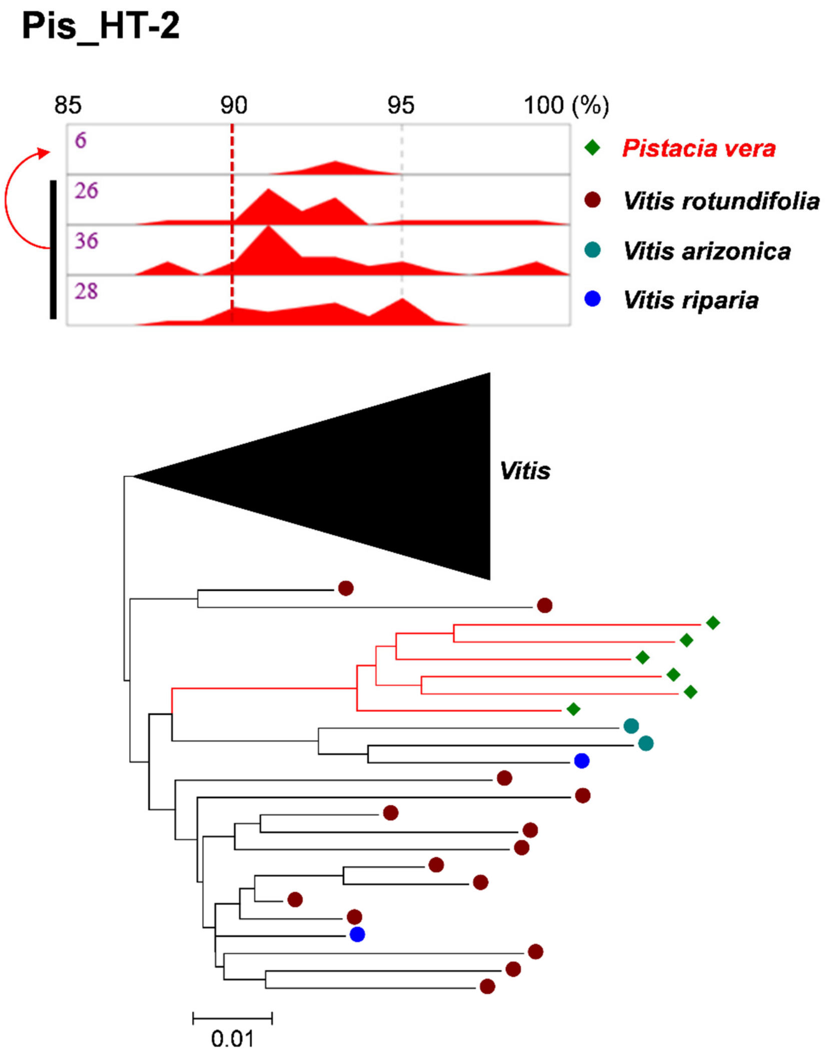

Supplementary Figure S4. *Cont.*

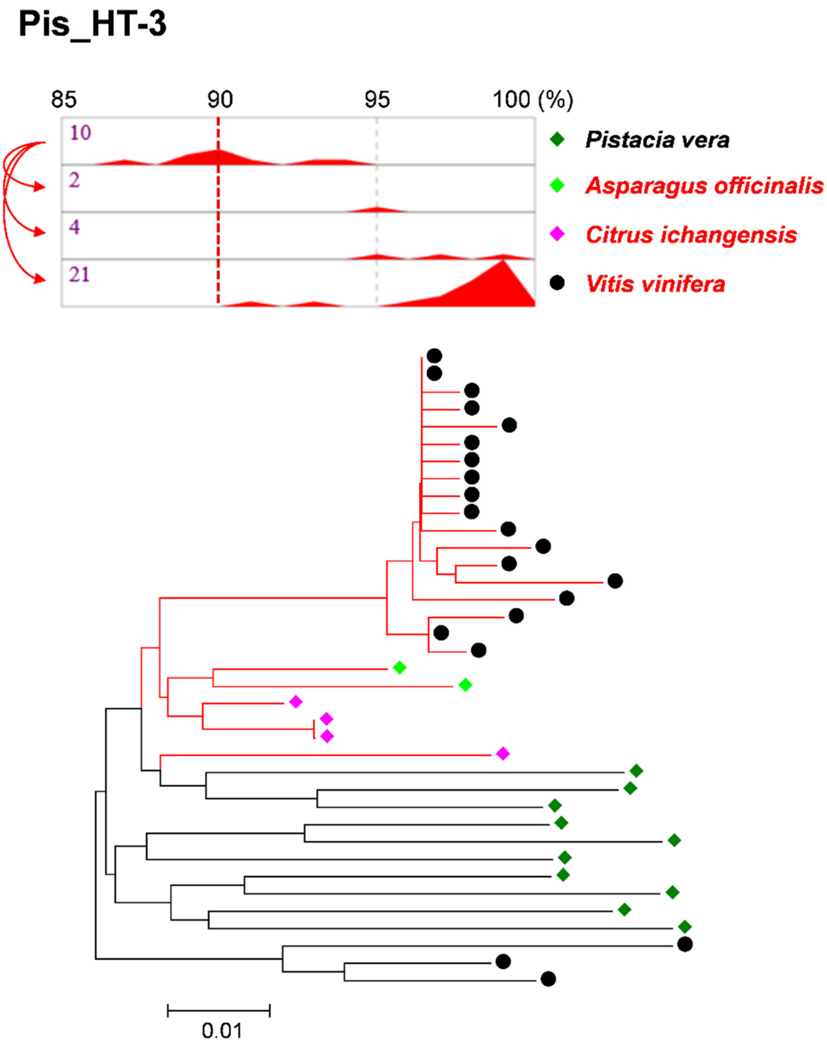

Supplementary Figure S4. *Cont.*

Pis_HT-4

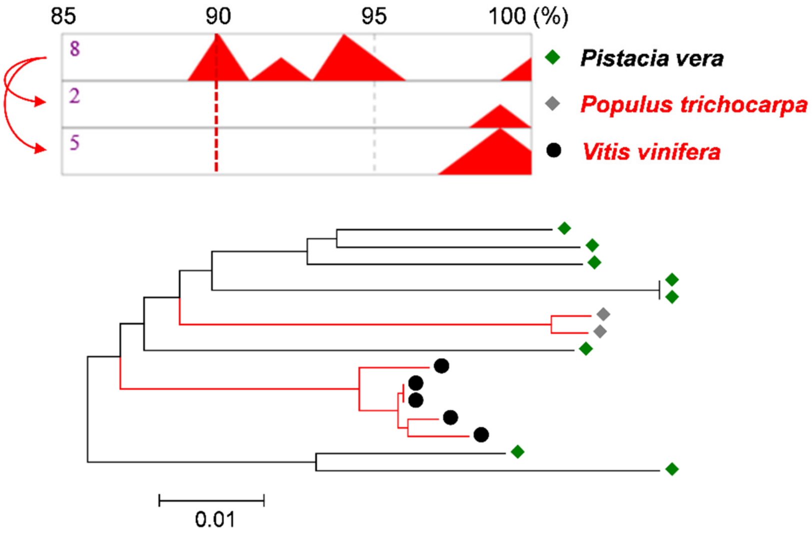

Supplementary Figure S4. *Cont.*

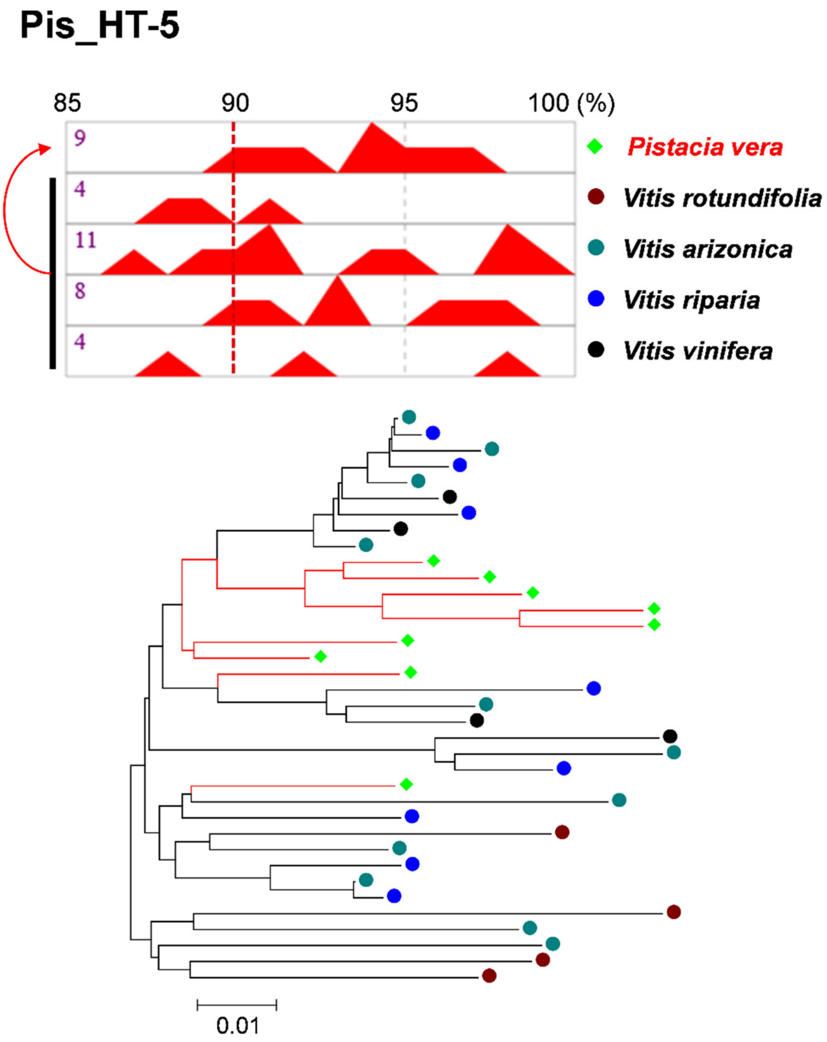

Supplementary Figure S4. *Cont.*

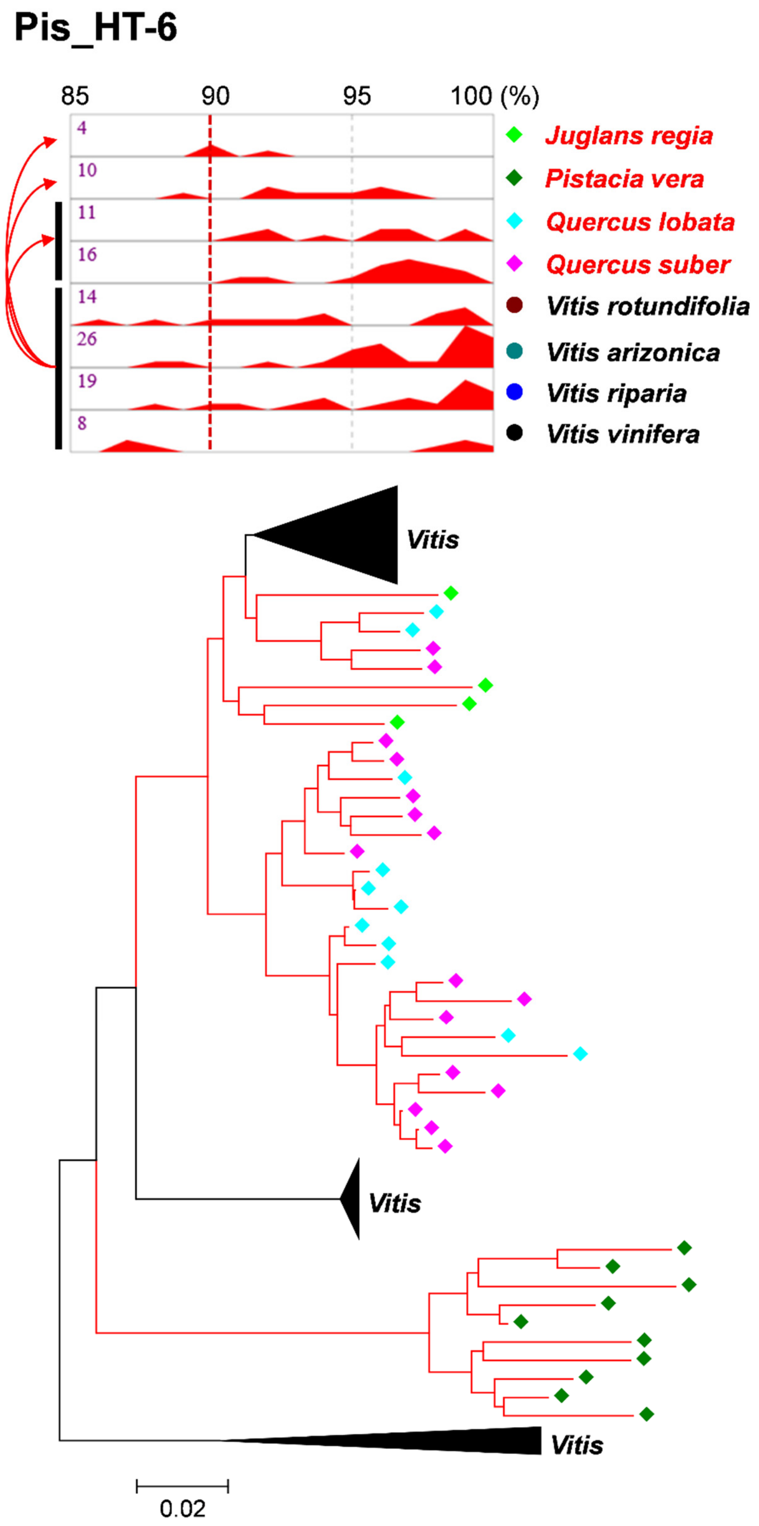

Supplementary Figure S4. *Cont.*

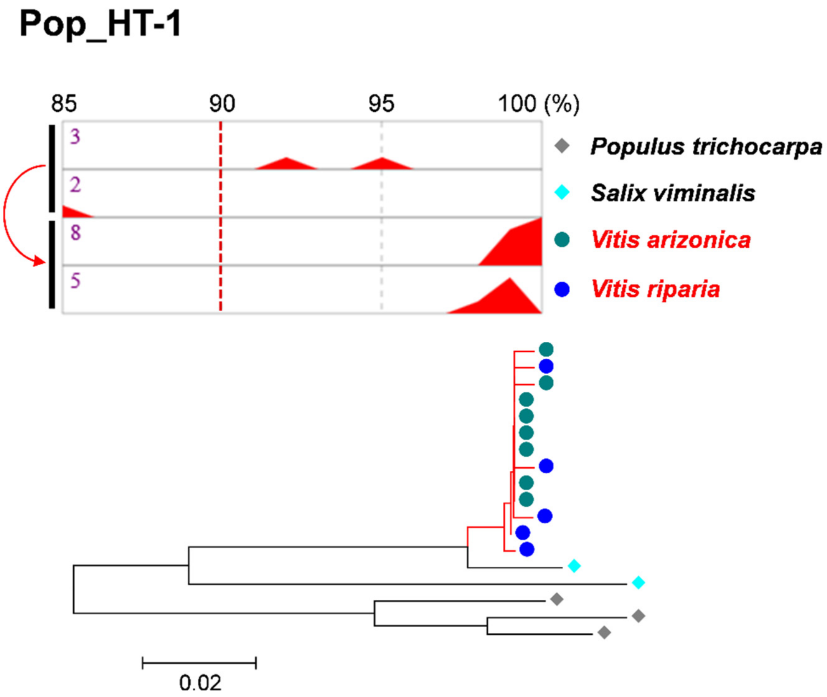

Supplementary Figure S4. *Cont.*

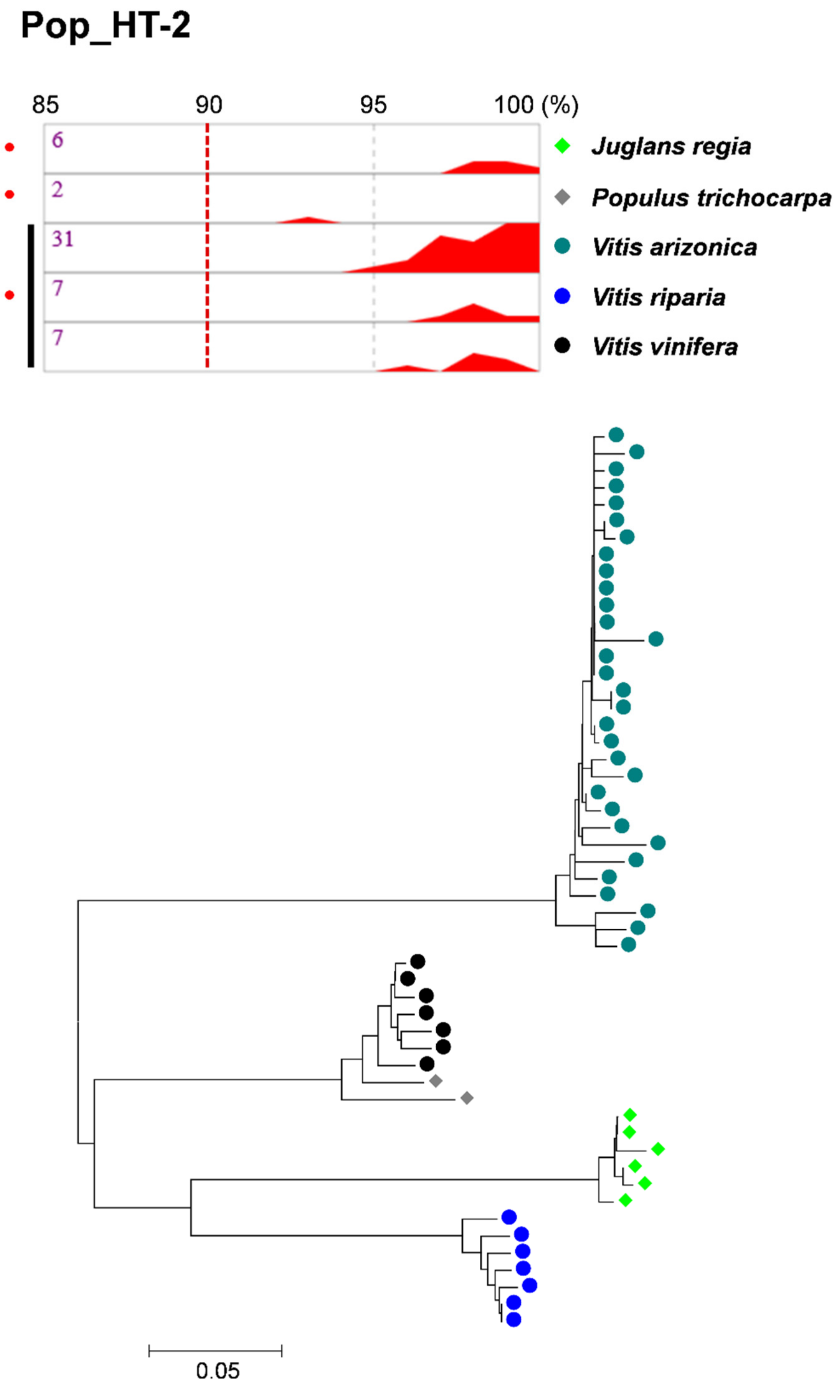

Supplementary Figure S4. *Cont.*

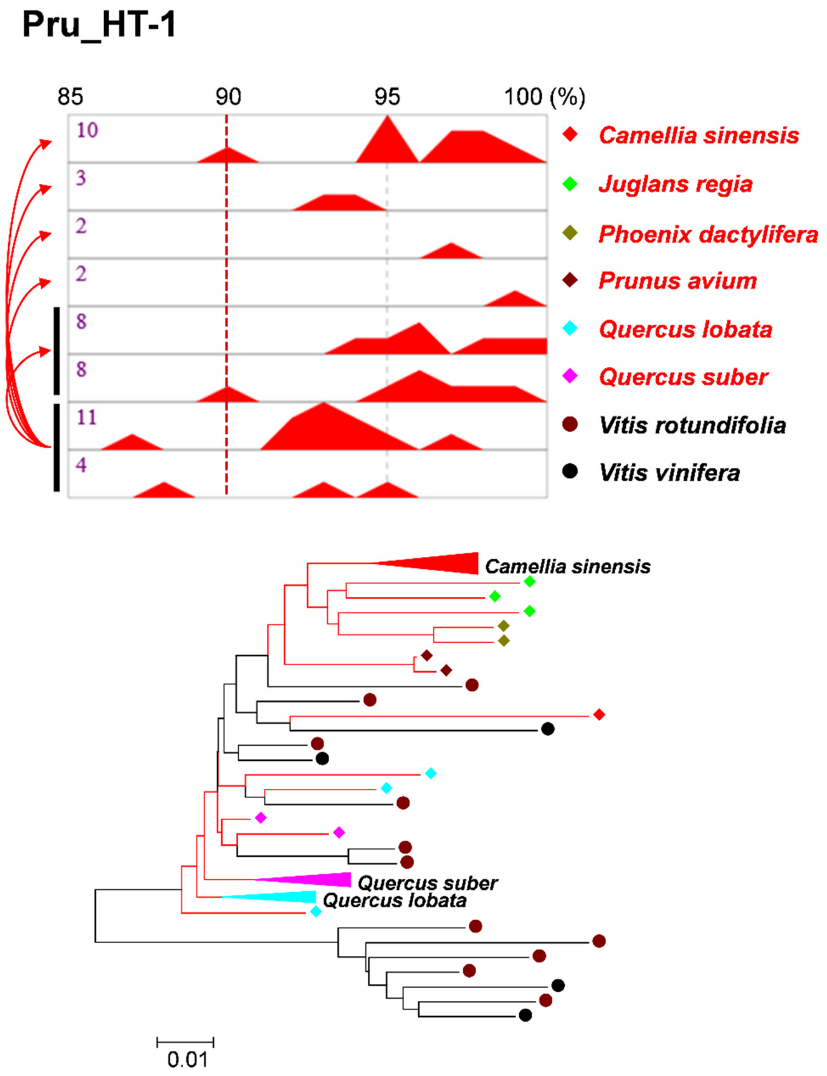

Supplementary Figure S4. *Cont.*

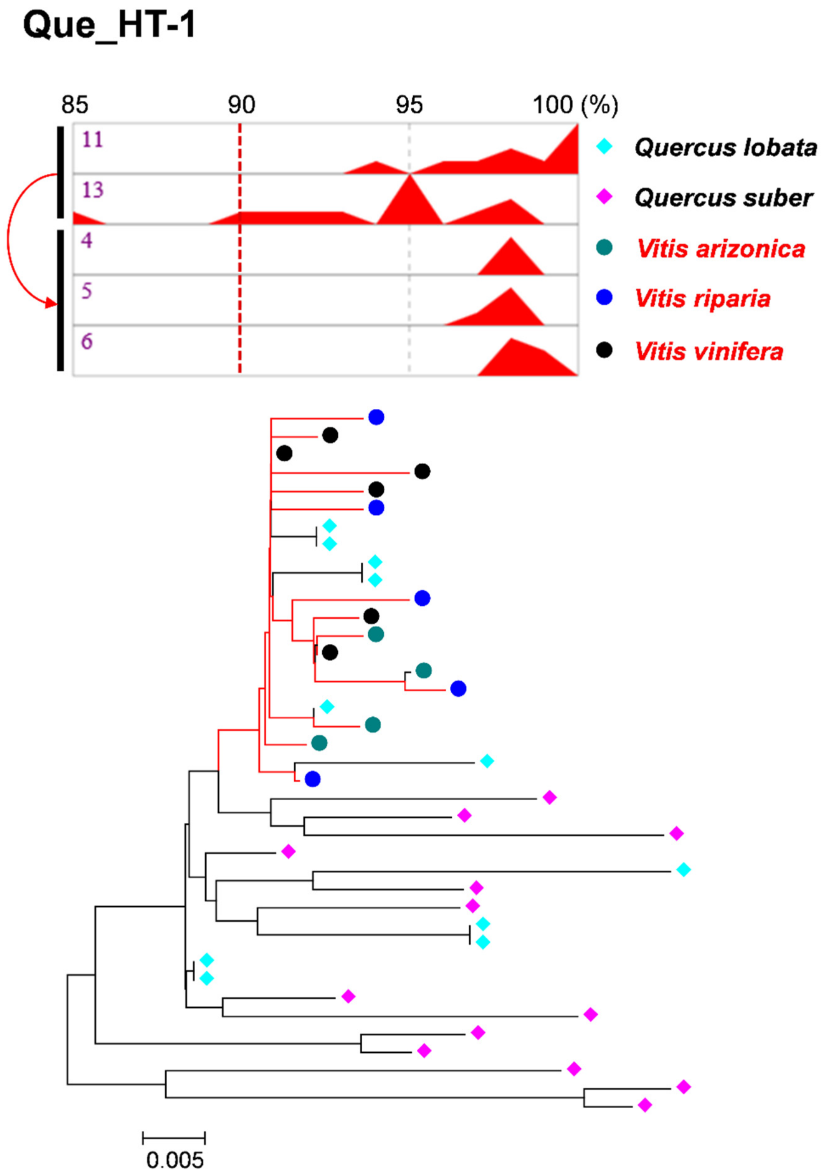

Supplementary Figure S4. *Cont.*

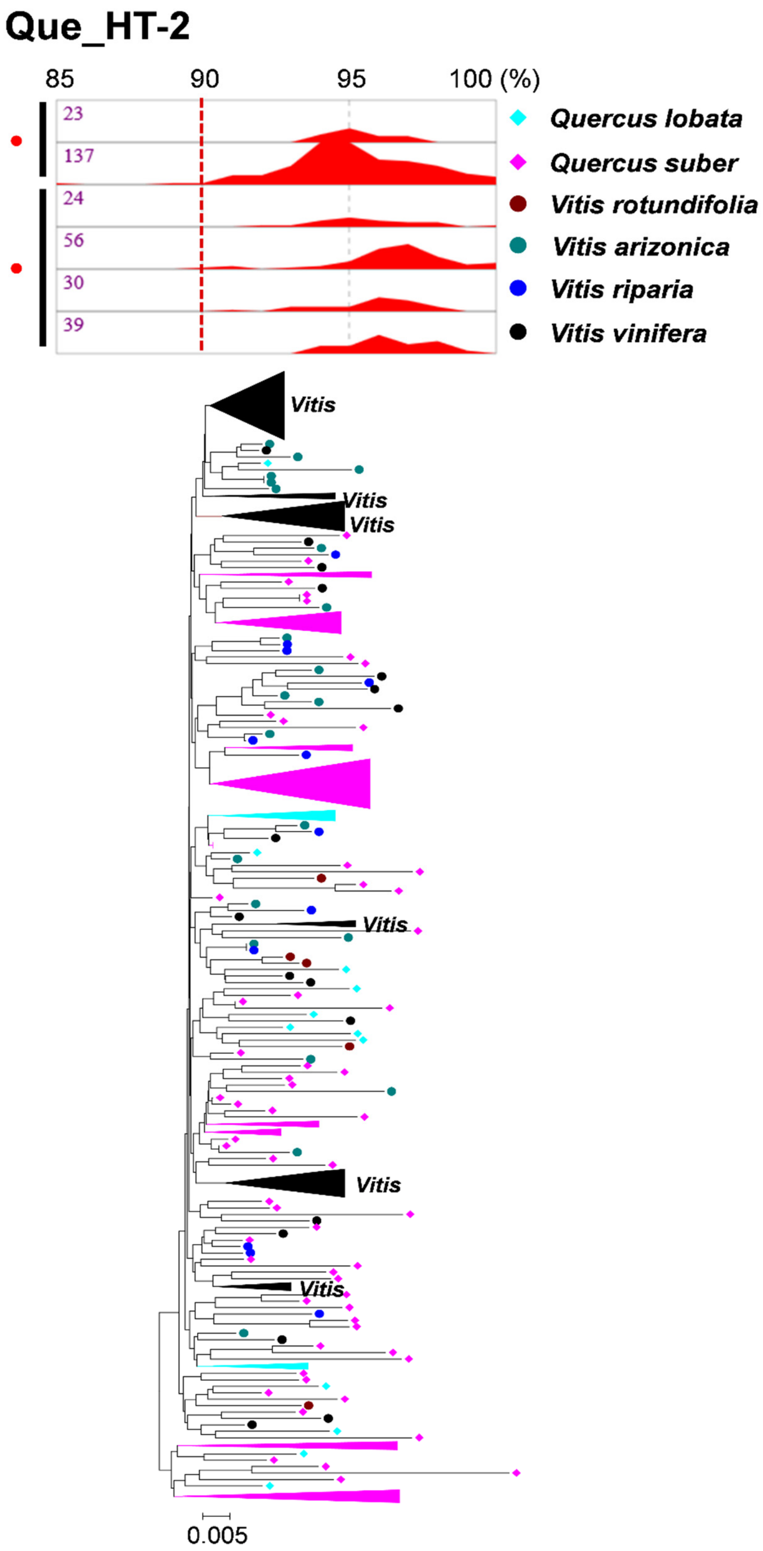

Supplementary Figure S4. *Cont.*

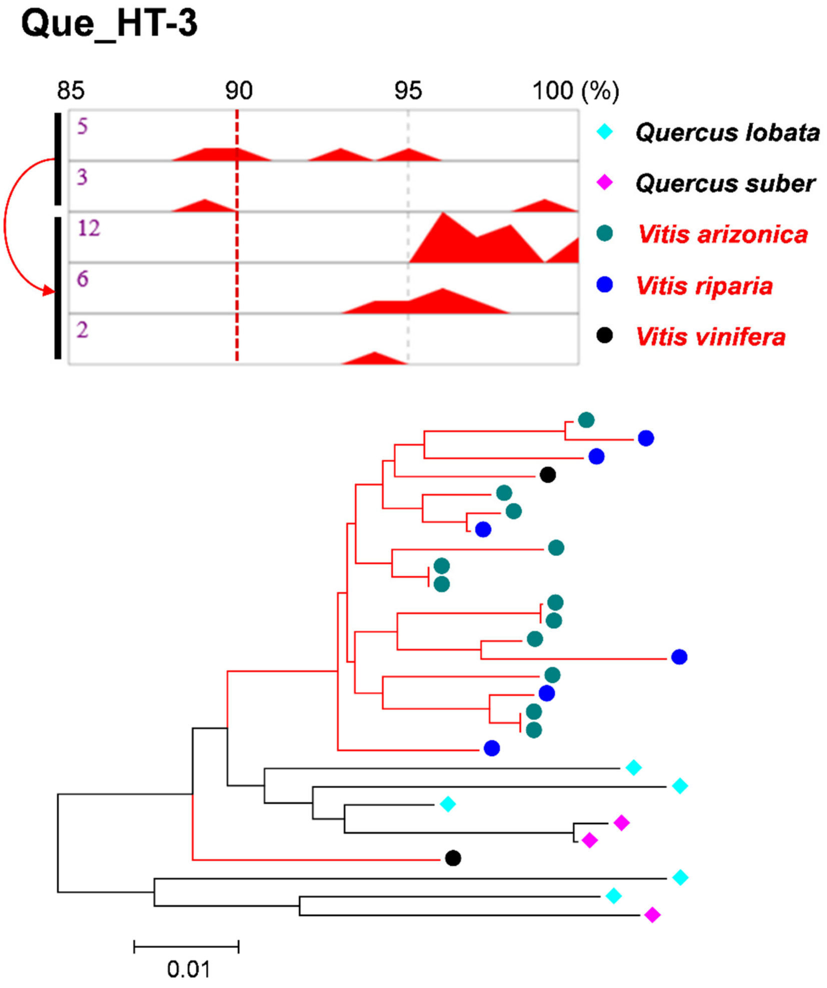

Supplementary Figure S4. *Cont.*

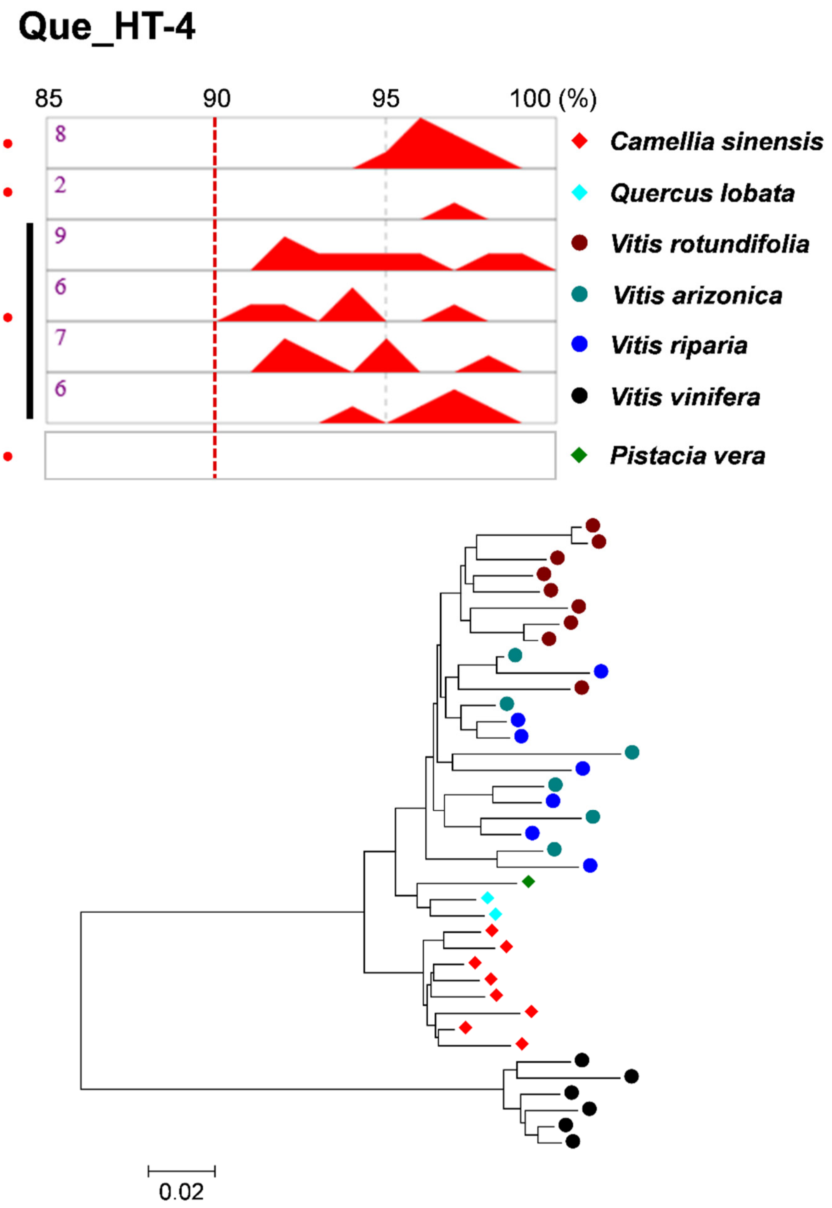

Supplementary Figure S4. *Cont.*

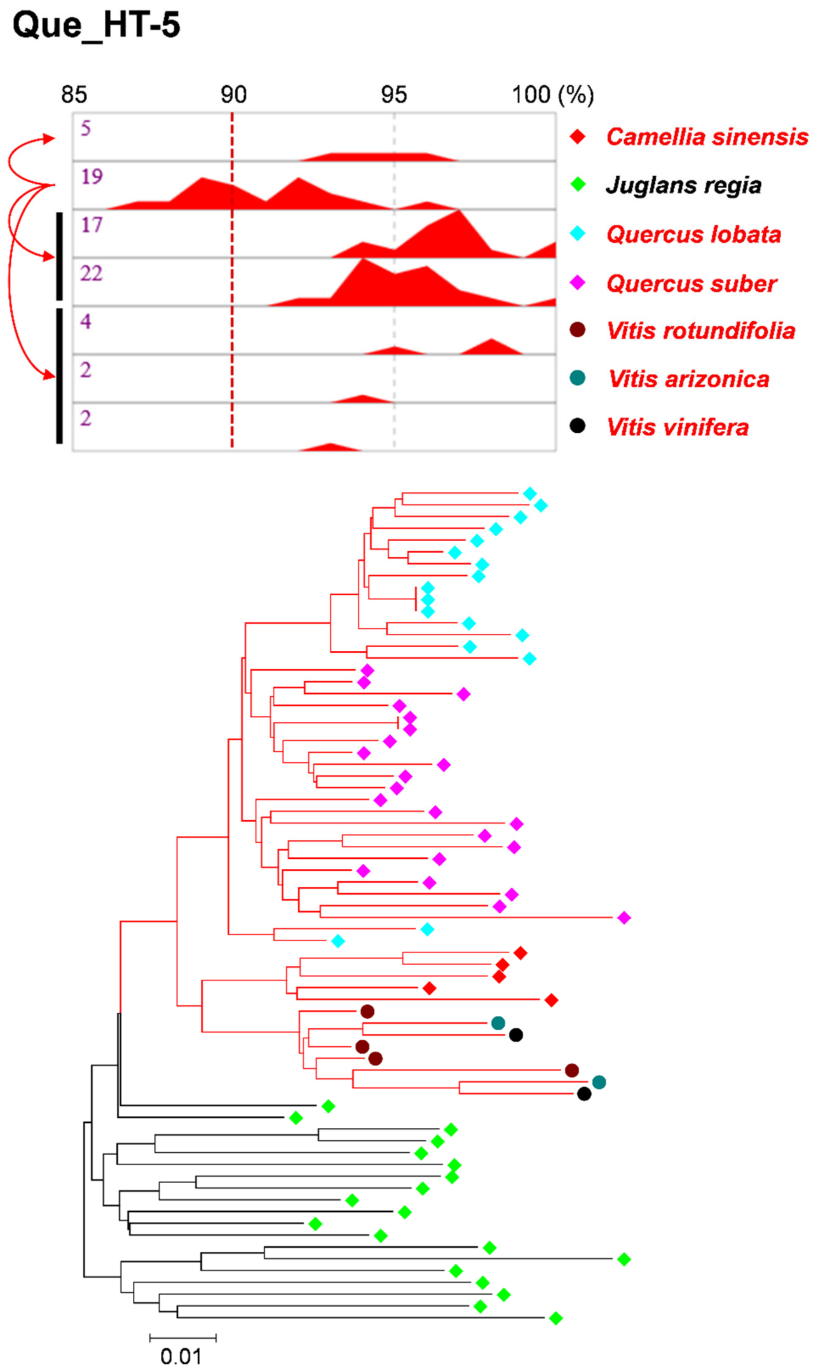

Supplementary Figure S4. *Cont.*

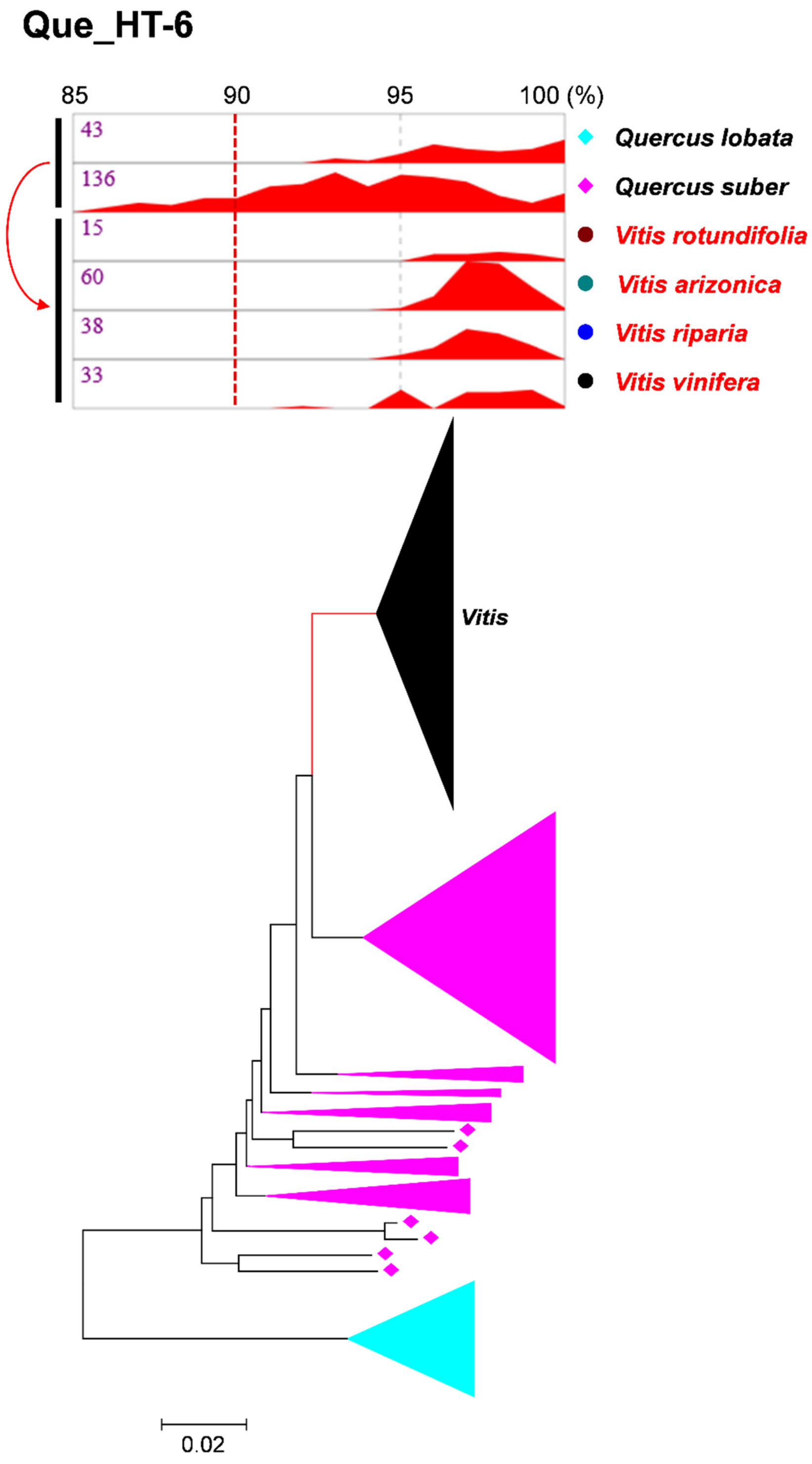

Supplementary Figure S4. *Cont.*

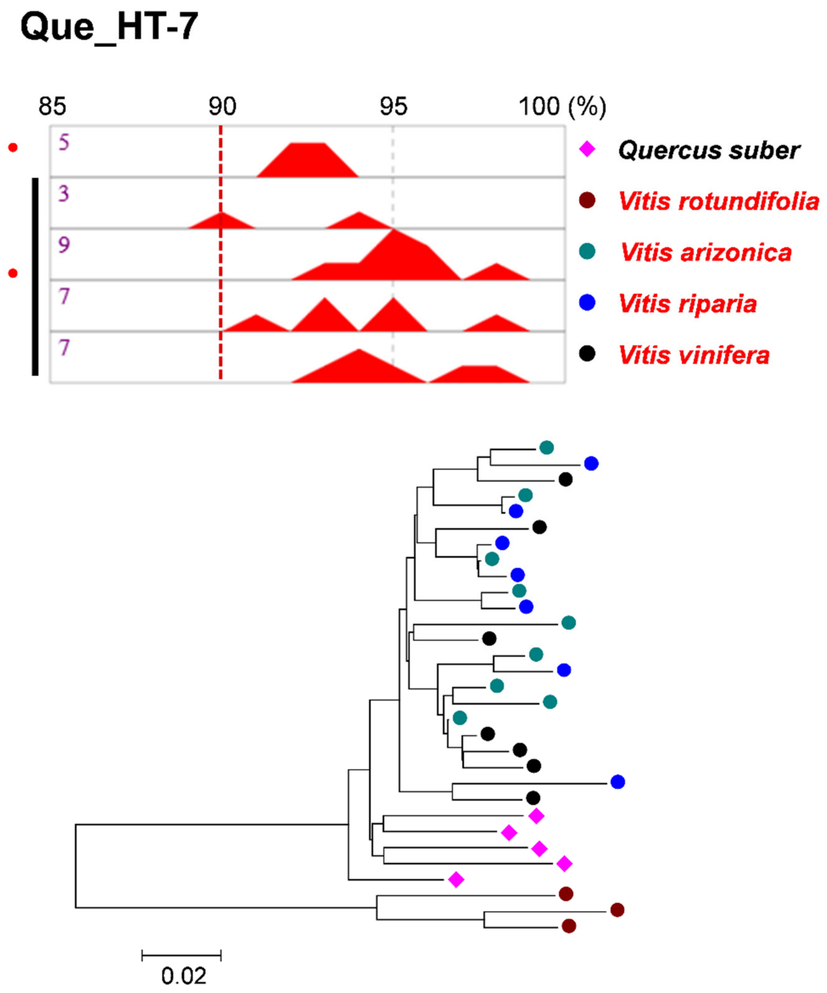

Supplementary Figure S4. *Cont.*

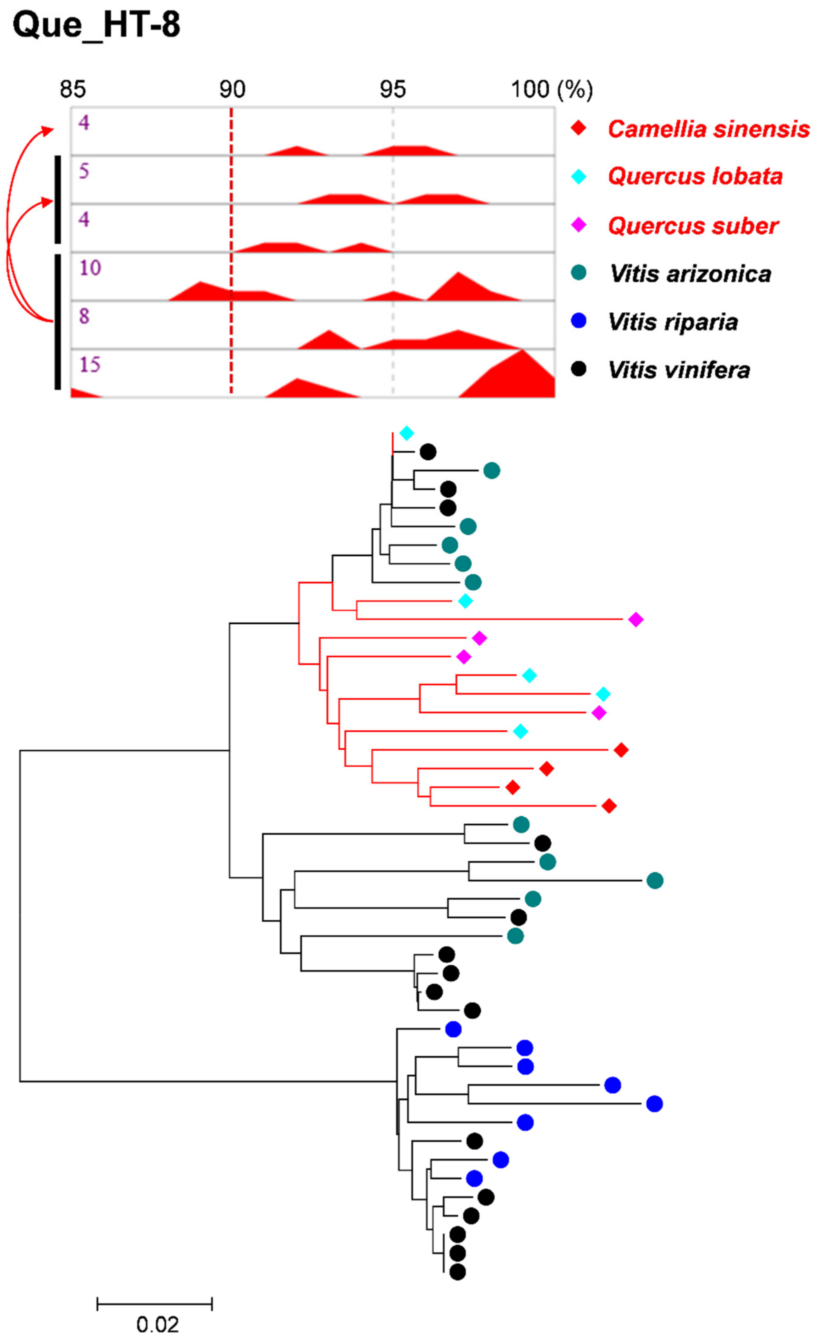

Supplementary Figure S4. *Cont.*

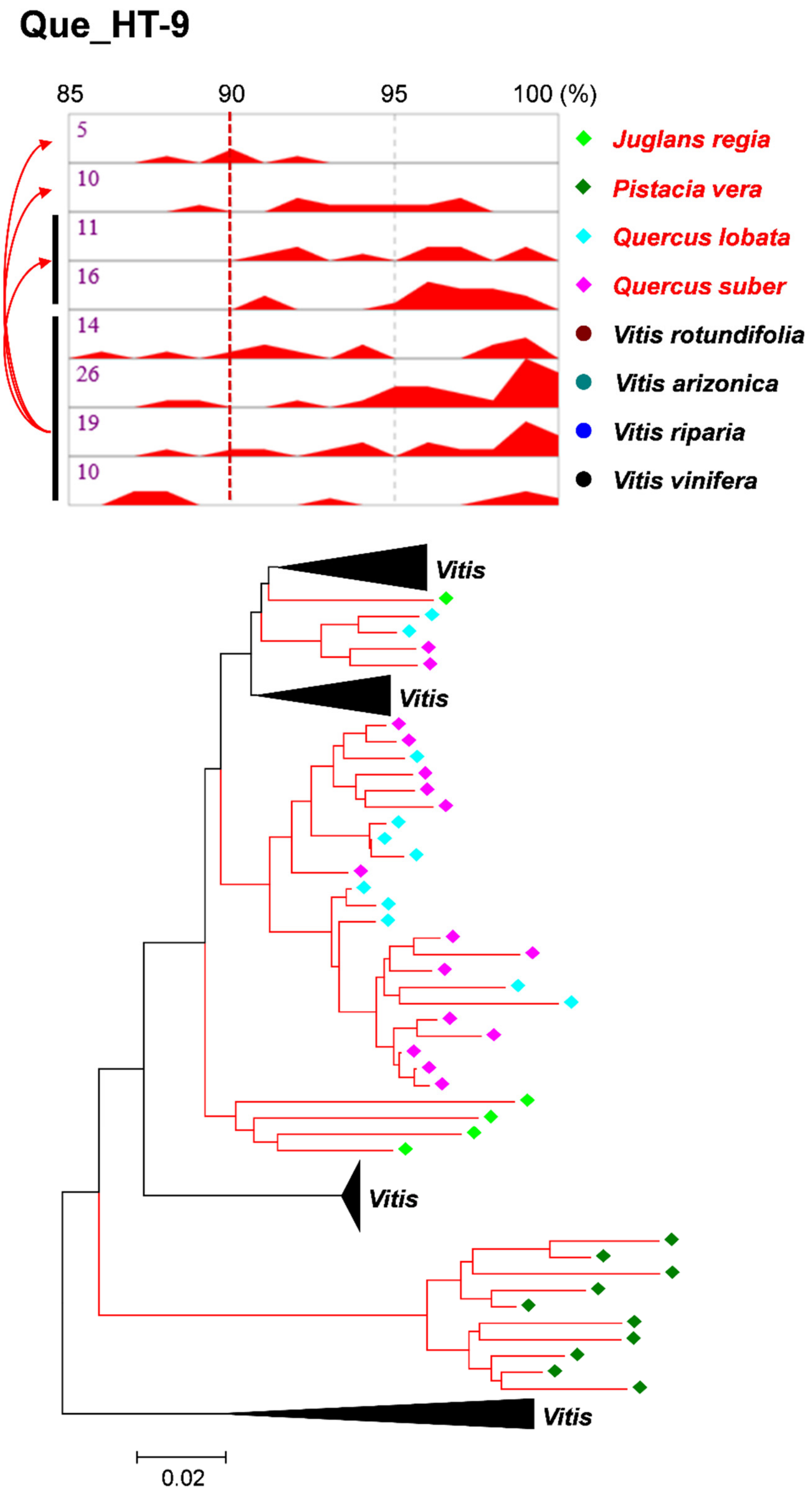

Supplementary Figure S4. *Cont.*

Rub_HT-1

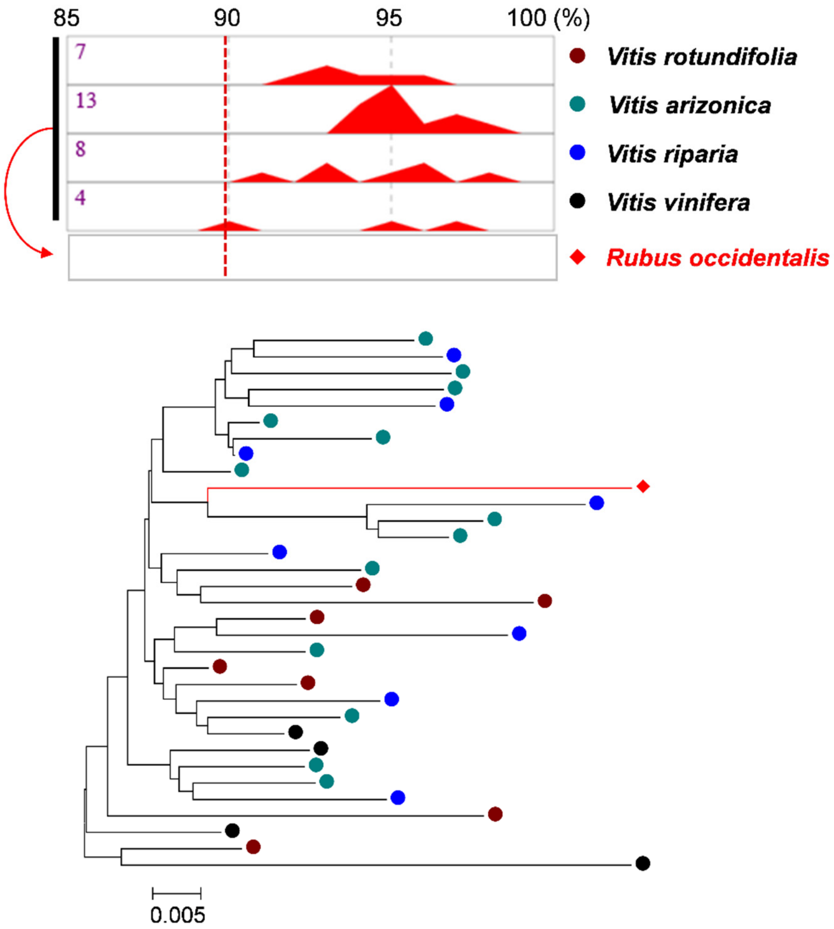

Supplementary Figure S4. *Cont.*

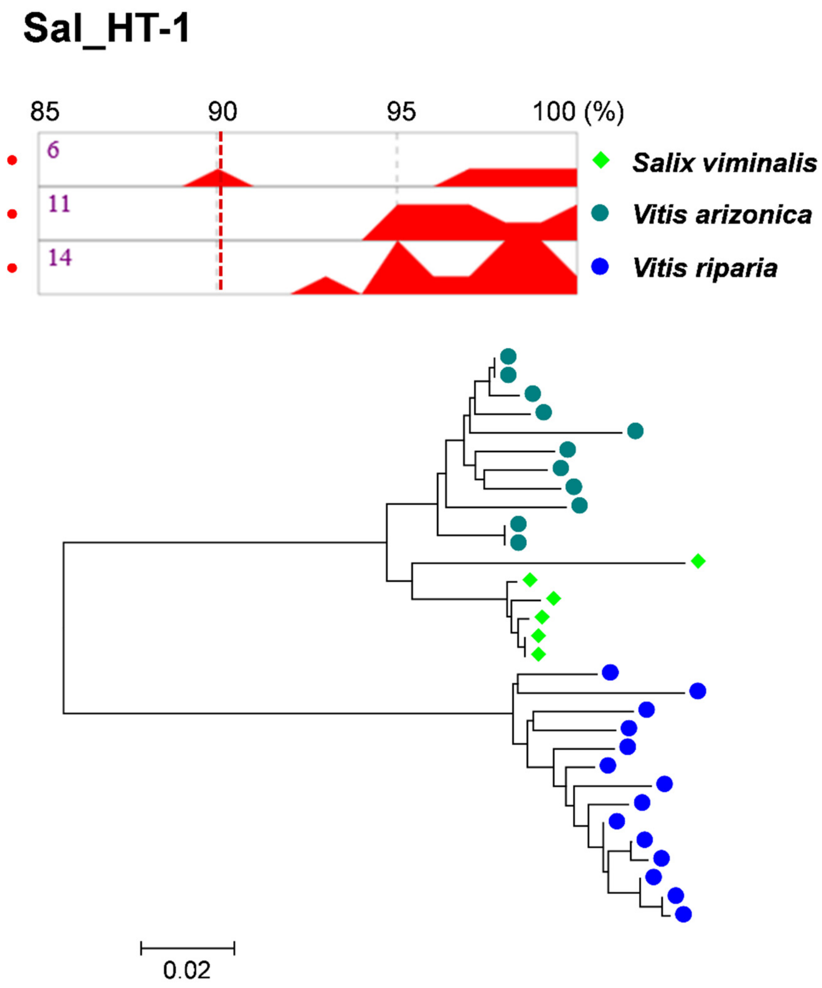

Supplementary Figure S4. *Cont.*

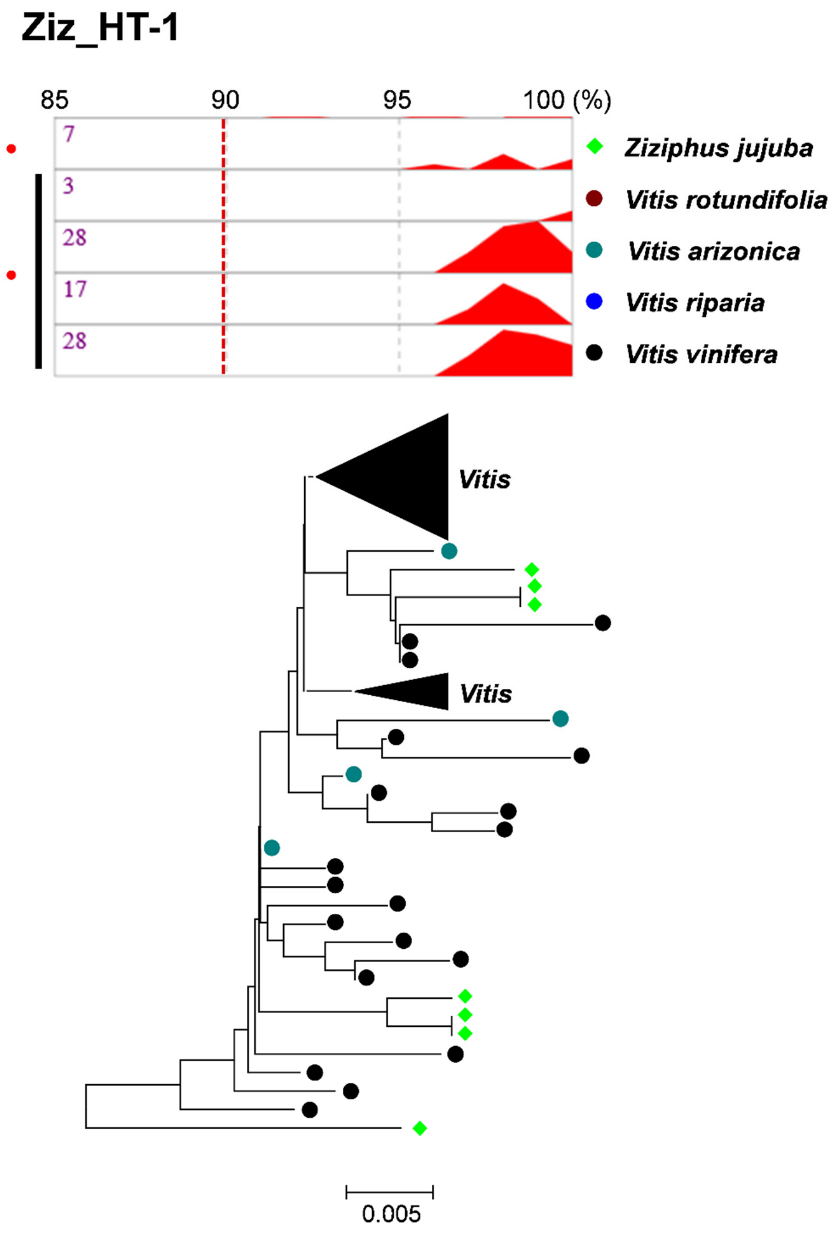

Supplementary Figure S4. Activity history and phylogenetic tree analyses of the horizontally transferred TEs and their homologs. The activity history is shown in the upper panel. The name of the genus or species is shown on the right side of the panel. Each of the *Vitis* species is marked with a colored circle and other non-*Vitis* genera are with colored rhombuses. The percent identity is shown at the top of the panel. The red graph in each square represents the relative activity history of the paralogs of the horizontally transferred TE. The numbers in the panel indicate the number of the used paralogs in each species. The species of the same genus are marked with black bars on the left side of the panel. The red dots indicate the species or genera for which the donor or recipient was undetermined. The red dotted lines present the 90% identity threshold used in the identification of HTs. The lower panel presents the phylogenetic tree of the homologs of the horizontally transferred TE. The species or genus of each element is marked with the same colored circle or rhombus used in the activity history panel. The

branches of recipients are marked with red lines. The triangles represent the merged clusters of the same genus or species. The triangles of the species or genera, except for *Vitis*, are presented in the same color used in the activity history panel. The *Vitis* genus is presented with a black triangle.

Supplementary Figure S5

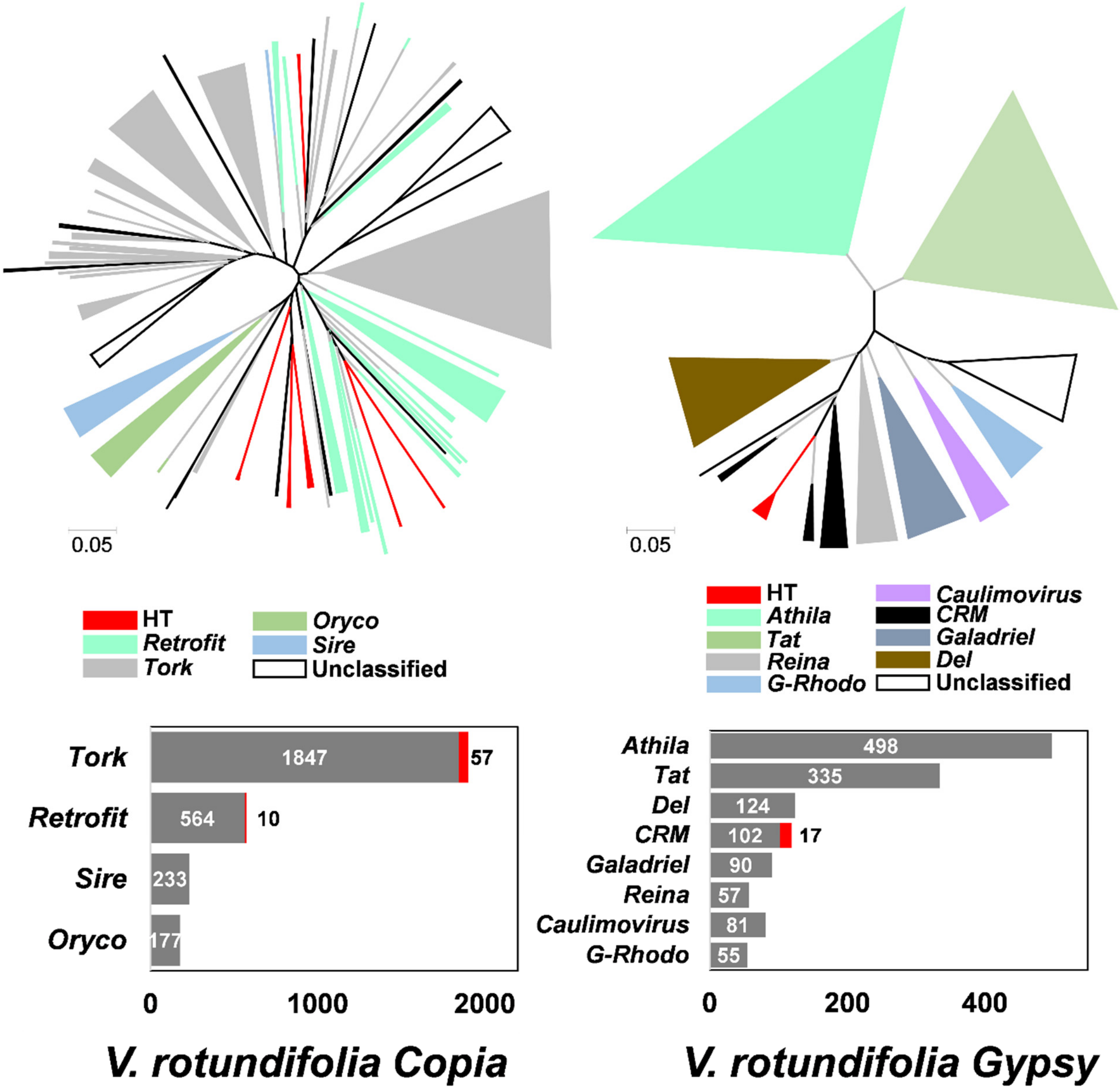

Supplementary Figure S5. *Cont.*

Supplementary Figure S5

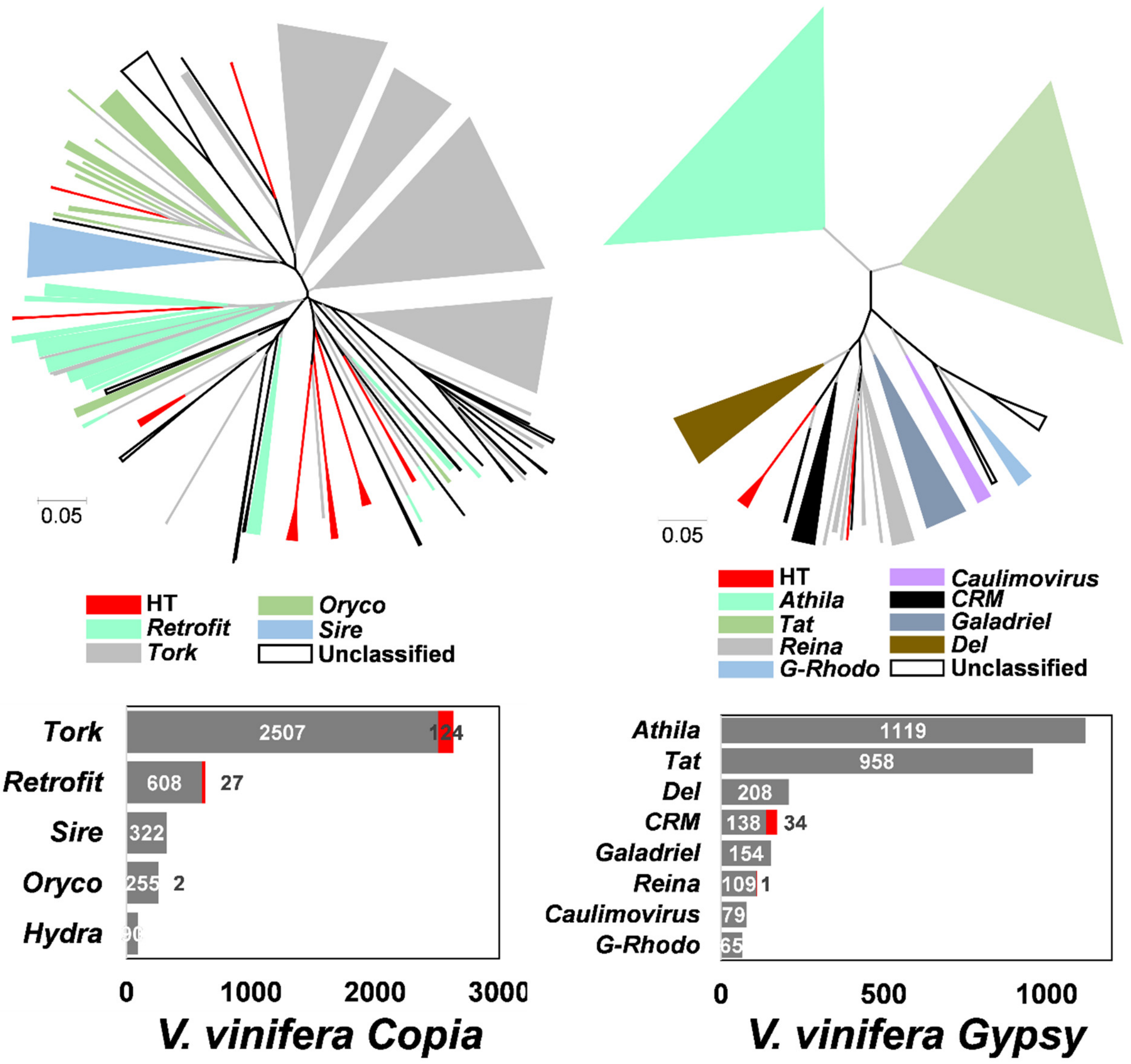

Supplementary Figure S5. *Cont.*

Supplementary Figure S5

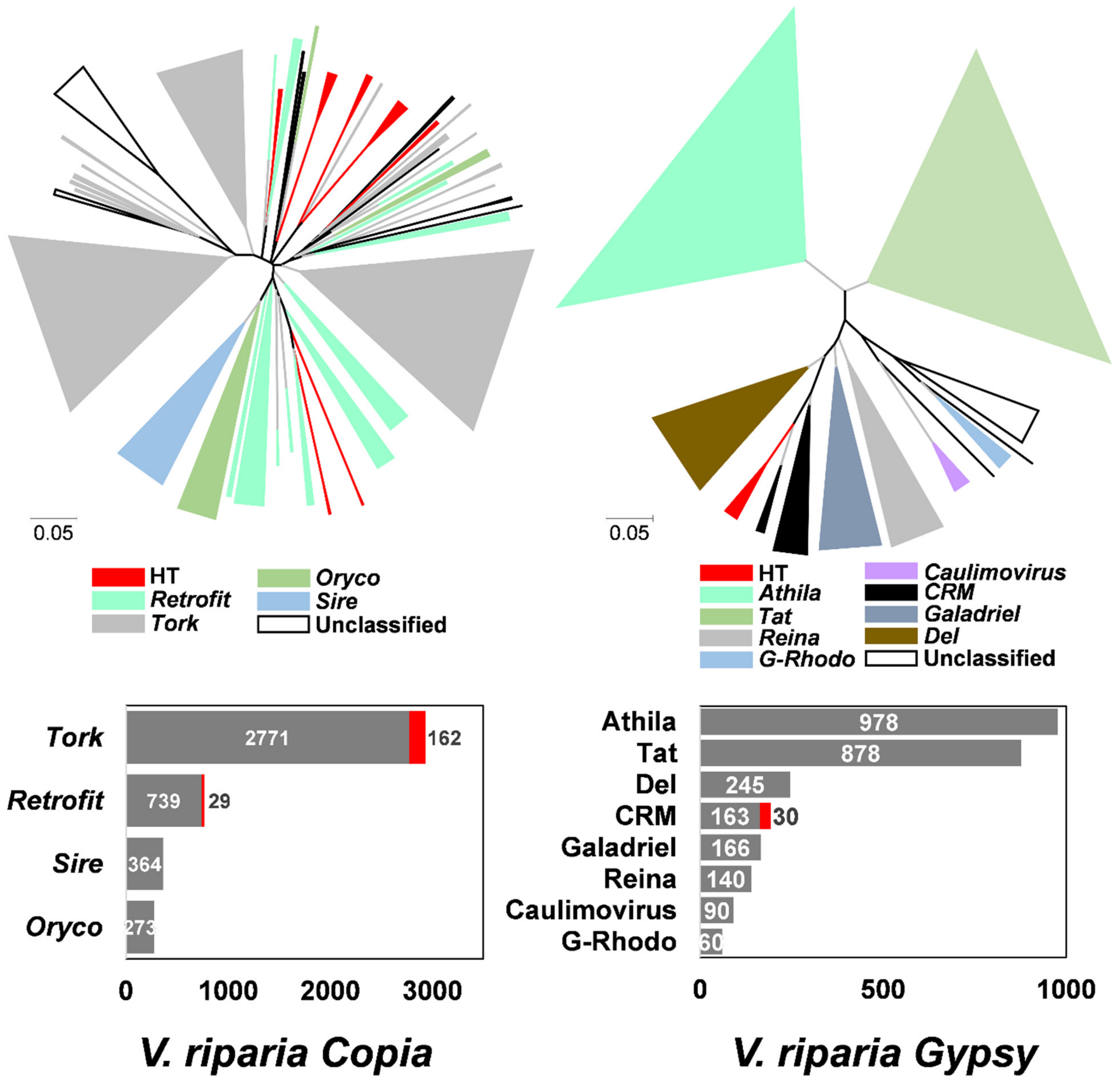

Supplementary Figure S5. *Cont.*

Supplementary Figure S5

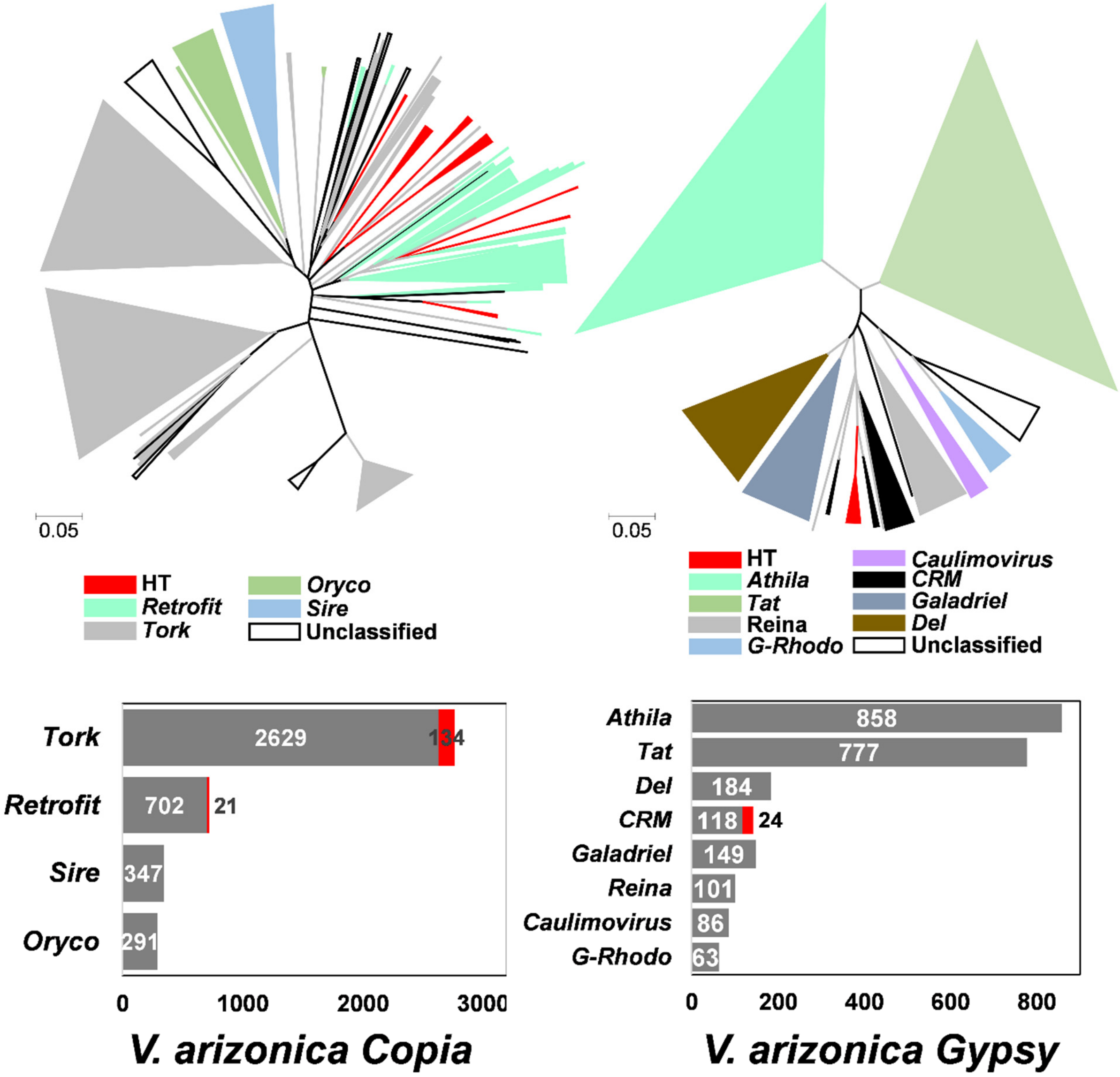

Supplementary Figure S5. Composition of the horizontally transferred LTR retrotransposons in the four Vitis genomes. The phylogenetic trees and composition of the horizontally transferred elements among native *Copia* and *Gypsy* elements in the four *Vitis* species are presented. In the phylogenetic trees, the clusters are represented with triangles and the classified families are presented with different colors. The unclassified clusters are presented with empty triangles. The clusters for the horizontally transferred LTR retrotransposons are marked with red triangles. The composition of the horizontally transferred LTR retrotransposons is shown in the lower panel. The size of LTR retrotransposons (excluding horizontally transferred elements) in each family and the part of horizontally transferred elements are shown with gray and red bar graphs, respectively, and the values are shown with the numbers. The species names and the names of the LTR retrotransposon families are presented on the left side of the panel.

Supplementary Figure S6

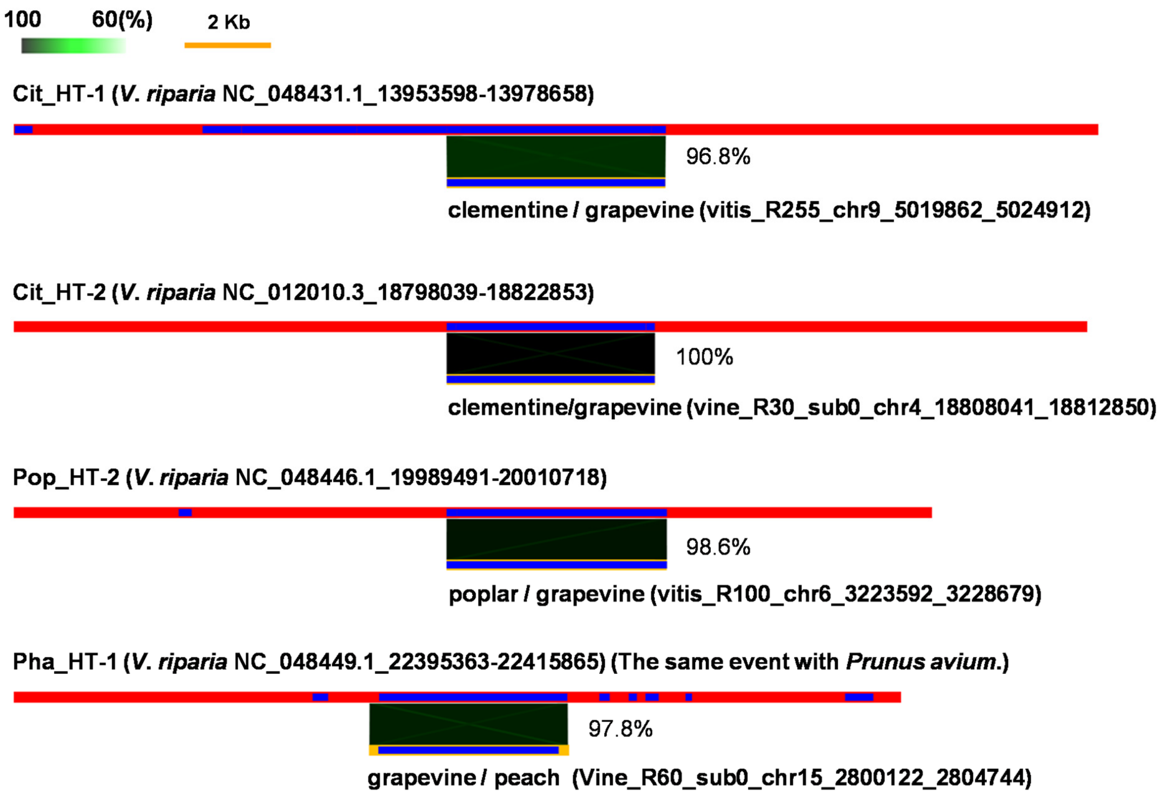

Supplementary Figure S6. Comparative sequence analysis of the four HT events with the previously identified HT events. The *Vitis* sequence contigs containing the horizontally transferred LTR retrotransposons (red bars) are compared to the sequences of the horizontally transferred LTR retrotransposons (yellow bars) identified in the previous study by El Baidouri et al.. The blue bars indicate annotated TEs. The matched sequence regions are linked with green boxes, and the color intensity is proportional to the identity.

Supplementary Figure S7

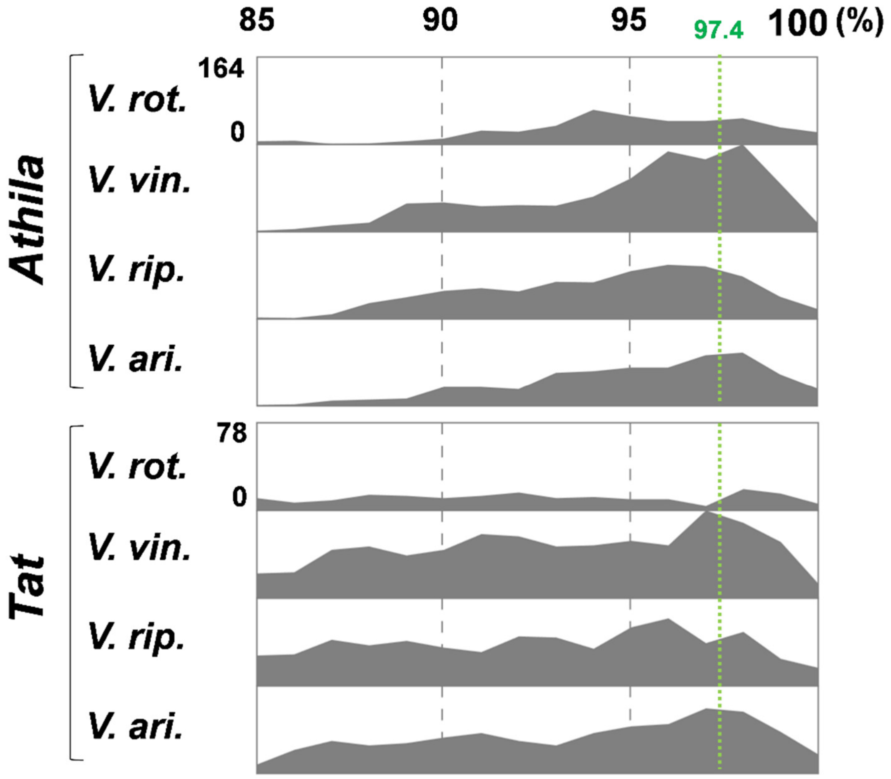

Supplementary Figure S7. Activity history of the two major *Gypsy* families. The relative activity history of the two *Gypsy* families, *Athila* and *Tat*, is presented. The activity history for the two families is shown with gray graphs. The range of the X-axis is shown at the top of the panel. The estimated speciation point of *V. rotundifolia* is indicated with green dotted lines and its identity value is shown at the top of the line. The range of the Y-axis is shown on the left side of each panel and the range is the same as in the other three *Vitis* species. The abbreviations are the same as in Figure 5.
