# Supplementary material for: Horizontal Transfer of LTR Retrotransposons Contributes to the Genome Diversity of Vitis
Source: Int J Mol Sci. 2021 Sep 28;22(19):10446. doi: 10.3390/ijms221910446 (PMC8508631; doi:10.3390/ijms221910446)
Supplement: Supplementary file 1 [file ijms-22-10446-s001.zip › Supplementary_Table_S1.pdf]

Supplementary Table S1. List of plant species used for horizontal transfer investigation.

Species	Family	Citation
Abrus precatorius	Fabaceae	[1]
Aegilops tauschii	Poaceae	[2]
Amborella trichopoda	Amborellaceae	[3]
Ananas comosus	Bromeliaceae	[4]
Arabidopsis lyrata	Brassicaceae	[5]
Arabidopsis thaliana	Brassicaceae	[6]
Arachis duranensis	Fabaceae	[7]
Arachis hypogaea	Fabaceae	[8]
Arachis ipaensis	Fabaceae	[7]
Asparagus officinalis	Asparagaceae	[9]
Beta vulgaris	Chenopodiaceae	[10]
Brachypodium distachyon	Poaceae	[11]
Brassica napus	Brassicaceae	[12]
Brassica oleracea	Brassicaceae	[13]
Brassica rapa	Brassicaceae	[14]
Cajanus cajan	Fabaceae	[15]
Camelina sativa	Brassicaceae	[16]
Camellia sinensis	Theaceae	[17]
Cannabis sativa	Cannabaceae	[18]
Capsella rubella	Brassicaceae	[19]
Capsicum annuum	Solanaceae	[20]
Carica papaya	Caricaceae	[21]
Cerasus x yedoensis	Rosaceae	[22]
Chenopodium quinoa	Amaranthaceae	[23]
Cicer arietinum	Fabaceae	[24]
Citrus clementina	Rutaceae	[25]
Citrus ichangensis	Rutaceae	[26]
Citrus maxima	Rutaceae	[26]
Citrus medica	Rutaceae	[26]
Citrus sinensis	Rutaceae	[27]
Coffea arabica	Rubiaceae	[28]
Coffea eugenioides	Rubiaceae	[28]
Cucumis melo	Cucurbitaceae	[29]
Cucumis sativus	Cucurbitaceae	[30]
Cucurbita maxima	Cucurbitaceae	[31]
Cucurbita moschata	Cucurbitaceae	[31]
Cucurbita pepo	Cucurbitaceae	[32]
Cynara cardunculus	Asteraceae	[33]
Daucus carota	Apiaceae	[34]
Dendrobium catenatum	Orchidaceae	[35]

Dendrobium officinale	Orchidaceae	[36]
Durio zibethinus	Malvaceae	[37]
Elaeis guineensis	Arecaceae	[38]
Erythranthe guttata	Phrymaceae	N/A
Eucalyptus grandis	Myrtaceae	[39]
Eutrema salsugineum	Brassicaceae	[40]
Fragaria iinumae	Rosaceae	[41]
Fragaria nilgerrensis	Rosaceae	[42]
Fragaria nipponica	Rosaceae	[41]
Fragaria nubicola	Rosaceae	[41]
Fragaria orientalis	Rosaceae	[41]
Fragaria vesca	Rosaceae	[43]
Fragaria x ananassa	Rosaceae	[41]
Glycine max	Fabaceae	[44]
Glycine soja	Fabaceae	[45]
Gossypium arboreum	Malvaceae	[46]
Gossypium hirsutum	Malvaceae	[47]
Gossypium raimondi	Malvaceae	[48]
Helianthus annuus	Asteraceae	[49]
Herrania umbratica	Malvaceae	N/A
Hevea brasiliensis	Euphorbiaceae	[50]
Ipomoea nil	Convolvulaceae	[51]
Ipomoea triloba	Convolvulaceae	[52]
Jatropha curcas	Euphorbiaceae	[53]
Juglans regia	Juglandaceae	[54]
Lactuca sativa	Asteraceae	[55]
Lupinus angustifolius	Fabaceae	[56]
Malus baccata	Rosaceae	[57]
Malus domestica	Rosaceae	[58]
Manihot esculenta	Euphorbiaceae	[59]
Medicago truncatula	Fabaceae	[60]
Momordica charantia	Cucurbitaceae	[61]
Morus notabilis	Moraceae	[62]
Musa acuminata	Musaceae	[63]
Muscadinia rotundifolia	Vitaceae	[64]
Nelumbo nucifera	Nelumbonaceae	[63]
Nicotiana attenuata	Solanaceae	[65]
Nicotiana sylvestris	Solanaceae	[66]
Nicotiana tabacum	Solanaceae	[67]
Nicotiana tomentosiformis	Solanaceae	[66]
Nymphaea colorata	Nymphaeaceae	[68]
Olea europaea	Oleaceae	[69]
Oryza brachyantha	Poaceae	[70]

Oryza sativa	Poaceae	[71]
Panicum hallii	Poaceae	[72]
Papaver somniferum	Papaveraceae	[73]
Phalaenopsis equestris	Orchidaceae	[74]
Phaseolus vulgaris	Fabaceae	[75]
Phoenix dactylifera	Arecaceae	[76]
Pistacia vera	Anacardiaceae	[77]
Populus alba	Salicaceae	[78]
Populus euphratica	Salicaceae	[79]
Populus tremula	Salicaceae	[80]
Populus trichocarpa	Salicaceae	[81]
Potentilla micrantha	Rosaceae	[82]
Prosopis alba	Fabaceae	[83]
Prunus armeniaca	Rosaceae	[84]
Prunus avium	Rosaceae	[85]
Prunus domestica	Rosaceae	[86]
Prunus dulcis	Rosaceae	[87]
Prunus ferganensis	Rosaceae	N/A
Prunus mira	Rosaceae	N/A
Prunus mume	Rosaceae	[88]
Prunus persica	Rosaceae	[89]
Prunus salicina	Rosaceae	[90]
Prunus yedoensis	Rosaceae	[91]
Punica granatum	Lythraceae	[92]
Pyrus betulifolia	Rosaceae	[93]
Pyrus bretschneideri	Rosaceae	[94]
Pyrus Communis	Rosaceae	[95]
Pyrys ussuriensis	Rosaceae	[96]
Quercus lobata	Fagaceae	[97]
Quercus suber	Fagaceae	[98]
Raphanus sativus	Brassicaceae	[99]
Rhodamnia argentea	Myrtaceae	N/A
Ricinus communis	Euphorbiaceae	[100]
Rosa chinensis	Rosaceae	[101]
Rosa multiflora	Rosaceae	[102]
Rubus occidentalis	Rosaceae	[103]
Salix viminalis	Salicaceae	[104]
Salix suchowensis	Salicaceae	[105]
Sesamum indicum	Pedaliaceae	[106]
Setaria italica	Poaceae	[107]
Setaria viridis	Poaceae	[108]
Solanum lycopersicum	Solanaceae	[109]
Solanum pennellii	Solanaceae	[110]

Solanum tuberosum	Solanaceae	[111]
Sorghum bicolor	Poaceae	[112]
Spinacia oleracea	Amaranthaceae	[113]
Syzygium oleosum	Myrtaceae	N/A
Tarenaya hassleriana	Cleomaceae	[114]
Theobroma cacao	Malvaceae	[115]
Triticum urartu	Poaceae	[116]
Vigna angularis	Fabaceae	[117]
Vigna radiata	Fabaceae	[118]
Vigna unguiculata	Fabaceae	[119]
Vitis arizonica	Vitaceae	[120]
Vitis riparia	Vitaceae	[121]
Vitis vinifera	Vitaceae	[122]
Zea mays	Poaceae	[123]
Ziziphus jujuba	Rhamnaceae	[124]

References

1. Hovde, B.; Daligault, H.; Hanschen, E.; Kunde, Y.; Johnson, M.; Starkenburg, S.; Johnson, S. Detection of abrin-like and prepropulchellin-like toxin genes and transcripts using whole genome sequencing and full-length transcript sequencing of *Abrus precatorius*. *Toxins* **2019**, *11*, 691.
2. Jia, J.; Zhao, S.; Kong, X.; Li, Y.; Zhao, G.; He, W.; Appels, R.; Pfeifer, M.; Tao, Y.; Zhang, X., *et al.* *Aegilops tauschii* draft genome sequence reveals a gene repertoire for wheat adaptation. *Nature* **2013**, *496*, 91.
3. Amborella Genome Project. The *Amborella* genome and the evolution of flowering plants. *Science* **2013**, *342*, 1241089.
4. Ming, R.; VanBuren, R.; Wai, C.M.; Tang, H.; Schatz, M.C.; Bowers, J.E.; Lyons, E.; Wang, M.-L.; Chen, J.; Biggers, E., *et al.* The pineapple genome and the evolution of cam photosynthesis. *Nat. Genet.* **2015**, *47*, 1435-1442.
5. Hu, T.T.; Pattyn, P.; Bakker, E.G.; Cao, J.; Cheng, J.-F.; Clark, R.M.; Fahlgren, N.; Fawcett, J.A.; Grimwood, J.; Gundlach, H., *et al.* The *Arabidopsis lyrata* genome sequence and the basis of rapid genome size change. *Nat. genet.* **2011**, *43*, 476-481.
6. The Arabidopsis Genome Initiative. Analysis of the genome sequence of the flowering plant *Arabidopsis thaliana*. *Nature* **2000**, *408*, 796.
7. Bertioli, D.J.; Cannon, S.B.; Froenicke, L.; Huang, G.; Farmer, A.D.; Cannon, E.K.S.; Liu, X.; Gao, D.; Clevenger, J.; Dash, S., *et al.* The genome sequences of *Arachis duranensis* and *Arachis ipaensis*, the diploid ancestors of cultivated peanut. *Nat. Genet.* **2016**, *48*, 438-446.
8. Bertioli, D.J.; Jenkins, J.; Clevenger, J.; Dudchenko, O.; Gao, D.; Seijo, G.; Leal-Bertioli, S.C.M.; Ren, L.; Farmer, A.D.; Pandey, M.K., *et al.* The genome sequence of segmental allotetraploid peanut *Arachis hypogaea*. *Nat. Genet.* **2019**, *51*, 877-884.
9. Harkess, A.; Zhou, J.; Xu, C.; Bowers, J.E.; Van der Hulst, R.; Ayyampalayam, S.; Mercati, F.; Riccardi, P.; McKain, M.R.; Kakrana, A., *et al.* The asparagus genome sheds light on the origin and evolution of a young Y chromosome. *Nat. Commun.* **2017**, *8*, 1279.
10. Dohm, J.C.; Minoche, A.E.; Holtgräwe, D.; Capella-Gutiérrez, S.; Zakrzewski, F.; Tafer, H.; Rupp, O.; Sörensen, T.R.; Stracke, R.; Reinhardt, R., *et al.* The genome of the recently domesticated crop plant sugar beet (*Beta vulgaris*). *Nature* **2013**, *505*, 546.
11. The International Brachypodium Initiative. Genome sequencing and analysis of the model grass *Brachypodium distachyon*. *Nature* **2010**, *463*, 763.
12. Chalhoub, B.; Denoeud, F.; Liu, S.; Parkin, I.A.P.; Tang, H.; Wang, X.; Chiquet, J.; Belcram, H.; Tong, C.; Samans, B., *et al.* Early allopolyploid evolution in the post-neolithic *Brassica napus* oilseed genome. *Science* **2014**, *345*, 950-953.
13. Liu, S.; Liu, Y.; Yang, X.; Tong, C.; Edwards, D.; Parkin, I.A.P.; Zhao, M.; Ma, J.; Yu, J.; Huang, S., *et al.* The *Brassica oleracea* genome reveals the asymmetrical evolution of polyploid genomes. *Nat. Commun.* **2014**, *5*, 3930.
14. Wang, X.; Wang, H.; Wang, J.; Sun, R.; Wu, J.; Liu, S.; Bai, Y.; Mun, J.-H.; Bancroft, I.; Cheng, F., *et al.* The genome of the mesopolyploid crop species *Brassica rapa*. *Nat. Genet.* **2011**, *43*, 1035-1039.
15. Varshney, R.K.; Chen, W.; Li, Y.; Bharti, A.K.; Saxena, R.K.; Schlueter, J.A.; Donoghue, M.T.A.; Azam, S.; Fan, G.; Whaley, A.M., *et al.* Draft genome sequence of pigeonpea (*Cajanus cajan*), an orphan legume crop of resource-poor farmers. *Nat. Biotechnol.* **2012**, *30*, 83-89.
16. Chaudhary, R.; Koh, C.S.; Kagale, S.; Tang, L.; Wu, S.W.; Lv, Z.; Mason, A.S.; Sharpe, A.G.; Diederichsen, A.; Parkin, I.A.P. Assessing diversity in the *Camelina* genus provides insights into the genome structure of *Camelina sativa*. *G3 (Bethesda)* **2020**, *10*, 1297.

17. Wei, C.; Yang, H.; Wang, S.; Zhao, J.; Liu, C.; Gao, L.; Xia, E.; Lu, Y.; Tai, Y.; She, G., *et al.* Draft genome sequence of *Camellia sinensis* var. *Sinensis* provides insights into the evolution of the tea genome and tea quality. *Proc. Natl. Acad. Sci. USA* **2018**, *115*, E4151.
18. van Bakel, H.; Stout, J.M.; Cote, A.G.; Tallon, C.M.; Sharpe, A.G.; Hughes, T.R.; Page, J.E. The draft genome and transcriptome of *Cannabis sativa*. *Genome Biol.* **2011**, *12*, R102-R102.
19. Slotte, T.; Hazzouri, K.M.; Ågren, J.A.; Koenig, D.; Maumus, F.; Guo, Y.-L.; Steige, K.; Platts, A.E.; Escobar, J.S.; Newman, L.K., *et al.* The *Capsella rubella* genome and the genomic consequences of rapid mating system evolution. *Nat. Genet.* **2013**, *45*, 831.
20. Kim, S.; Park, M.; Yeom, S.-I.; Kim, Y.-M.; Lee, J.M.; Lee, H.-A.; Seo, E.; Choi, J.; Cheong, K.; Kim, K.-T., *et al.* Genome sequence of the hot pepper provides insights into the evolution of pungency in *Capsicum* species. *Nat. Genet.* **2014**, *46*, 270.
21. Ming, R.; Hou, S.; Feng, Y.; Yu, Q.; Dionne-Laporte, A.; Saw, J.H.; Senin, P.; Wang, W.; Ly, B.V.; Lewis, K.L.T., *et al.* The draft genome of the transgenic tropical fruit tree papaya (*Carica papaya* Linnaeus). *Nature* **2008**, *452*, 991-996.
22. Shirasawa, K.; Esumi, T.; Hirakawa, H.; Tanaka, H.; Itai, A.; Ghelfi, A.; Nagasaki, H.; Isobe, S. Phased genome sequence of an interspecific hybrid flowering cherry, 'Somei-Yoshino' (*Cerasus* × *yedoensis*). *DNA Res.* **2019**, *26*, 379-389.
23. Jarvis, D.E.; Ho, Y.S.; Lightfoot, D.J.; Schmöckel, S.M.; Li, B.; Borm, T.J.A.; Ohyanagi, H.; Mineta, K.; Michell, C.T.; Saber, N., *et al.* The genome of *Chenopodium quinoa*. *Nature* **2017**, *542*, 307-312.
24. Varshney, R.K.; Song, C.; Saxena, R.K.; Azam, S.; Yu, S.; Sharpe, A.G.; Cannon, S.; Baek, J.; Rosen, B.D.; Tar'an, B., *et al.* Draft genome sequence of chickpea (*Cicer arietinum*) provides a resource for trait improvement. *Nat. Biotechnol.* **2013**, *31*, 240.
25. Wu, G.A.; Prochnik, S.; Jenkins, J.; Salse, J.; Hellsten, U.; Murat, F.; Perrier, X.; Ruiz, M.; Scalabrin, S.; Terol, J., *et al.* Sequencing of diverse mandarin, pummelo and orange genomes reveals complex history of admixture during citrus domestication. *Nat. Biotechnol.* **2014**, *32*, 656-662.
26. Wang, X.; Xu, Y.; Zhang, S.; Cao, L.; Huang, Y.; Cheng, J.; Wu, G.; Tian, S.; Chen, C.; Liu, Y., *et al.* Genomic analyses of primitive, wild and cultivated citrus provide insights into asexual reproduction. *Nature Genetics* **2017**, *49*, 765-772.
27. Xu, Q.; Chen, L.-L.; Ruan, X.; Chen, D.; Zhu, A.; Chen, C.; Bertrand, D.; Jiao, W.-B.; Hao, B.-H.; Lyon, M.P., *et al.* The draft genome of sweet orange (*Citrus sinensis*). *Nat. Genet.* **2012**, *45*, 59.
28. Scalabrin, S.; Toniutti, L.; Di Gaspero, G.; Scaglione, D.; Magris, G.; Vidotto, M.; Pinosio, S.; Cattonaro, F.; Magni, F.; Jurman, I., *et al.* A single polyploidization event at the origin of the tetraploid genome of *Coffea arabica* is responsible for the extremely low genetic variation in wild and cultivated germplasm. *Sci. Rep.* **2020**, *10*, 4642.
29. Garcia-Mas, J.; Benjak, A.; Sanseverino, W.; Bourgeois, M.; Mir, G.; González, V.M.; Hénaff, E.; Câmara, F.; Cozzuto, L.; Lowy, E., *et al.* The genome of melon (*Cucumis melo*l.). *Proc. Natl. Acad. Sci. USA* **2012**, *109*, 11872-11877.
30. Huang, S.; Li, R.; Zhang, Z.; Li, L.; Gu, X.; Fan, W.; Lucas, W.J.; Wang, X.; Xie, B.; Ni, P., *et al.* The genome of the cucumber, *Cucumis sativus* L. *Nat. Genet.* **2009**, *41*, 1275-1281.
31. Sun, H.; Wu, S.; Zhang, G.; Jiao, C.; Guo, S.; Ren, Y.; Zhang, J.; Zhang, H.; Gong, G.; Jia, Z., *et al.* Karyotype stability and unbiased fractionation in the paleo-allotetraploid *Cucurbita* genomes. *Mol. Plant* **2017**, *10*, 1293-1306.
32. Xanthopoulou, A.; Montero-Pau, J.; Mellidou, I.; Kissoudis, C.; Blanca, J.; Picó, B.; Tsaballa, A.; Tsaliki, E.; Dalakouras, A.; Paris, H.S., *et al.* Whole-genome resequencing of *Cucurbita pepo* morphotypes to discover genomic variants associated with morphology and horticulturally valuable traits. *Hortic. Res.* **2019**, *6*, 94.
33. Scaglione, D.; Reyes-Chin-Wo, S.; Acquadro, A.; Froenicke, L.; Portis, E.; Beitel, C.; Tirone, M.; Mauro, R.; Lo Monaco, A.; Mauromicale, G., *et al.* The genome sequence of the outbreeding globe

artichoke constructed de novo incorporating a phase-aware low-pass sequencing strategy of F1 progeny. *Sci. Rep.* **2016**, *6*, 19427.
34. Iorizzo, M.; Ellison, S.; Senalik, D.; Zeng, P.; Satapoomin, P.; Huang, J.; Bowman, M.; Iovene, M.; Sanseverino, W.; Cavagnaro, P., *et al.* A high-quality carrot genome assembly provides new insights into carotenoid accumulation and asterid genome evolution. *Nat. Genet.* **2016**, *48*, 657-666.
35. Zhang, G.-Q.; Xu, Q.; Bian, C.; Tsai, W.-C.; Yeh, C.-M.; Liu, K.-W.; Yoshida, K.; Zhang, L.-S.; Chang, S.-B.; Chen, F., *et al.* The *Dendrobium catenatum* Lindl. Genome sequence provides insights into polysaccharide synthase, floral development and adaptive evolution. *Sci. Rep.* **2016**, *6*, 19029.
36. Yan, L.; Wang, X.; Liu, H.; Tian, Y.; Lian, J.; Yang, R.; Hao, S.; Wang, X.; Yang, S.; Li, Q., *et al.* The genome of *Dendrobium officinale* illuminates the biology of the important traditional Chinese orchid herb. *Mol. Plant* **2015**, *8*, 922-934.
37. Teh, B.T.; Lim, K.; Yong, C.H.; Ng, C.C.Y.; Rao, S.R.; Rajasegaran, V.; Lim, W.K.; Ong, C.K.; Chan, K.; Cheng, V.K.Y., *et al.* The draft genome of tropical fruit durian (*Durio zibethinus*). *Nat. Genet.* **2017**, *49*, 1633-1641.
38. Singh, R.; Ong-Abdullah, M.; Low, E.-T.L.; Manaf, M.A.A.; Rosli, R.; Nookiah, R.; Ooi, L.C.-L.; Ooi, S.E.; Chan, K.-L.; Halim, M.A., *et al.* Oil palm genome sequence reveals divergence of interfertile species in Old and New worlds. *Nature* **2013**, *500*, 335.
39. Myburg, A.A.; Grattapaglia, D.; Tuskan, G.A.; Hellsten, U.; Hayes, R.D.; Grimwood, J.; Jenkins, J.; Lindquist, E.; Tice, H.; Bauer, D., *et al.* The genome of *Eucalyptus grandis*. *Nature* **2014**, *510*, 356.
40. Yang, R.; Jarvis, D.E.; Chen, H.; Beilstein, M.A.; Grimwood, J.; Jenkins, J.; Shu, S.; Prochnik, S.; Xin, M.; Ma, C., *et al.* The reference genome of the halophytic plant *Eutrema salsugineum*. *Front. Plant. Sci.* **2013**, *4*, 46.
41. Hirakawa, H.; Shirasawa, K.; Kosugi, S.; Tashiro, K.; Nakayama, S.; Yamada, M.; Kohara, M.; Watanabe, A.; Kishida, Y.; Fujishiro, T., *et al.* Dissection of the octoploid strawberry genome by deep sequencing of the genomes of *Fragaria* species. *DNA Res.* **2014**, *21*, 169-181.
42. Zhang, J.; Lei, Y.; Wang, B.; Li, S.; Yu, S.; Wang, Y.; Li, H.; Liu, Y.; Ma, Y.; Dai, H., *et al.* The high-quality genome of diploid strawberry (*Fragaria nilgerrensis*) provides new insights into anthocyanin accumulation. *Plant Biotechnol. J.* **2020**, *18*, 1908-1924.
43. Shulaev, V.; Sargent, D.J.; Crowhurst, R.N.; Mockler, T.C.; Folkerts, O.; Delcher, A.L.; Jaiswal, P.; Mockaitis, K.; Liston, A.; Mane, S.P., *et al.* The genome of woodland strawberry (*Fragaria vesca*). *Nat. Genet.* **2011**, *43*, 109-116.
44. Schmutz, J.; Cannon, S.B.; Schlueter, J.; Ma, J.; Mitros, T.; Nelson, W.; Hyten, D.L.; Song, Q.; Thelen, J.J.; Cheng, J., *et al.* Genome sequence of the palaeopolyploid soybean. *Nature* **2010**, *463*, 178.
45. Kim, M.Y.; Lee, S.; Van, K.; Kim, T.-H.; Jeong, S.-C.; Choi, I.-Y.; Kim, D.-S.; Lee, Y.-S.; Park, D.; Ma, J., *et al.* Whole-genome sequencing and intensive analysis of the undomesticated soybean (*Glycine soja* Sieb. and Zucc.) genome. *Proc. Natl. Acad. Sci. USA* **2010**, *107*, 22032.
46. Li, F.; Fan, G.; Wang, K.; Sun, F.; Yuan, Y.; Song, G.; Li, Q.; Ma, Z.; Lu, C.; Zou, C., *et al.* Genome sequence of the cultivated cotton *Gossypium arboreum*. *Nat. Genet.* **2014**, *46*, 567-572.
47. Li, F.; Fan, G.; Lu, C.; Xiao, G.; Zou, C.; Kohel, R.J.; Ma, Z.; Shang, H.; Ma, X.; Wu, J., *et al.* Genome sequence of cultivated upland cotton (*Gossypium hirsutum* TM-1) provides insights into genome evolution. *Nat. Biotechnol.* **2015**, *33*, 524-530.
48. Wang, K.; Wang, Z.; Li, F.; Ye, W.; Wang, J.; Song, G.; Yue, Z.; Cong, L.; Shang, H.; Zhu, S., *et al.* The draft genome of a diploid cotton *Gossypium raimondii*. *Nat. Genet.* **2012**, *44*, 1098.
49. Badouin, H.; Gouzy, J.; Grassa, C.J.; Murat, F.; Staton, S.E.; Cottret, L.; Lelandais-Brière, C.; Owens, G.L.; Carrère, S.; Mayjonade, B., *et al.* The sunflower genome provides insights into oil metabolism, flowering and Asterid evolution. *Nature* **2017**, *546*, 148-152.

50. Rahman, A.Y.A.; Usharraj, A.O.; Misra, B.B.; Thottathil, G.P.; Jayasekaran, K.; Feng, Y.; Hou, S.; Ong, S.Y.; Ng, F.L.; Lee, L.S., *et al.* Draft genome sequence of the rubber tree *Hevea brasiliensis*. *BMC Genomics* **2013**, *14*, 75.
51. Hoshino, A.; Jayakumar, V.; Nitasaka, E.; Toyoda, A.; Noguchi, H.; Itoh, T.; Shin-I, T.; Minakuchi, Y.; Koda, Y.; Nagano, A.J., *et al.* Genome sequence and analysis of the japanese morning glory *Ipomoea nil*. *Nat. Commun.* **2016**, *7*, 13295.
52. Wu, S.; Lau, K.H.; Cao, Q.; Hamilton, J.P.; Sun, H.; Zhou, C.; Eserman, L.; Gemenet, D.C.; Olukolu, B.A.; Wang, H., *et al.* Genome sequences of two diploid wild relatives of cultivated sweetpotato reveal targets for genetic improvement. *Nat. Commun.* **2018**, *9*, 4580.
53. Sato, S.; Hirakawa, H.; Isobe, S.; Fukai, E.; Watanabe, A.; Kato, M.; Kawashima, K.; Minami, C.; Muraki, A.; Nakazaki, N., *et al.* Sequence analysis of the genome of an oil-bearing tree, *Jatropha curcas* l. *DNA Res.* **2011**, *18*, 65-76.
54. Martínez-García, P.J.; Crepeau, M.W.; Puiu, D.; Gonzalez-Ibeas, D.; Whalen, J.; Stevens, K.A.; Paul, R.; Butterfield, T.S.; Britton, M.T.; Reagan, R.L., *et al.* The walnut (*Juglans regia*) genome sequence reveals diversity in genes coding for the biosynthesis of non-structural polyphenols. *Plant J.* **2016**, *87*, 507-532.
55. Verwaaijen, B.; Wibberg, D.; Nelkner, J.; Gordin, M.; Rupp, O.; Winkler, A.; Bremges, A.; Blom, J.; Grosch, R.; Pühler, A., *et al.* Assembly of the *Lactuca sativa*, l. cv. Tizian draft genome sequence reveals differences within major resistance complex 1 as compared to the cv. Salinas reference genome. *J. Biotechnol.* **2018**, *267*, 12-18.
56. Hane, J.K.; Ming, Y.; Kamphuis, L.G.; Nelson, M.N.; Garg, G.; Atkins, C.A.; Bayer, P.E.; Bravo, A.; Bringans, S.; Cannon, S., *et al.* A comprehensive draft genome sequence for lupin (*Lupinus angustifolius*), an emerging health food: Insights into plant–microbe interactions and legume evolution. *Plant Biotechnol. J.* **2017**, *15*, 318-330.
57. Chen, X.; Li, S.; Zhang, D.; Han, M.; Jin, X.; Zhao, C.; Wang, S.; Xing, L.; Ma, J.; Ji, J., *et al.* Sequencing of a wild pple (*Malus baccata*) genome unravels the differences between cultivated and wild apple species regarding disease resistance and cold tolerance. *G3 (Bethesda)* **2019**, *9*, 2051.
58. Velasco, R.; Zharkikh, A.; Affourtit, J.; Dhingra, A.; Cestaro, A.; Kalyanaraman, A.; Fontana, P.; Bhatnagar, S.K.; Troggio, M.; Pruss, D., *et al.* The genome of the domesticated apple (*Malus* × *domestica* Borkh.). *Nat. Genet.* **2010**, *42*, 833.
59. Wang, W.; Feng, B.; Xiao, J.; Xia, Z.; Zhou, X.; Li, P.; Zhang, W.; Wang, Y.; Møller, B.L.; Zhang, P., *et al.* Cassava genome from a wild ancestor to cultivated varieties. *Nat. Commun.* **2014**, *5*, 5110.
60. Young, N.D.; Debellé, F.; Oldroyd, G.E.D.; Geurts, R.; Cannon, S.B.; Udvardi, M.K.; Benedito, V.A.; Mayer, K.F.X.; Gouzy, J.; Schoof, H., *et al.* The medicago genome provides insight into the evolution of rhizobial symbioses. *Nature* **2011**, *480*, 520-524.
61. Urasaki, N.; Takagi, H.; Natsume, S.; Uemura, A.; Taniai, N.; Miyagi, N.; Fukushima, M.; Suzuki, S.; Tarora, K.; Tamaki, M., *et al.* Draft genome sequence of bitter gourd (*Momordica charantia*), a vegetable and medicinal plant in tropical and subtropical regions. *DNA Res.* **2016**, *24*, 51-58.
62. He, N.; Zhang, C.; Qi, X.; Zhao, S.; Tao, Y.; Yang, G.; Lee, T.-H.; Wang, X.; Cai, Q.; Li, D., *et al.* Draft genome sequence of the mulberry tree *Morus notabilis*. *Nat. Commun.* **2013**, *4*, 2445.
63. D'Hont, A.; Denoeud, F.; Aury, J.-M.; Baurens, F.-C.; Carreel, F.; Garsmeur, O.; Noel, B.; Bocs, S.; Droc, G.; Rouard, M., *et al.* The banana (*Musa acuminata*) genome and the evolution of monocotyledonous plants. *Nature* **2012**, *488*, 213.
64. Cochetel, N.; Minio, A.; Massonnet, M.; Vondras, A.M.; Figueroa-Balderas, R.; Cantu, D. Diploid chromosome-scale assembly of the *Muscadinia rotundifolia* genome supports chromosome fusion and disease resistance gene expansion during *Vitis* and *muscadinia* divergence. *G3 (Bethesda)* **2021**, *11*, jkab033.

65. Xu, S.; Brockmöller, T.; Navarro-Quezada, A.; Kuhl, H.; Gase, K.; Ling, Z.; Zhou, W.; Kreitzer, C.; Stanke, M.; Tang, H., *et al.* Wild tobacco genomes reveal the evolution of nicotine biosynthesis. *Proc. Natl. Acad. Sci. USA* **2017**, *114*, 6133.
66. Sierro, N.; Battey, J.N.; Ouadi, S.; Bovet, L.; Goepfert, S.; Bakaher, N.; Peitsch, M.C.; Ivanov, N.V. Reference genomes and transcriptomes of *Nicotiana sylvestris* and *Nicotiana tomentosiformis*. *Genome Biol.* **2013**, *14*, R60.
67. Sierro, N.; Battey, J.N.D.; Ouadi, S.; Bakaher, N.; Bovet, L.; Willig, A.; Goepfert, S.; Peitsch, M.C.; Ivanov, N.V. The tobacco genome sequence and its comparison with those of tomato and potato. *Nat. Commun.* **2014**, *5*, 3833.
68. Zhang, L.; Chen, F.; Zhang, X.; Li, Z.; Zhao, Y.; Lohaus, R.; Chang, X.; Dong, W.; Ho, S.Y.W.; Liu, X., *et al.* The water lily genome and the early evolution of flowering plants. *Nature* **2020**, *577*, 79-84.
69. Cruz, F.; Julca, I.; Gómez-Garrido, J.; Loska, D.; Marcet-Houben, M.; Cano, E.; Galán, B.; Frias, L.; Ribeca, P.; Derdak, S., *et al.* Genome sequence of the olive tree, *Olea europaea*. *GigaScience* **2016**, *5*, 29.
70. Chen, J.; Huang, Q.; Gao, D.; Wang, J.; Lang, Y.; Liu, T.; Li, B.; Bai, Z.; Luis Goicoechea, J.; Liang, C., *et al.* Whole-genome sequencing of *Oryza brachyantha* reveals mechanisms underlying *Oryza* genome evolution. *Nat. Commun.* **2013**, *4*, 1595.
71. Goff, S.A.; Ricke, D.; Lan, T.-H.; Presting, G.; Wang, R.; Dunn, M.; Glazebrook, J.; Sessions, A.; Oeller, P.; Varma, H., *et al.* A draft sequence of the rice genome (*Oryza sativa* L. ssp. *japonica*). *Science* **2002**, *296*, 92-100.
72. Lovell, J.T.; Jenkins, J.; Lowry, D.B.; Mamidi, S.; Sreedasyam, A.; Weng, X.; Barry, K.; Bonnette, J.; Campitelli, B.; Daum, C., *et al.* The genomic landscape of molecular responses to natural drought stress in *Panicum hallii*. *Nat. Commun.* **2018**, *9*, 5213.
73. Guo, L.; Winzer, T.; Yang, X.; Li, Y.; Ning, Z.; He, Z.; Teodor, R.; Lu, Y.; Bowser, T.A.; Graham, I.A., *et al.* The opium poppy genome and morphinan production. *Science* **2018**, *362*, 343.
74. Cai, J.; Liu, X.; Vanneste, K.; Proost, S.; Tsai, W.-C.; Liu, K.-W.; Chen, L.-J.; He, Y.; Xu, Q.; Bian, C., *et al.* The genome sequence of the orchid *Phalaenopsis equestris*. *Nat. Genet.* **2015**, *47*, 65-72.
75. Schmutz, J.; McClean, P.E.; Mamidi, S.; Wu, G.A.; Cannon, S.B.; Grimwood, J.; Jenkins, J.; Shu, S.; Song, Q.; Chavarro, C., *et al.* A reference genome for common bean and genome-wide analysis of dual domestications. *Nat. Genet.* **2014**, *46*, 707.
76. Al-Dous, E.K.; George, B.; Al-Mahmoud, M.E.; Al-Jaber, M.Y.; Wang, H.; Salameh, Y.M.; Al-Azwani, E.K.; Chaluvadi, S.; Pontaroli, A.C.; DeBarry, J., *et al. De novo* genome sequencing and comparative genomics of date palm (*Phoenix dactylifera*). *Nat. Biotechnol.* **2011**, *29*, 521-528.
77. Zeng, L.; Tu, X.-L.; Dai, H.; Han, F.-M.; Lu, B.-S.; Wang, M.-S.; Nanaei, H.A.; Tajabadipour, A.; Mansouri, M.; Li, X.-L., *et al.* Whole genomes and transcriptomes reveal adaptation and domestication of pistachio. *Genome Biol.* **2019**, *20*, 79.
78. Liu, Y.-J.; Wang, X.-R.; Zeng, Q.-Y. *De novo* assembly of white poplar genome and genetic diversity of white poplar population in Irtysh River basin in China. *Sci. China Life Sci.* **2019**, *62*, 609-618.
79. Ma, T.; Wang, J.; Zhou, G.; Yue, Z.; Hu, Q.; Chen, Y.; Liu, B.; Qiu, Q.; Wang, Z.; Zhang, J., *et al.* Genomic insights into salt adaptation in a desert poplar. *Nat. Commun.* **2013**, *4*, 2797.
80. Lin, Y.-C.; Wang, J.; Delhomme, N.; Schiffthaler, B.; Sundström, G.; Zuccolo, A.; Nystedt, B.; Hvidsten, T.R.; de la Torre, A.; Cossu, R.M., *et al.* Functional and evolutionary genomic inferences in *Populus* through genome and population sequencing of American and European aspen. *Proc. Natl. Acad. Sci. USA* **2018**, *115*, E10970.

81. Tuskan, G.A.; DiFazio, S.; Jansson, S.; Bohlmann, J.; Grigoriev, I.; Hellsten, U.; Putnam, N.; Ralph, S.; Rombauts, S.; Salamov, A., *et al.* The genome of black cottonwood, *Populus trichocarpa* (Torr. Gray). *Science* **2006**, *313*, 1596-1604.
82. Buti, M.; Moretto, M.; Barghini, E.; Mascagni, F.; Natali, L.; Brilli, M.; Lomsadze, A.; Sonego, P.; Giongo, L.; Alonge, M., *et al.* The genome sequence and transcriptome of *Potentilla micrantha* and their comparison to *Fragaria vesca* (the woodland strawberry). *GigaScience* **2018**, *7*, 1-14.
83. Torales, S.L.; Rivarola, M.; Pomponio, M.F.; Gonzalez, S.; Acuña, C.V.; Fernández, P.; Lauenstein, D.L.; Verga, A.R.; Hopp, H.E.; Paniego, N.B., *et al. De novo* assembly and characterization of leaf transcriptome for the development of functional molecular markers of the extremophile multipurpose tree species *Prosopis alba*. *BMC Genomics* **2013**, *14*, 705.
84. Jiang, F.; Zhang, J.; Wang, S.; Yang, L.; Luo, Y.; Gao, S.; Zhang, M.; Wu, S.; Hu, S.; Sun, H., *et al.* The apricot (*Prunus armeniaca* l.) genome elucidates Rosaceae evolution and beta-carotenoid synthesis. *Hortic. Res.* **2019**, *6*, 128.
85. Shirasawa, K.; Isuzugawa, K.; Ikenaga, M.; Saito, Y.; Yamamoto, T.; Hirakawa, H.; Isobe, S. The genome sequence of sweet cherry (*Prunus avium*) for use in genomics-assisted breeding. *DNA Res.* **2017**, *24*, 499-508.
86. Zhebentyayeva, T.; Shankar, V.; Scorza, R.; Callahan, A.; Ravelonandro, M.; Castro, S.; DeJong, T.; Saski, C.A.; Dardick, C. Genetic characterization of worldwide *Prunus domestica* (plum) germplasm using sequence-based genotyping. *Hortic. Res.* **2019**, *6*, 12.
87. Alioto, T.; Alexiou, K.G.; Bardil, A.; Barteri, F.; Castanera, R.; Cruz, F.; Dhingra, A.; Duval, H.; Fernández i Martí, Á.; Frias, L., *et al.* Transposons played a major role in the diversification between the closely related almond and peach genomes: Results from the almond genome sequence. *Plant J.* **2020**, *101*, 455-472.
88. Zhang, Q.; Chen, W.; Sun, L.; Zhao, F.; Huang, B.; Yang, W.; Tao, Y.; Wang, J.; Yuan, Z.; Fan, G., *et al.* The genome of *Prunus mume*. *Nat. Commun.* **2012**, *3*, 1318.
89. The International Peach Genome Initiative. The high-quality draft genome of peach (*Prunus persica*) identifies unique patterns of genetic diversity, domestication and genome evolution. *Nat. Genet.* **2013**, *45*, 487.
90. Liu, C.; Feng, C.; Peng, W.; Hao, J.; Wang, J.; Pan, J.; He, Y. Chromosome-level draft genome of a diploid plum (*Prunus salicina*). *GigaScience* **2020**, *9*.
91. Baek, S.; Choi, K.; Kim, G.-B.; Yu, H.-J.; Cho, A.; Jang, H.; Kim, C.; Kim, H.-J.; Chang, K.S.; Kim, J.-H., *et al.* Draft genome sequence of wild *Prunus yedoensis* reveals massive inter-specific hybridization between sympatric flowering cherries. *Genome Biol.* **2018**, *19*, 127.
92. Yuan, Z.; Fang, Y.; Zhang, T.; Fei, Z.; Han, F.; Liu, C.; Liu, M.; Xiao, W.; Zhang, W.; Wu, S., *et al.* The pomegranate (*Punica granatum* l.) genome provides insights into fruit quality and ovule developmental biology. *Plant Biotechnol. J.* **2018**, *16*, 1363-1374.
93. Dong, X.; Wang, Z.; Tian, L.; Zhang, Y.; Qi, D.; Huo, H.; Xu, J.; Li, Z.; Liao, R.; Shi, M., *et al. De novo* assembly of a wild pear (*Pyrus betuleafolia*) genome. *Plant Biotechnol. J.***2020**, *18*, 581-595.
94. Wu, J.; Wang, Z.; Shi, Z.; Zhang, S.; Ming, R.; Zhu, S.; Khan, M.A.; Tao, S.; Korban, S.S.; Wang, H., *et al.* The genome of the pear (*Pyrus bretschneideri* Rehd.). *Genome Res.* **2013**, *23*, 396-408.
95. Chagné, D.; Crowhurst, R.N.; Pindo, M.; Thrimawithana, A.; Deng, C.; Ireland, H.; Fiers, M.; Dzierzon, H.; Cestaro, A.; Fontana, P., *et al.* The draft genome sequence of european pear (*Pyrus communis* L. 'Bartlett'). *PLoS one* **2014**, *9*, e92644.
96. Ou, C.; Wang, F.; Wang, J.; Li, S.; Zhang, Y.; Fang, M.; Ma, L.; Zhao, Y.; Jiang, S. A *de novo* genome assembly of the dwarfing pear rootstock Zhongai 1. *Sci. Data* **2019**, *6*, 281.
97. Sork, V.L.; Fitz-Gibbon, S.T.; Puiu, D.; Crepeau, M.; Gugger, P.F.; Sherman, R.; Stevens, K.; Langley, C.H.; Pellegrini, M.; Salzberg, S.L. First draft assembly and annotation of the genome of a california endemic oak *Quercus lobata* née (Fagaceae). *G3 (Bethesda)* **2016**, *6*, 3485-3495.

98. Ramos, A.M.; Usié, A.; Barbosa, P.; Barros, P.M.; Capote, T.; Chaves, I.; Simões, F.; Abreu, I.; Carrasquinho, I.; Faro, C., *et al.* The draft genome sequence of cork oak. *Sci. Data* **2018**, *5*, 180069.
99. Kitashiba, H.; Li, F.; Hirakawa, H.; Kawanabe, T.; Zou, Z.; Hasegawa, Y.; Tonosaki, K.; Shirasawa, S.; Fukushima, A.; Yokoi, S., *et al.* Draft sequences of the radish (*Raphanus sativus* l.) genome. *DNA Res.* **2014**, *21*, 481-490.
100. Chan, A.P.; Crabtree, J.; Zhao, Q.; Lorenzi, H.; Orvis, J.; Puiu, D.; Melake-Berhan, A.; Jones, K.M.; Redman, J.; Chen, G., *et al.* Draft genome sequence of the oilseed species *Ricinus communis*. *Nat. Biotechnol.* **2010**, *28*, 951.
101. Hibrand Saint-Oyant, L.; Ruttink, T.; Hamama, L.; Kirov, I.; Lakhwani, D.; Zhou, N.N.; Bourke, P.M.; Daccord, N.; Leus, L.; Schulz, D., *et al.* A high-quality genome sequence of *Rosa chinensis* to elucidate ornamental traits. *Nat. Plants* **2018**, *4*, 473-484.
102. Nakamura, N.; Hirakawa, H.; Sato, S.; Otagaki, S.; Matsumoto, S.; Tabata, S.; Tanaka, Y. Genome structure of *Rosa multiflora*, a wild ancestor of cultivated roses. *DNA Res.* **2018**, *25*, 113-121.
103. VanBuren, R.; Wai, C.M.; Colle, M.; Wang, J.; Sullivan, S.; Bushakra, J.M.; Liachko, I.; Vining, K.J.; Dossett, M.; Finn, C.E., *et al.* A near complete, chromosome-scale assembly of the black raspberry (*Rubus occidentalis*) genome. *GigaScience* **2018**, *7*.
104. Almeida, P.; Proux-Wera, E.; Churcher, A.; Soler, L.; Dainat, J.; Pucholt, P.; Nordlund, J.; Martin, T.; Rönnberg-Wästljung, A.-C.; Nystedt, B., *et al.* Genome assembly of the basket willow, *Salix viminalis*, reveals earliest stages of sex chromosome expansion. *BMC Biology* **2020**, *18*, 78.
105. Wei, S.; Yang, Y.; Yin, T. The chromosome-scale assembly of the willow genome provides insight into Salicaceae genome evolution. *Hortic. Res.* **2020**, *7*, 45.
106. Wang, L.; Yu, S.; Tong, C.; Zhao, Y.; Liu, Y.; Song, C.; Zhang, Y.; Zhang, X.; Wang, Y.; Hua, W., *et al.* Genome sequencing of the high oil crop sesame provides insight into oil biosynthesis. *Genome Biol.* **2014**, *15*, R39.
107. Zhang, G.; Liu, X.; Quan, Z.; Cheng, S.; Xu, X.; Pan, S.; Xie, M.; Zeng, P.; Yue, Z.; Wang, W., *et al.* Genome sequence of foxtail millet (*Setaria italica*) provides insights into grass evolution and biofuel potential. *Nat. Biotechnol.* **2012**, *30*, 549-554.
108. Thielen, P.M.; Pendleton, A.L.; Player, R.A.; Bowden, K.V.; Lawton, T.J.; Wisecaver, J.H. Reference genome for the highly transformable *Setaria viridis* ME034V. *G3 (Bethesda)* **2020**, *10*, 3467-3478.
109. The Tomato Genome Consortium. The tomato genome sequence provides insights into fleshy fruit evolution. *Nature* **2012**, *485*, 635.
110. Bolger, A.; Scossa, F.; Bolger, M.E.; Lanz, C.; Maumus, F.; Tohge, T.; Quesneville, H.; Alseekh, S.; Sørensen, I.; Lichtenstein, G., *et al.* The genome of the stress-tolerant wild tomato species *Solanum pennellii*. *Nat. Genet.* **2014**, *46*, 1034-1038.
111. Xu, X.; Pan, S.; Cheng, S.; Zhang, B.; Mu, D.; Ni, P.; Zhang, G.; Yang, S.; Li, R.; Wang, J., *et al.* Genome sequence and analysis of the tuber crop potato. *Nature* **2011**, *475*, 189-195.
112. Paterson, A.H.; Bowers, J.E.; Bruggmann, R.; Dubchak, I.; Grimwood, J.; Gundlach, H.; Haberer, G.; Hellsten, U.; Mitros, T.; Poliakov, A., *et al.* The *Sorghum bicolor* genome and the diversification of grasses. *Nature* **2009**, *457*, 551-556.
113. Xu, C.; Jiao, C.; Sun, H.; Cai, X.; Wang, X.; Ge, C.; Zheng, Y.; Liu, W.; Sun, X.; Xu, Y., *et al.* Draft genome of spinach and transcriptome diversity of 120 *Spinacia* accessions. *Nat. Commun.* **2017**, *8*, 15275.
114. Cheng, S.; van den Bergh, E.; Zeng, P.; Zhong, X.; Xu, J.; Liu, X.; Hofberger, J.; de Bruijn, S.; Bhide, A.S.; Kuelahoglu, C., *et al.* The *Tarenaya hassleriana* genome provides insight into reproductive trait and genome evolution of crucifers. *Plant Cell* **2013**, *25*, 2813-2830.
115. Argout, X.; Salse, J.; Aury, J.-M.; Guiltinan, M.J.; Droc, G.; Gouzy, J.; Allegre, M.; Chaparro, C.; Legavre, T.; Maximova, S.N., *et al.* The genome of *Theobroma cacao*. *Nat. Genet.* **2010**, *43*, 101.

116. Ling, H.-Q.; Zhao, S.; Liu, D.; Wang, J.; Sun, H.; Zhang, C.; Fan, H.; Li, D.; Dong, L.; Tao, Y., *et al.* Draft genome of the wheat A-genome progenitor *Triticum urartu*. *Nature* **2013**, *496*, 87.
117. Kang, Y.J.; Satyawan, D.; Shim, S.; Lee, T.; Lee, J.; Hwang, W.J.; Kim, S.K.; Lestari, P.; Laosatit, K.; Kim, K.H., *et al.* Draft genome sequence of adzuki bean, *Vigna angularis*. *Sci. Rep.* **2015**, *5*, 8069.
118. Kang, Y.J.; Kim, S.K.; Kim, M.Y.; Lestari, P.; Kim, K.H.; Ha, B.-K.; Jun, T.H.; Hwang, W.J.; Lee, T.; Lee, J., *et al.* Genome sequence of mungbean and insights into evolution within *Vigna* species. *Nat. Commun.* **2014**, *5*, 5443.
119. Lonardi, S.; Muñoz-Amatriaín, M.; Liang, Q.; Shu, S.; Wanamaker, S.I.; Lo, S.; Tanskanen, J.; Schulman, A.H.; Zhu, T.; Luo, M.-C., *et al.* The genome of cowpea (*Vigna unguiculata* [L.] Walp.). *Plant J.* **2019**, *98*, 767-782.
120. Massonnet, M.; Cochetel, N.; Minio, A.; Vondras, A.M.; Lin, J.; Muyle, A.; Garcia, J.F.; Zhou, Y.; Delledonne, M.; Riaz, S., *et al.* The genetic basis of sex determination in grapes. *Nature Communications* **2020**, *11*, 2902.
121. Girollet, N.; Rubio, B.; Lopez-Roques, C.; Valière, S.; Ollat, N.; Bert, P.-F. *De novo* phased assembly of the *Vitis riparia* grape genome. *Sci. Data* **2019**, *6*, 127.
122. The French–Italian Public Consortium for Grapevine Genome Characterization. The grapevine genome sequence suggests ancestral hexaploidization in major angiosperm phyla. *Nature* **2007**, *449*, 463.
123. Schnable, P.S.; Ware, D.; Fulton, R.S.; Stein, J.C.; Wei, F.; Pasternak, S.; Liang, C.; Zhang, J.; Fulton, L.; Graves, T.A., *et al.* The B73 maize genome: Complexity, diversity, and dynamics. *Science* **2009**, *326*, 1112-1115.
124. Liu, M.-J.; Zhao, J.; Cai, Q.-L.; Liu, G.-C.; Wang, J.-R.; Zhao, Z.-H.; Liu, P.; Dai, L.; Yan, G.; Wang, W.-J., *et al.* The complex jujube genome provides insights into fruit tree biology. *Nat. Commun.* **2014**, *5*, 5315.
